# Selective activation of STAT3 and STAT5 dictates the fate of myeloid progenitor cells

**DOI:** 10.1038/s41420-023-01575-y

**Published:** 2023-07-28

**Authors:** Meichao Zhang, Yiling Meng, Yingxia Ying, Pingting Zhou, Suning Zhang, Yong Fang, Yuan Yao, Dong Li

**Affiliations:** 1 Department of Radiation Oncology, Shanghai Ninth People’s Hospital, Shanghai Jiaotong University School of Medicine, Shanghai, China; 2 Department of Emergency, Shanghai Ninth People’s Hospital, Shanghai Jiaotong University School of Medicine, Shanghai, China; 3 Department of Burns and Plastic Surgery, Shanghai Ninth People’s Hospital, Shanghai Jiaotong University School of Medicine, Shanghai, China

**Keywords:** Haematopoietic stem cells, Cell growth

## Abstract

The molecular programs that govern the directed differentiation of myeloid progenitor cells are still poorly defined. Using a previously established immortalized, phenotypically normal myeloid progenitor cell model mEB8-ER, we unveil a new mechanism mediated by STAT5 and STAT3 at a bifurcation point of myeloid progenitor cell-fate specification. We find that myeloid progenitor cells can spontaneously differentiate into neutrophils with a basal level of STAT3 phosphorylation, which is enhanced by G-CSF treatment or STAT3 over-expression, leading to elevated neutrophil differentiation. Reduced STAT3 phosphorylation caused by GM-CSF treatment, STAT3 specific inhibitor, or STAT3 depletion leads to attenuated myeloid differentiation into neutrophils, while elevating differentiation into monocytes/macrophages. In contrast, STAT5 appears to have an antagonistic function to STAT3. When activated by GM-CSF, STAT5 promotes myeloid differentiation into monocytes/macrophages but inhibits neutrophil differentiation. At the mechanistic level, GM-CSF activates STAT5 to up-regulate SOCS3, which attenuates STAT3 phosphorylation and consequently neutrophil differentiation, while enhancing monocyte/macrophage differentiation. Furthermore, inhibition of STAT5 and STAT3 in primary myeloid progenitors recapitulates the results from the mEB8-ER model. Together, our findings provide new mechanistic insights into myeloid differentiation and may prove useful for the diagnosis and treatment of diseases related to abnormal myeloid differentiation.

## Introduction

Neutrophils and macrophages are important components of innate immunity and play important roles in immune defense, regulation, and surveillance [1–4]. Numerous studies have demonstrated that neutrophils and macrophages are differentiated from myeloid progenitor cells and that abnormal myeloid differentiation leads to a series of diseases [5, 6]. The induction of myeloid progenitor cell differentiation requires the regulation of multiple cytokines. Among them, granulocyte colony-stimulating factor (G-CSF) and granulocyte-macrophage colony-stimulating factor (GM-CSF) are the most crucial [7, 8] and support the proliferation, survival, and differentiation of the myeloid progenitor cells.

To study the underlying molecular program of myeloid cell differentiation, we previously established a phenotypically normal, immortalized myeloid progenitor cell line mEB8-ER, which was established from mouse embryonic stem cells transduced with β-estradiol-inducible HoxB8 [9]. When β-estradiol was removed, G-CSF and GM-CSF can induce them to differentiate into neutrophils and macrophages, respectively, reminiscent of primary myeloid progenitor cells [9, 10]. The mEB8-ER cell model facilitates the dissection of the underlying molecular programs governing the directed differentiation of myeloid progenitor cells, which are still poorly defined.

It is well known that G-CSF and GM-CSF bind to the extracellular portion of their respective receptors and activate the JAK-STAT signaling pathway [11–16], which has a broad function in the regulation of cell proliferation, differentiation, apoptosis, and immune responses [13, 14, 17]. Conversely, the misregulation of STATs leads to the development of various diseases, including lymphoma, leukemia, and solid tumors, while STAT3 and STAT5 are the most common signaling proteins that are constitutively activated in leukemia [13, 18, 19].

STAT3 and STAT5 are activated by G-CSF and GM-CSF through binding to their respective receptors [13]. It has been previously shown that selective activation of STAT3 and STAT5 can differentially regulate the lineage specification of blood cells such as CD11C^+^ dendritic cells, red blood cells, and megakaryocyte [20–22]. However, it is still unclear whether they play similar roles in the directed differentiation of the myeloid progenitor cells. Inactivation of STAT3 due to mutations in the G-CSF receptor or within its gene causes myeloid progenitor cells unable to differentiate into mature neutrophils [23–25]. These findings suggest that STAT3 functions positively in the differentiation and maturation of neutrophils. However, the mice lacking STAT3 in hematopoietic cells exhibit granulocytosis instead of neutropenia, while the hematopoietic cells respond more strongly to G-CSF [26, 27], indicating that STAT3 might play a negative regulatory role in neutrophil differentiation. It was previously reported that mutations in the α subunit of the GM-CSF receptor render the cells unable to activate STAT5 and fail to differentiate into mature macrophages with the induction of GM-CSF [16]. A separate study showed that myelopoiesis appears relatively unaffected in mice with STAT5 deletion. Instead, the loss of STAT5 leads to an abnormal increase in the number and function of alveolar macrophages and, subsequently, lung injury [28]. As a result, conflicting perspectives exist regarding the roles of STAT3 and STAT5 in the differentiation of myeloid progenitor cells, necessitating further exploration and investigation.

In this study, we conducted an assessment of the lineage specification of myeloid cells. We utilized the mEB8-ER myeloid cell model and employed specific inhibitors, gene knockdown, and overexpression techniques to investigate the roles of STAT3 and STAT5. Our investigation led to the identification of a novel mechanism occurring at a critical point in myeloid progenitor cell fate determination. Our findings revealed that the activation of STAT3 promotes the differentiation of myeloid progenitor cells into neutrophils. In contrast, the activation of STAT5, induced by GM-CSF, triggers an up-regulation of SOCS3, which subsequently inhibits STAT3 activity. This inhibition of STAT3 leads to the specification of the monocyte/macrophage lineage.

## Results

### The cellular profile of the myeloid progenitor cell differentiation

We previously demonstrated that immortalized myeloid progenitor-like mEB8-ER cells can be induced to differentiate into neutrophils and macrophages by the addition of G-CSF and GM-CSF, respectively. Here, we investigated the dynamic changes in the cellular profile when β-estradiol alone was removed or when 2 ng/mL of G-CSF or GM-CSF was added simultaneously.

**Fig. 1:**
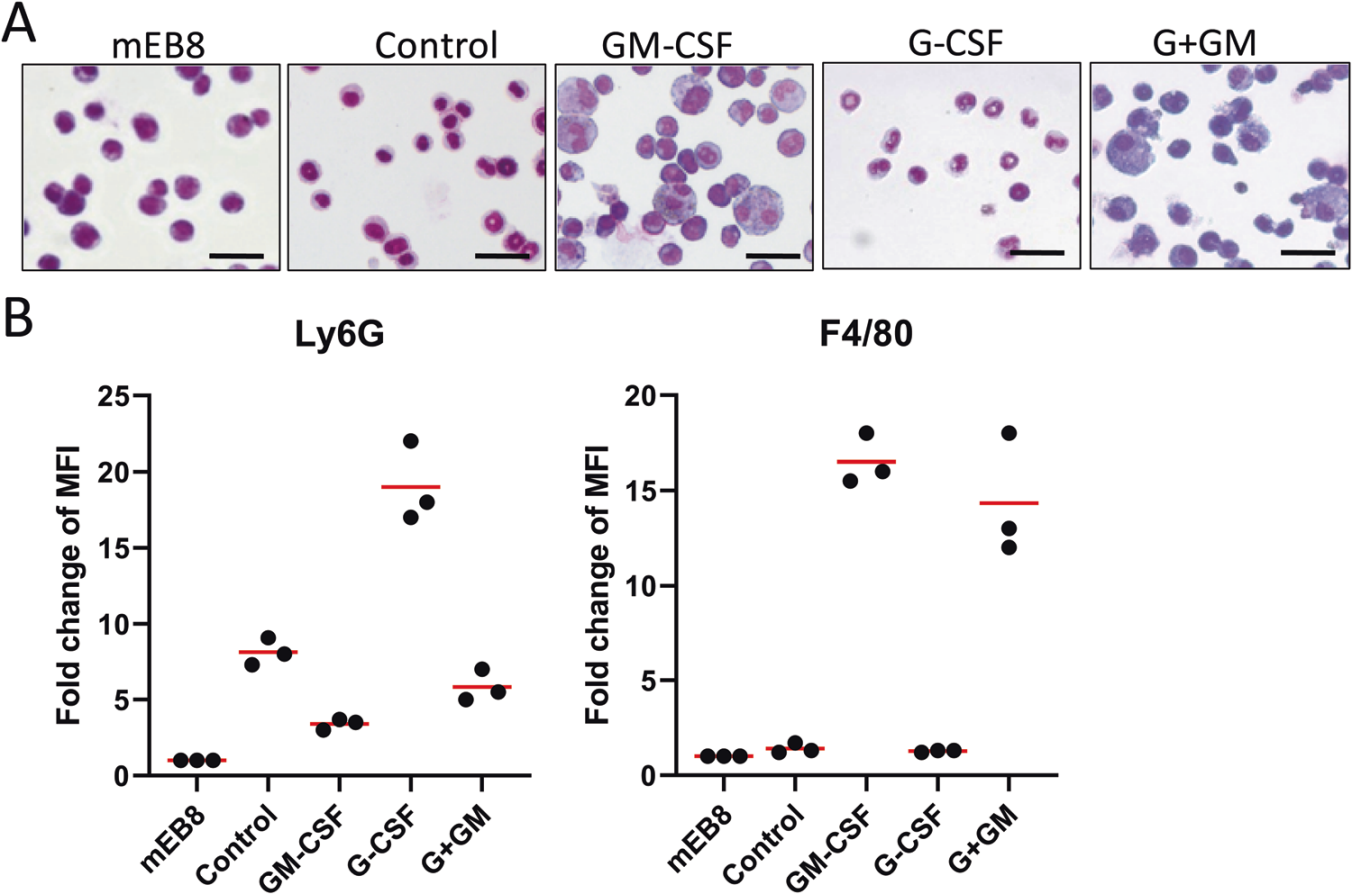
The differentiation profiles of the myeloid progenitor cells (mEB8-ER cells). Firstly, the β-estradiol was removed from the medium for mEB8-ER cells. Then the cells were differentiated without (Control) or with the induction of 2 ng/mL G-CSF and/or GM-CSF for 5 days. **A** The mEB8-ER cells or the differentiated cells were stained with Wright-Giemsa and photographed with a microscope. The bar stands for 25 μm. **B** The expression level of Ly6G and F4/80 was detected by flow cytometry.

After differentiation was induced under the aforementioned conditions for 5 days, cells were stained with Wright-Giemsa and were subsequently classified into immature neutrophils, mature neutrophils, monocytes, and macrophages based on cellular morphology and nuclear characteristics [29, 30] (Fig. S1). We found that without the induction of G-CSF or GM-CSF, most of the mEB8-ER cells were differentiated into neutrophils and mainly to the stage of immature neutrophils. When G-CSF was added, the differentiation into neutrophils was enhanced, and the percentage of mature neutrophils was markedly elevated. In contrast, with GM-CSF induction, the percentage of neutrophils in differentiated cells was dramatically reduced, while most of the cells were differentiated into monocytes and macrophages (Fig. 1A). We assessed the level of the neutrophil surface marker Ly6G and the macrophage surface marker F4/80 using flow cytometry. Consistent with the morphological responses, the level of Ly6G was elevated when the cells were differentiated with the removal of β-estradiol alone and were much higher in the presence of G-CSF. Upon GM-CSF induction, however, the level of Ly6G was reduced, while the level of F4/80 was elevated (Fig. 1B). Thus, the mEB8-ER cells can differentiate into neutrophils with the removal of β-estradiol alone, while G-CSF enhances the differentiation process. In contrast, GM-CSF prevents mEB8-ER cell differentiation to neutrophils and instead induces differentiation to monocytes and macrophages.

### Selective activation of STAT3 and STAT5 by G-CSF and GM-CSF

**Fig. 2:**
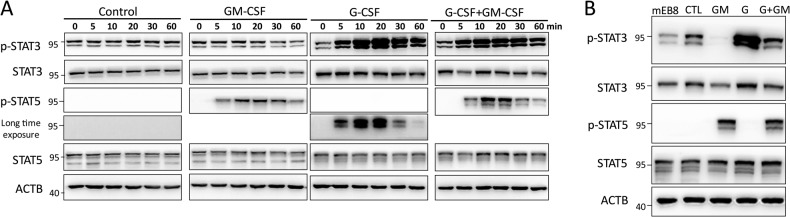
G-CSF and GM-CSF selectively activate STAT3 and STAT5, respectively. First, the β-estradiol was removed from the medium for mEB8-ER cells. Then the cells were stimulated without (Control, CTL) or with the induction of 2 ng/mL G-CSF and/or GM-CSF for 0, 5, 10, 20, 30, 60 min (**A**), and 24 h (**B**), and collected to detect the indicated protein markers. The exposure of p-STAT5 is 15 s for short exposure and 90 s for long exposure under the condition of CTL or G-CSF (**A**).

G-CSF reportedly activates STAT3 through the phosphorylation of tyrosine residues in human mononuclear cells [31]. It was also reported that in HL60 cells, GM-CSF activates the STAT5 signaling pathway [32]. To ask whether G-CSF and GM-CSF also activate the STAT molecules in myeloid progenitor cells, we stimulated the mEB8-ER cells with G-CSF and/or GM-CSF for various periods (0, 5, 10, 20, 30, and 60 min) and assessed the changes in the phosphorylation levels of STAT3 and STAT5 (Fig. 2A, B).

We detected a basal level of phosphorylation of STAT3, but not STAT5, in mEB8-ER cells with the removal of β-estradiol. However, the basal phosphorylation level of STAT3 was reduced after 30-min induction of GM-CSF (Fig. 2A). The phosphorylation level of STAT3 started to increase 5 min after G-CSF stimulation and reached the highest level at 20 min, which was sustained even after 60 min. When both G-CSF and GM-CSF were applied for >20 min, the high phosphorylation level of STAT3 was lower than with G-CSF treatment alone (Fig. 2A). GM-CSF, but not G-CSF, caused the phosphorylation level of STAT5 to increase after 5-min stimulation. STAT5 phosphorylation reached the highest level at 10 min, which was sustained even after 30–60 min of stimulation (Fig. 2A; the phosphorylation signal of STAT5 under G-CSF induction was only visible after prolonged exposure).

When the experiment was extended to 24 h, there was continuous basal level of phosphorylation of STAT3. After 24-h treatment of G-CSF or GM-CSF respectively, the phosphorylation of STAT3 or STAT5 was maintained at high levels (Fig. 2B). When the cells were treated with both G-CSF and GM-CSF, the level of STAT3 phosphorylation was lower than that of G-CSF single treatment, while STAT5 phosphorylation was largely unaltered when compared with that of GM-CSF single treatment (Fig. 2B). Thus, G-CSF and GM-CSF selectively activate STAT3 and STAT5 respectively, while GM-CSF inhibits the phosphorylation of STAT3.

### STAT3 promotes neutrophil differentiation but inhibits monocyte/macrophage differentiation

**Fig. 3:**
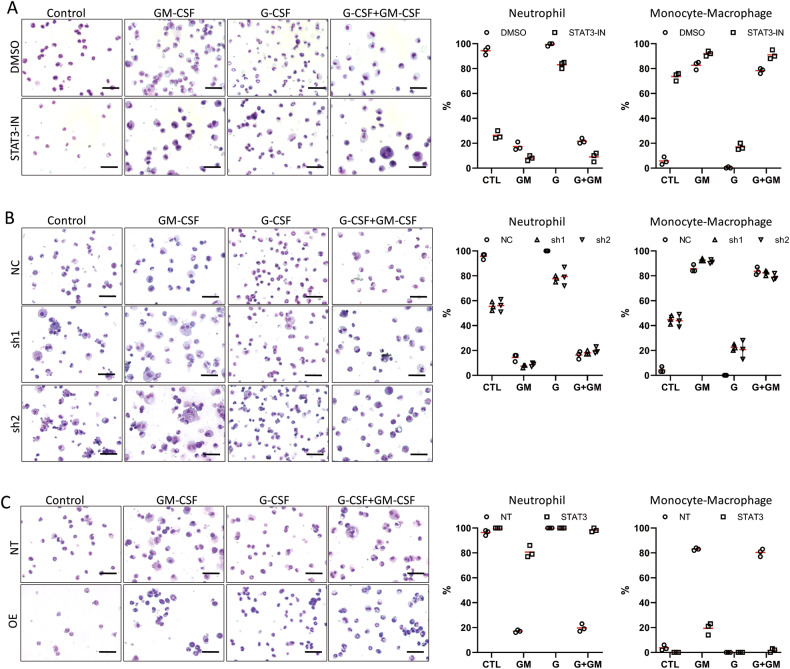
STAT3 promotes neutrophil differentiation. The mEB8-ER cells were pretreated with 5 μM STAT3-IN or DMSO (**A**), or infected by retroviral particles containing *stat3*-shRNA (**B**) or containing *stat3* over-expressing sequence (**C**). Then, the β-estradiol was removed from the medium for mEB8-ER cells, and the cells were differentiated without (Control, CTL) or with the induction of 2 ng/mL G-CSF and/or GM-CSF for 5 days. The differentiated cells were stained with Wright-Giemsa and photographed with a microscope (left). The bar stands for 50 μm. The cell number of the indicated types was counted (right). The details of statistical analyses on cell numbers in indicated types were shown in Fig. S3.

We next examined the cellular function of selective activation of STAT3 and STAT5 by G-CSF and GM-CSF. We first used STAT3-IN-1 (denoted as STAT3-IN), a specific inhibitor of STAT3 [33], to inhibit the activity of STAT3 and assessed the effects on STAT3 and STAT5. We found that the phosphorylation of STAT3 was inhibited in a concentration-dependent manner, while the phosphorylation of STAT5 was largely unaltered (Fig. S2A). We then applied STAT3-IN (5 μM) during the differentiation of mEB8-ER cells. When the cells were differentiated with the removal of β-estradiol alone or induced with G-CSF for 5 days, the percentage of total neutrophils and mature neutrophils in the STAT3-IN group was much lower than that in the DMSO control group. In contrast, the percentage of monocytes/macrophages was much higher (Figs. 3A, S3A). The treatment of the STAT3-IN cells with GM-CSF or with G-CSF and GM-CSF in combination led to a decrease in the number of neutrophils and an increase in the number of macrophages that exhibit mature morphologic features (Figs. 3A, S3A). Thus, inhibition of STAT3 decreases mEB8-ER cell differentiation into neutrophils while promoting differentiation into monocytes/macrophages.

To verify the results from STAT3 inhibition, we depleted STAT3 in mEB8-ER cells. Two shRNA sequences were found to effectively deplete STAT3 (Fig. S2C). The mEB8-ER cells infected with retroviral particles containing the shRNA sequences were selected for knockdown and differentiated for 5 days. Consistent with the effects of STAT3 inhibition, STAT3 depletion caused the percentage of total neutrophils and mature neutrophils to reduce under the spontaneous condition and with G-CSF treatment, while a fraction of cells even differentiated into monocytes/macrophages. Furthermore, with the induction of GM-CSF, STAT3 depletion increased the percentage of macrophages with mature morphologies (Figs. 3B, S3B). Thus, STAT3 depletion mimics the effect of STAT3 inhibition.

To further examine the function of STAT3, we over-expressed STAT3 in mEB8-ER cells and assessed the effects. The efficacy of STAT3 over-expression was seen using Western blotting (Fig. S2D). Both the control (NT) and STAT3 over-expressing cells were induced to differentiate for 5 days. Under the β-estradiol removal or G-CSF-induced differentiation conditions, the percentage of mature neutrophils was elevated, and the degree of differentiation was more advanced in the cells with over-expression. STAT3 overexpression also caused the percentage of total monocytes/macrophages to decrease upon GM-CSF induction, while most of the cells differentiated into neutrophils (Figs. 3C, S3C). Thus, the over-expression of STAT3 promotes directed differentiation of mEB8-ER cells into neutrophils and inhibits differentiation into monocytes/macrophages.

Together, we present evidence for STAT3 as a key regulatory factor for bifurcate differentiation of the myeloid progenitor cells. STAT3, when activated, promotes myeloid cell differentiation into neutrophils and inhibits the differentiation into monocyte/macrophages.

### STAT5 promotes myeloid differentiation to monocytes/macrophages but inhibits neutrophil differentiation

**Fig. 4:**
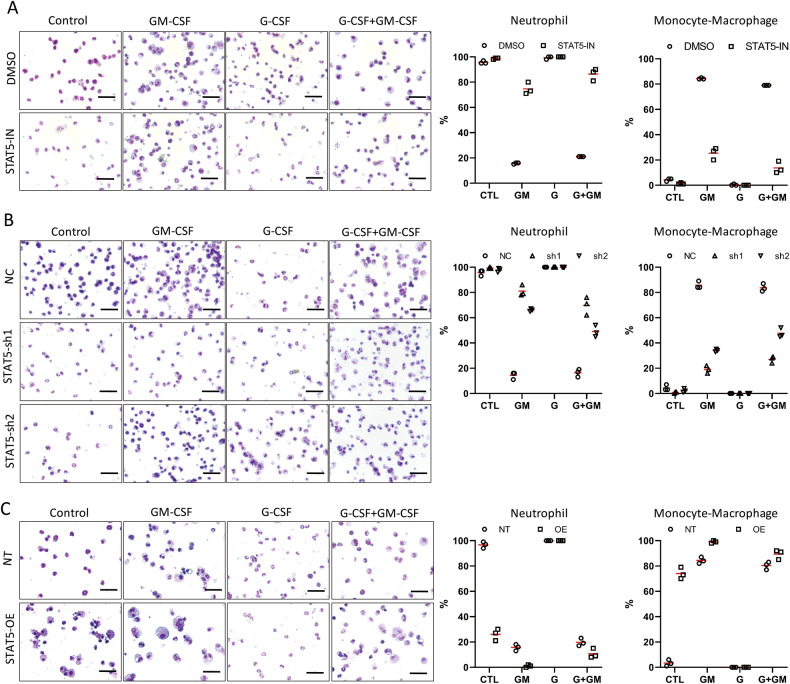
STAT5 promotes monocyte/macrophage differentiation and inhibits neutrophil differentiation. The mEB8-ER cells, pretreated with 5 μM STAT5-IN or DMSO (**A**), or infected by retroviral particles containing *stat5*-shRNA (**B**) or containing *stat5* over-expressing sequences (**C**). Then, the β-estradiol was removed from the medium for mEB8-ER cells, and the cells were differentiated without (Control, CTL) or with the induction of 2 ng/mL G-CSF and/or GM-CSF for 5 days. The cells were stained with Wright-Giemsa and photographed with a microscope (left). The bar stands for 50 μm. The cell number of the indicated types was counted (right). The details of statistical analyses on cell numbers in indicated types were shown in Fig. S4.

After assessing the function of STAT3, we next investigated the role of STAT5 in myeloid differentiation. We used STAT5-IN-2 (denoted as STAT5-IN), a specific inhibitor of STAT5 [34], to inhibit the activity of STAT5. As expected, STAT5-IN inhibits the phosphorylation of STAT5 in a concentration-dependent manner (Fig. S2B). The concentration of 5 μM was chosen for the experiments described hereafter (Fig. S2B). STAT5-IN treatment caused the percentage of mature neutrophils to increase under the β-estradiol removal or G-CSF-induced differentiation conditions (Figs. 4A, S4A; 5 days after the induction). In contrast, with the induction of GM-CSF, the same STAT5-IN treatment led to fewer macrophages and total monocytes/macrophages, while most of the cells differentiated into neutrophils (with more mature neutrophils) (Figs. 4A, S4A). Thus, STAT5 inhibition decreases the differentiation of mEB8-ER cells into monocytes/macrophages and enhances neutrophil differentiation instead.

Next, we depleted STAT5 in mEB8-ER cells to verify the results from STAT5 inhibition and identified two separate shRNA sequences that could effectively deplete STAT5 (Fig. S2E). In keeping with the effect of STAT5 inhibition. After β-estradiol removal or G-CSF-induced differentiation, the percentage of mature neutrophils was elevated upon STAT5 depletion. With GM-CSF treatment, the percentage of total monocytes/macrophages and mature macrophages were reduced, but the percentages of total and mature neutrophils were significantly elevated after STAT5 depletion (Figs. 4B, S4B). Thus, STAT5 depletion mirrors the effect of STAT5 inhibition.

To further dissect the function of STAT5, we over-expressed STAT5a and STAT5b simultaneously in mEB8-ER cells and examined the effects. The efficacy of STAT5 over-expression was confirmed with Western blotting (Fig. S2F). With STAT5 over-expression, the percentage of total neutrophils and mature neutrophils was reduced when the cells were induced with the removal of β-estradiol alone, with most cells differentiating into monocytes/macrophages. Although the percentage of total neutrophils was largely unaltered upon G-CSF induction, the percentage of mature neutrophils was reduced with STAT5 over-expression. In contrast, the treatment of GM-CSF induced an increase in the percentage of mature macrophages with STAT5 over-expression (Figs. 4C, S4C). Thus, STAT5 over-expression inhibits the differentiation of mEB8-ER cells into neutrophils while promoting monocyte/macrophage differentiation. STAT5 exhibits an opposite function of STAT3 by promoting myeloid progenitor cell differentiation into monocytes/macrophages and simultaneously inhibiting neutrophil differentiation.

### STAT5 inhibits STAT3 phosphorylation

**Fig. 5:**
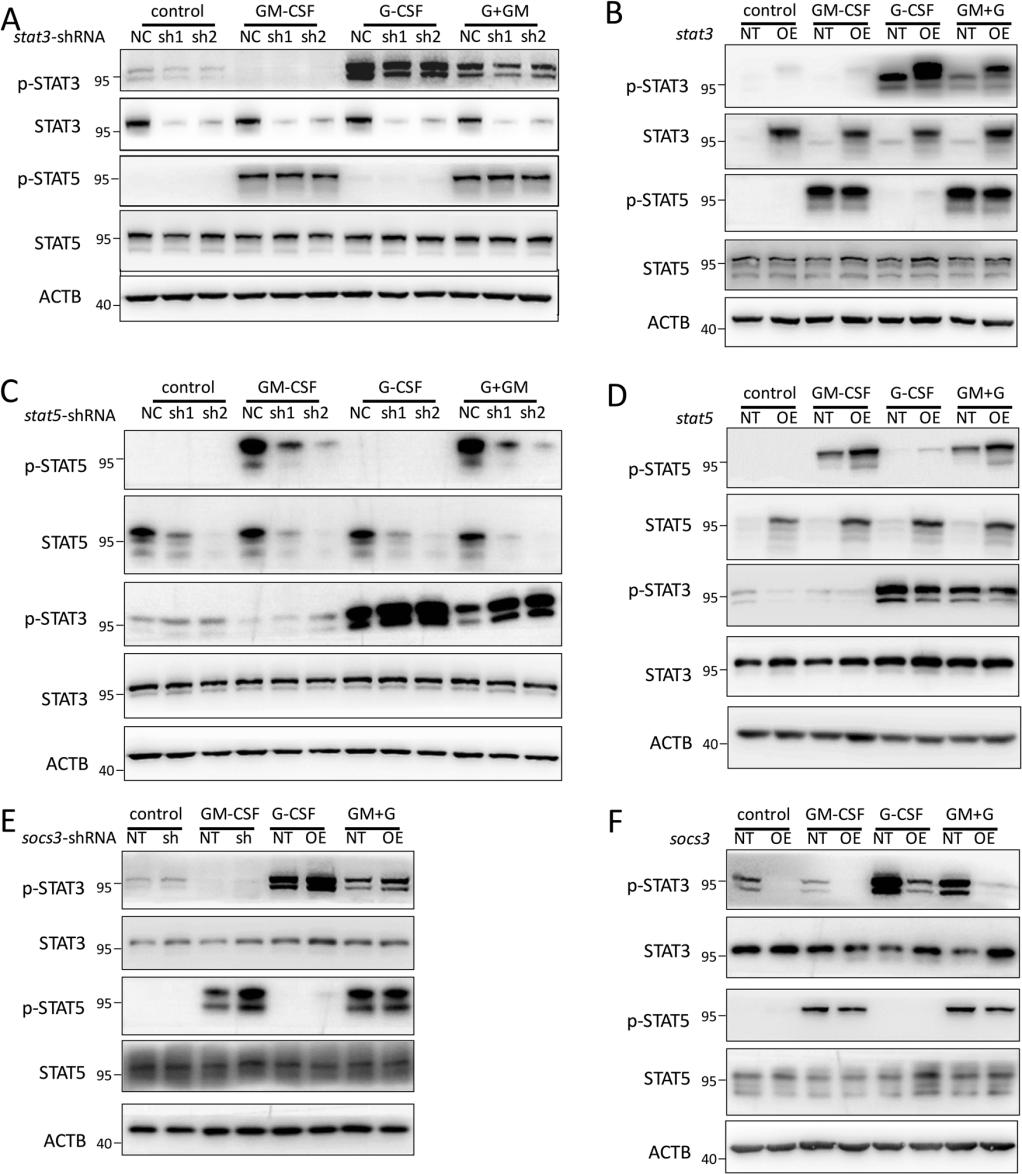
STAT5 inhibits the phosphorylation of STAT3 through SOCS3. **A**, **C** The mEB8-ER cells were infected by retroviral particles containing *stat3*-shRNA (**A**) or *stat5*-shRNA (**C**) Then, the β-estradiol was removed from the medium for mEB8-ER cells, and the cells were differentiated without (Control) or with the induction of 2 ng/mL G-CSF and GM-CSF for 24 h. The indicated protein markers were tested with Western blotting. **B**, **D** The mEB8-ER cells were infected by retroviral particles containing *stat3* (**B**) or *stat5* (**D**) over-expressing sequences. Then, the β-estradiol was removed from the the medium for mEB8-ER cells, and the cells were differentiated without (Control) or with the induction of 2 ng/mL G-CSF and GM-CSF for 24 h. The indicated protein markers were tested with Western blotting. **E**, **F** The mEB8-ER cells were infected by retroviral particles containing *socs3*-shRNA (**E**) or *socs3* over-expressing sequences (**F**). Then, the β-estradiol was removed from the medium for mEB8-ER cells, and the cells were differentiated without (Control) or with the induction of 2 ng/mL G-CSF and GM-CSF for 24 h. The indicated protein markers were tested with Western blotting.

What is the mechanism underlying the opposing function of STAT5 and STAT3? One possibility is that STAT5 and STAT3 can cross-regulate each other during mEB8-ER cell differentiation. To test this possibility, we first examined the effects of inhibiting STAT5 (STAT3) on STAT3 (STAT5). We found that inhibition of STAT3 with STAT3-IN had little impact on STAT5 phosphorylation (Fig. S2A). However, when the activity of STAT5 was inhibited with STAT5-IN (0.625–5 μM), the level of STAT3 phosphorylation was elevated (Fig. S2B). Similarly, depletion or over-expression of STAT3 had no significant effects on STAT5 phosphorylation (Fig. 5A, B), while depletion (or over-expression) of STAT5 increased (or decreased) STAT3 phosphorylation (Fig. 5C, D). These results suggest that STAT5 activation attenuates STAT3 phosphorylation.

### STAT5 inhibits STAT3 phosphorylation via SOCS3

SOCS proteins are well-known negative regulatory factors of STAT activation [13, 35]. Among them, SOCS3 is involved in the negative-feedback regulation of the JAK/STAT3 pathway and inhibits the self-activation of STAT3 [36]. Mice with SOCS3 deletion in the bone marrow exhibit STAT3 overexpression and sustained activation of the JAK/STAT3 pathway [37]. These earlier findings led us to hypothesize that SOCS3 might mediate the inhibitory effect of GM-CSF on STAT3.

To assess this, we measured the mRNA level of *socs3* during myeloid differentiation. Both G-CSF and GM-CSF treatments significantly increased the mRNA level of *socs3*, and the up-regulation of *socs3* mRNA coincided with the changes in STAT3 and STAT5 phosphorylation (Fig. S5A, Fig. 2A). Depletion of STAT5 led to a decrease in the level of SOCS3 (Fig. S5B), while over-expression of STAT5 led to an increase in the level of SOCS3 (Fig. S5C). Thus, in line with previous reports in other cells [38], STAT5 can modulate SOCS3 in myeloid progenitor cells.

We next assessed the effect of SOCS3 depletion or over-expression on STAT3 phosphorylation. SOCS3 depletion (Fig. S2G) caused the level of STAT3 phosphorylation to markedly elevate (Fig. 5E), while SOCS3 over-expression (Fig. S2H) reduced STAT3 phosphorylation (Fig. 5F). In contrast, depletion and over-expression of SOCS3 only had a moderate effect on STAT5 phosphorylation (Fig. 5E, F).

Together, these results suggest that GM-CSF activates STAT5, leading to the up-regulation of SOCS3, which, in turn, inhibits the phosphorylation of STAT3.

### SOCS3 inhibits neutrophil differentiation and promotes monocyte/macrophage differentiation

**Fig. 6:**
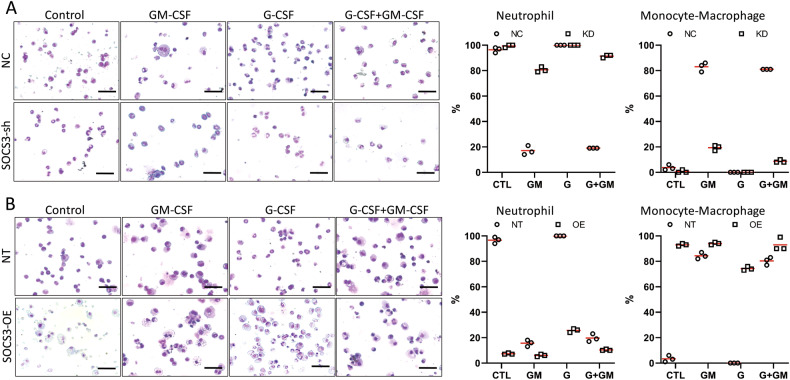
SOCS3 inhibits neutrophil differentiation but promotes monocyte/macrophage differentiation. **A**, **B** The mEB8-ER cells were infected by retroviral particles containing *socs3*-shRNA (**A**) or *socs3* over-expression sequence (**B**). Then, the β-estradiol was removed from the medium for mEB8-ER cells, and the cells were differentiated without (Control, CTL) or with the induction of 2 ng/mL G-CSF and GM-CSF for 5 days. The morphological changes were evaluated with Wright-Giemsa staining (left). The bar stands for 50 μm. The cell number of the indicated types was counted (right). The details of statistical analyses on cell numbers in indicated types were shown in Fig. S6.

How does SOCS3 influence myeloid differentiation? To address this question, we depleted or over-expressed SOCS3 in mEB8-ER cells and subsequently induced cell differentiation. SOCS3 depletion caused the percentage of mature neutrophils to increase 5 days after the removal of β-estradiol alone or G-CSF induction, while the percentages of monocytes, mature macrophages, and total monocytes/macrophages were reduced with GM-CSF induction (Figs. 6A, S6A). Expectedly, SOCS3 over-expression exerted the opposite effects. After the removal of β-estradiol alone or G-CSF induction, the numbers of total and mature neutrophils were reduced, with some cells differentiating into macrophages. When the cells were induced with GM-CSF, the number of total neutrophils was reduced, while the numbers of total monocytes/macrophages and mature macrophages were elevated (Figs. 6B, S6B). Thus, SOCS3 might function to inhibit neutrophil differentiation but promote the differentiation of myeloid progenitor cells into monocytes/macrophages.

### STAT3 and STAT5 have a conserved function in primary myeloid progenitor cells

To extend the findings from the mEB8-ER cell model to primary myeloid progenitor cells, we isolated 6-week-old mouse bone marrow-derived myeloid progenitor cells and used specific inhibitors to study the role of STAT3 and STAT5 in the differentiation of myeloid primary cells.

**Fig. 7:**
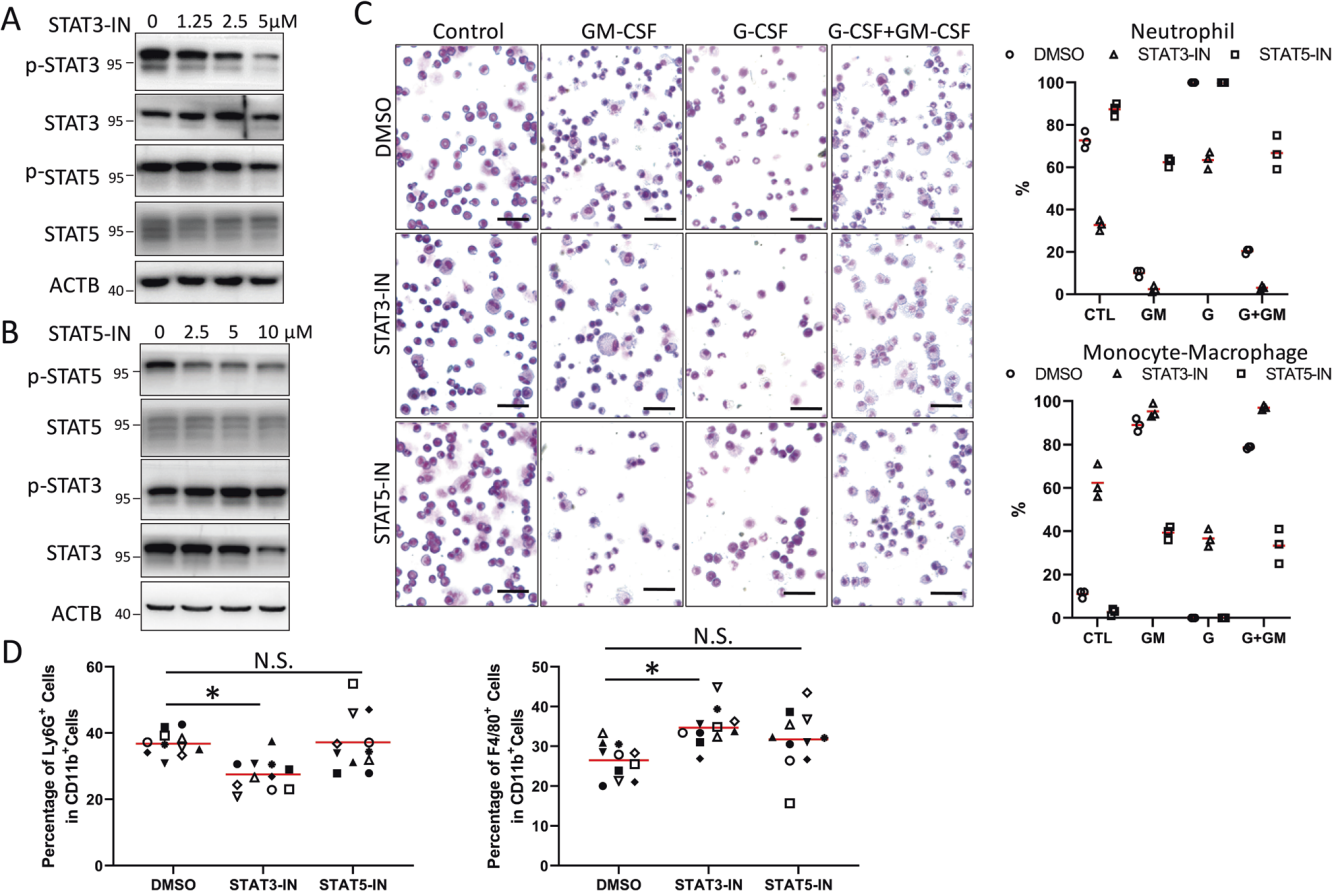
STAT3 and STAT5 have a conserved function in primary myeloid progenitor cells and in vivo. **A**, **B** The bone marrow-derived myeloid progenitor cells were isolated from 6-week-old mice. The primary cells were pretreated with STAT3-IN (A) or STAT5-IN (**B**) according to the indicated concentrations, and induced by 2 ng/mL G-CSF and GM-CSF for 24 h. The cells were collected to detect the indicated protein markers with western blotting. **C** The primary cells were pretreated with 5 μM STAT3-IN or STAT5-IN, followed by differentiation medium treatment only (Control, CTL) or were induced by 10 ng/mL G-CSF or/and GM-CSF for 5 days. The cells were stained with Wright-Giemsa (left). The bar stands for 50 μm. The cell number of the indicated types was counted (right). The details of statistical analyses on cell numbers in indicated types were shown in Fig. S7. **D** 10 mg/kg STAT3-IN or STAT5-IN were injected into the 6-week-old male mice intraperitoneally every 2 days. After 7 days, peripheral blood and bone marrow cells were separated, and the levels of CD11b, Ly6G and F4/80 were detected by flow cytometry. The data are the mean ± SD, *P < 0.05.

In primary cells, STAT3 or STAT5 inhibitors potently diminished the levels of phosphorylated STAT3 and STAT5, respectively (Fig. 7A, B). The primary cells were then pretreated with the inhibitors and induced with the removal of IL3 and IL6, or the addition of G-CSF or/and GM-CSF simultaneously for 5 days (Figs. 7C, S7C), allowing us to obtain similar results to those from mEB8-ER cells. Thus, in primary myeloid cells, STAT3 promotes neutrophil differentiation and inhibits monocyte/macrophage differentiation of myeloid progenitor cells, while STAT5 exerts an opposite function to STAT3.

### The effect of STAT3 and STAT5 inhibition in vivo

Finally, we examined the function of STAT3 and STAT5 in vivo by conducting experiments with mice. We injected STAT3-IN and STAT5-IN intraperitoneally into 6-week-old male mice (at 10 mg/kg body weight) every 2 days. Seven days after the beginning of the injection, we isolated peripheral blood cells from the mice and measured the number of CD11b^+^ Ly6G^+^ neutrophils and CD11b^+^ F4/80^+^ monocytes/macrophages by using flow cytometry. We found that the STAT3-IN treatment reduced the number of neutrophils when compared with the control group (DMSO only) while elevating the number of macrophages (Fig. 7D), in keeping with the findings from the in vitro analyses. However, the treatment of STAT5 inhibitor had no significant effect on the number of neutrophils and macrophages in mice (Figs. 7D, S8B), which may be due to the complexity of the in-vivo microenvironment and/or the low phosphorylation level of STAT5, which was difficult to detect.

## Discussion

Many cell-lineage specification processes go through multiple classic bifurcation points. The myeloid progenitor cells are also positioned at such a bifurcation point, where cells are ready to differentiate into neutrophils or monocytes/macrophages upon the stimulation of various cues. For instance, the progenitor cells can differentiate spontaneously into neutrophils, and G-CSF promotes this process. In contrast, GM-CSF inhibits neutrophil differentiation but induces the cells to differentiate into monocytes/macrophages (Fig. 1). Thus, the regulatory mechanism underlying the bifurcation point is crucial and dictates the fate determination of myeloid cells. Classical bifurcation points are mostly regulated by at least two transcription factors with antagonistic functions, such as Notch/Pax5 and C/EBP/GATA-1 [39–41]. The gradual changes in the expression of these transcription factors lead to the shift of lineage potential [41]. It was previously reported that STAT3 and STAT5 also play an important role in the regulation of several bifurcation points [20, 22, 42]. The CD34^+^ common myeloid precursor cells and common lymphoid precursor cells can pan-differentiate into CD11C^+^ dendritic cells by activating STAT3 in the presence of Flt3L. This differentiation process can be significantly enhanced by IL-6 and G-CSF, which increase the phosphorylation level of STAT3. However, the inhibition of STAT3 and the simultaneous activation of STAT5 induced by GM-CSF lead to the inhibition of pan differentiation [20, 43]. In this study, we also showed that selective activation of STAT3 and STAT5 can dictate the fate specification of myeloid progenitor cells.

When G-CSF binds to its receptor, the intracellular segments undergo auto-phosphorylation. Specifically, the phosphorylation of Tyr704 and Tyr744 sites of G-CSFR enables it to selectively bind and activate STAT3 [44]. On the other hand, the GM-CSF receptor lacks intrinsic tyrosine kinase activity but interacts with GM-CSF, leading to JAK2 auto-phosphorylation, which subsequently phosphorylates STAT5 [45, 46]. Consistent with these mechanisms, G-CSF and GM-CSF rapidly activate the JAK-STAT signaling pathway and induce phosphorylation of STAT3 and STAT5, respectively, in mouse myeloid progenitor cells. Interestingly, G-CSF promotes STAT3 phosphorylation, while GM-CSF inhibits it (Fig. 2). Inhibition of STAT3 using a specific inhibitor attenuates neutrophil differentiation but enhances myeloid differentiation into monocytes/macrophages (Fig. 3A). Subsequent experiments involving gene knockdown and overexpression independently confirm the findings observed with the inhibitor (Fig. 3B, C). These results collectively indicate that STAT3 plays a pivotal role as a key regulatory molecule that determines the divergence of myeloid differentiation towards neutrophils and macrophages.

It has been reported that SOCS3 acts as a negative regulator of STAT3 and is up-regulated when STAT3 is activated by cytokines, thereby inhibiting STAT3 in a manner of negative feedback [36]. Consistent with previous reports, we found in this study that G-CSF can activate STAT3 (Fig. 2) and significantly increases the mRNA level of *socs3* (Fig. S5A). Furthermore, experiments with SOCS3 depletion and overexpression revealed its negative regulation of STAT3 (Fig. 5E, F). As reported earlier, SOCS3 deletion causes the number of blood neutrophils in mice to markedly increase [47, 48]. Our current study also shows that SOCS3 has a negative regulatory function in neutrophil differentiation while promoting monocyte/macrophage differentiation (Fig. 6). At the mechanistic level, SOCS3 inhibits STAT3 by attenuating the phosphorylation level of STAT3. Furthermore, SOCS3 regulation of STAT3 appears specific, because modulation of SOCS3 level only has a minor effect on STAT5 (Fig. 5E, F).

In contrast, STAT5, when inhibited, induces the myeloid progenitors to undergo neutrophil differentiation while preventing monocyte/macrophage differentiation (Fig. 4A). This conclusion is supported by experiments with STAT5 knockdown and overexpression (Fig. 4B, C). Thus, STAT5 functions antagonistically to STAT3 in the differentiation of myeloid progenitor cells. At the molecular level, we found that STAT5 depletion or overexpression leads to changes in STAT3 phosphorylation (Fig. 5C, D), suggesting a potential direct regulatory mechanism for STAT5 to inhibit STAT3.

It has been reported that STAT5 can bind to the promotor of *socs3* and regulates the expression of *socs3* at the transcriptional level [49]. We extend these findings and discover that GM-CSF activates STAT5 (Fig. 2), increasing *socs3* mRNA level (Fig. S5A). When STAT5 is depleted or overexpressed, the mRNA levels of *socs3* are reduced or elevated correspondingly (Fig. S5B, C), suggesting STAT5 regulation of SOCS3. Additionally, G-CSF can also phosphorylate and activate STAT5, but the phosphorylation level of STAT5 is lower than that upon GM-CSF induction (Fig. 2), Moreover, STAT5 depletion further enhances G-CSF-induced STAT3 phosphorylation, while STAT5 overexpression reduces it (Fig. 5C, D). These results suggest that STAT5 inhibits the phosphorylation of STAT3 by upregulating SOCS3, which, as a result, leads myeloid progenitors to differentiate into monocytes/macrophages.

In conclusion, we discover a new mechanism at a bifurcation point of myeloid progenitor cell-fate specification. When G-CSF is present, STAT3 is activated, leading to neutrophil differentiation. When GM-CSF is present, STAT5 is activated, which causes SOCS3 to up-regulate, resulting in the inhibition of STAT3 and monocyte/macrophage differentiation. Our research provides new mechanistic insights into myeloid differentiation and may prove useful for the diagnosis and treatment of diseases related to abnormal myeloid differentiation.

### Limitations of the study

In this study, we utilized an estrogen-induced immortalized myeloid progenitor cell line called mEB8-ER to investigate the role of STAT3 and STAT5 in the regulation of myeloid progenitor differentiation. We employed specific inhibitors, gene knockdown, and overexpression techniques to reveal their involvement. Additionally, we validated our findings by using specific inhibitors in bone marrow-derived primary myeloid progenitors. Moreover, we conducted in vivo experiments by administering specific inhibitors via intraperitoneal injection to examine the effect on neutrophils and monocyte-macrophages in peripheral blood.

It is possible that the in vivo environment is too complex or that the inhibitory effect of the specific inhibitor of STAT5 did not produce detectable effects at the cellular level due to low phosphorylation levels in peripheral blood. Therefore, the in vivo experimental results of STAT5-specific inhibitors do not exclude the possibility of STAT5 inhibition influencing the percentage of neutrophils and monocyte-macrophages in mouse peripheral blood.

## Methods

### Cell culture

The mEB8-ER cells were cultured in Opti-Mem medium (Gibco, 11058-021) with 10% fetal bovine serum (FBS, Gibco, 1600044), 100 U/mL penicillin, 100 mg./mL streptomycin (Gibco, 15140122), 1% Gautama (Gibco, 35050061), 10 ng/mL SCF, 30 gm. mercaptothions, and 1 μM β-estradiol. The medium used for mEB8-ER cell differentiation was without β-estradiol but contained 2 ng/mL G-CSF and/or GM-CSF. HEK293T cells were cultured in DMEM (high glucose) (Gibco, C11995500CP) with 10% FBS, 100 U/mL penicillin, 100 μg/mL streptomycin, 1% GlutaMAX.

### Retroviral particles production and infection

For shRNA-mediated knockdown of *stat3*, *stat5*, and *socs3*, pSIREN-RetroQ-shRNA containing retroviral particles was used. Sequences of shRNA were as Table 1. For over-expression of *stat3*, *stat5a/b*, and *socs3*, pBABE-puro containing retroviral particles were constructed and used. The cDNAs of *stat3* (NM_011486.4), *stat5a/b* (NM_011488.3, NM_001113563.1), and *socs3* (NM_007707.3) were purchased from Nanjing Bioworld Biotechnology.

**Table 1:** The sequences of shRNA for pSIREN-RetroQ-shRNA.

Name	shRNA Sequence (5'-3')
*stat3-sh1*	GATCCGCGACTTTGATTTCAACTACAATTCAAGAGATTGTAGTTGAAATCAAAGTCGTTTTTTG
*stat3-sh2*	GATCCGCCTGAGTTGAATTATCAGCTTTTCAAGAGAAAGCTGATAATTCAACTCAGGTTTTTTG
*stat5-sh1*	GATCCGGGTTTTTGCTGAAGATCAAGCTTCAAGAGAGCTTGATCTTCAGCAAAAACCTTTTTTG
*stat5-sh2*	GATCCGCACAGAAACTGTTCAACATCATTCAAGAGATGATGTTGAACAGTTTCTGTGTTTTTTG
*socs3-sh*	GATCCGTCTTCACGTTGAGCGTCAAGATTCAAGAGATCTTGACGCTCAACGTGAAGATTTTTTG

The retroviral plasmids containing shRNA or over-expression sequences and the packaging plasmids pUMVC, pCMV-VSV-G were co-transfecting to 293 T cells using the transfection reagent Hieff Trans^®^ PEI (Shanghai Yeasen Biotechnology, # 40820ES04). Supernatants containing retroviral particles were collected after 48 h of transfection.

Retroviral particles were added along with 6 μg/mL polybrene (Shanghai Yeasen Biotechnology, #40804ES76) to the mEB8-ER cells and incubated for 20 h, after which fresh growth medium was provided. Cells were selected with 2 μg/mL puromycin (Shanghai Yeasen Biotechnology, #60210ES25) for 2 days after infection and used for subsequent analyses 3 days after selection.

### Primary progenitor cells isolated from mouse bone marrow

The primary progenitor cells were isolated from the femurs and tibias of 6 weeks old C57BL/6 mice and then used with EasySep™ Mouse Hematopoietic Progenitor Cell Isolation Kit (Stemcell Technologies, #19856) to obtain primary progenitor cells. The isolated progenitor cells were cultured in culture medium containing 90% IMDM, 10% FBS, 100 U/mL penicillin, 100 μg/mL streptomycin, 1% GlutaMAX, 10 ng/mL SCF, 20 ng/mL IL3, 20 ng/mL IL6. The differentiation medium for the primary cells was the culture medium with the removal of IL3 and IL6. For the differentiation of the primary cells, 10 ng/mL G-CSF and/or GM-CSF were added to the differentiation medium.

### Effects of STAT3/STAT5 inhibitor in vivo

C57BL/6 mice (6 weeks old) were used. 10 mg/kg STAT3-IN-1 and STAT5-IN-2 were injected intraperitoneally every 2 days. Mice were sacrificed on day 7. Peripheral blood cells were separated, and the levels of CD11b, Ly6G, and F4/80 were detected with flow cytometry.

### Western blotting

Cells were lysed in lysis buffer (20 mM Tris, pH 7, 0.5% NP-40, 250 mM NaCl, 3 mM EDTA, 3 mM EGTA, 2 mM DTT, 0.5 mM PMSF, 20 mM β-glycerol phosphate, 1 mM sodium vanadate, and 1 mg/mL of leupeptin) and were analyzed by immunoblotting after SDS-PAGE. Proteins were visualized by ECL according to the manufacturer’s instructions (Millipore, WBKLS0050).

### RNA isolation and real-time PCR

Total RNA was extracted with TRIzol reagent (Invitrogen, #15596-026) according to the manufacturer’s guidelines. The reverse transcription of RNA samples was performed with PrimeScript RT reagent Kit with gDNA Eraser (TAKARA, #RR047A). Real-Time PCR was then performed with SYBR Green Master Mix Reagent (Shanghai Yeasen Biotechnology, #11201ES03) using Roche Light Cycler480 Real-Time PCR detector (Roche Diagnostics International Ltd, Forrenstrasse2,6343 Rotkreuz, Switzerland). For SYBR Green RT-PCR, we used the following sequences:

*actb*_Fw: GGCTGTATTCCCCTCCATCG; *actb*_Rev: CCAGTTGGTAACAATGCCATGT

*socs3*_Fw: CGTGCGCCATGGTCACCC; *socs3*_Rev: GCCTCGGAGGAGAGGCGA.

### Wright-Giemsa staining

Cellular morphology was evaluated by using Wright-Giemsa staining as previously described [50]. Briefly, cytospin preparations of 2 × 10^5^ cells were incubated sequentially in solution A for 1 min and solution B for 7 min, washed with water, air-dried, and then examined under a microscope. Images were collected using a Nikon ECLIPSE Ts2R microscope (Nikon, Tokyo, Japan).

### Flow cytometry

Flow cytometry was performed according to the manufacturer’s protocol. Briefly, cells were washed with ice-cold phosphate-buffered saline (PBS, Gibco, #10010023), incubated at 4 °C for 1 h in PBS/bovine serum albumin (BSA, 0.5%) with anti-Ly6G or anti-F4/80 antibody or an isotype control. Finally, cells were washed and detected with a BD FACS CantoTM II flow cytometer (BD Bioscience, California, USA).

### Statistical analyses

Results are shown as mean ± SD. Statistical significance was determined using *t*-test. Results were considered significant when *P* < 0.05.

## Supplementary information

Meichao ZhangXs Supplementary Figures

Key resources table

Original Data File

## Data Availability

This paper does not report the original code.

## Code availability

This paper does not report original code.
