# Supplementary material for: Selective activation of STAT3 and STAT5 dictates the fate of myeloid progenitor cells
Source: Cell Death Discov. 2023 Jul 28;9:274. doi: 10.1038/s41420-023-01575-y (PMC10382539; doi:10.1038/s41420-023-01575-y)
Supplement: Supplementary file 1 — Meichao ZhangXs Supplementary Figures [file 41420_2023_1575_MOESM1_ESM.docx]

**Meichao Zhang`s Supplementary Figures**


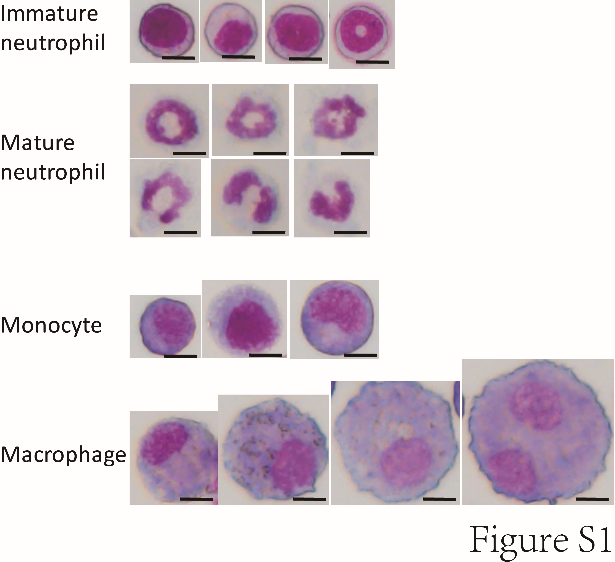


**Figure S1. The morphology of neutrophils and monocytes/macrophages.** With the removal of β-estradiol, mEB8-ER cells were induced with 2ng/mL G-CSF or GM-CSF for 5 days, and then the cells were stained with Wright-Giemsa and photographed with a microscope. Representative cells were selected as shown (Bar represents 8μm). In Wright-Giemsa-stained blood cells, the cytoplasm of neutrophils is transparent or light red, and there are small light red or purple particles that are diffusely distributed. The nuclei of mature neutrophils are ring-shaped, rod-shaped, or lobulated. Monocytes have abundant cytoplasm-stained gray-blue, and the nuclei are often kidney-shaped or horseshoe-shaped. Macrophages are larger in size, with darker oval nuclei, and inconspicuous nucleoli; some of them have two or more nuclei.


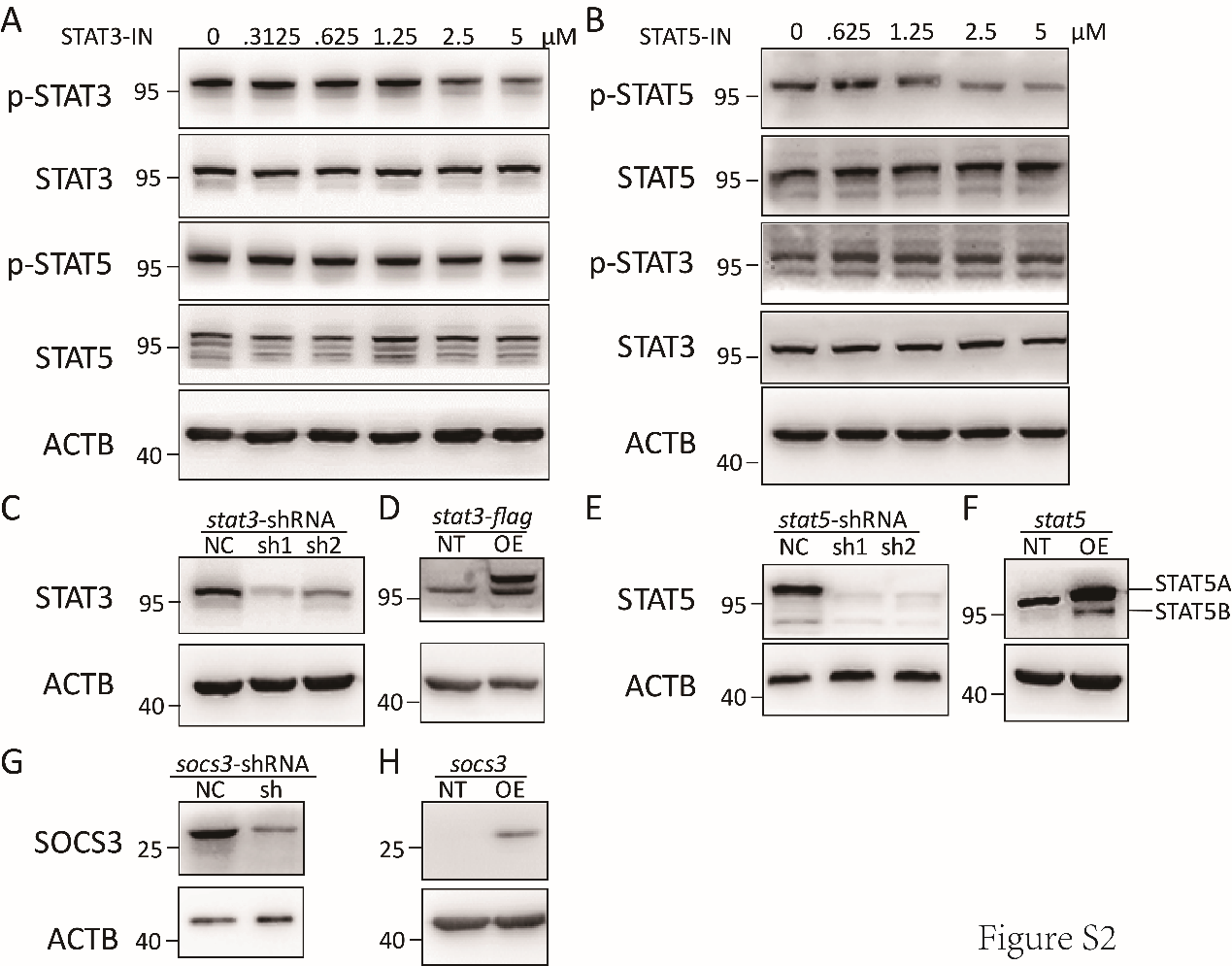


**Figure S2. The supplementary western blot results.**

(A, B) mEB8-ER cells were pretreated with STAT3-IN (A) or STAT5-IN (B) at the indicated concentrations and induced by the combined use of 2 ng/mL G-CSF and GM-CSF for 24 hours. The cells were collected to detect the indicated protein markers with Western blotting.

(C, E, G) The mEB8 cells were infected with retroviruses containing *stat3* shRNAs (C), *stat5* shRNAs (E) or *stat5* shRNAs (G), and the indicated protein markers were tested with Western blotting.

(D, F, H) The mEB8 cells were infected with retroviral particles containing *stat3* (D), *stat5* (F) or *socs5* (H), over-expressing sequences, and the indicated protein markers were tested with Western blotting.


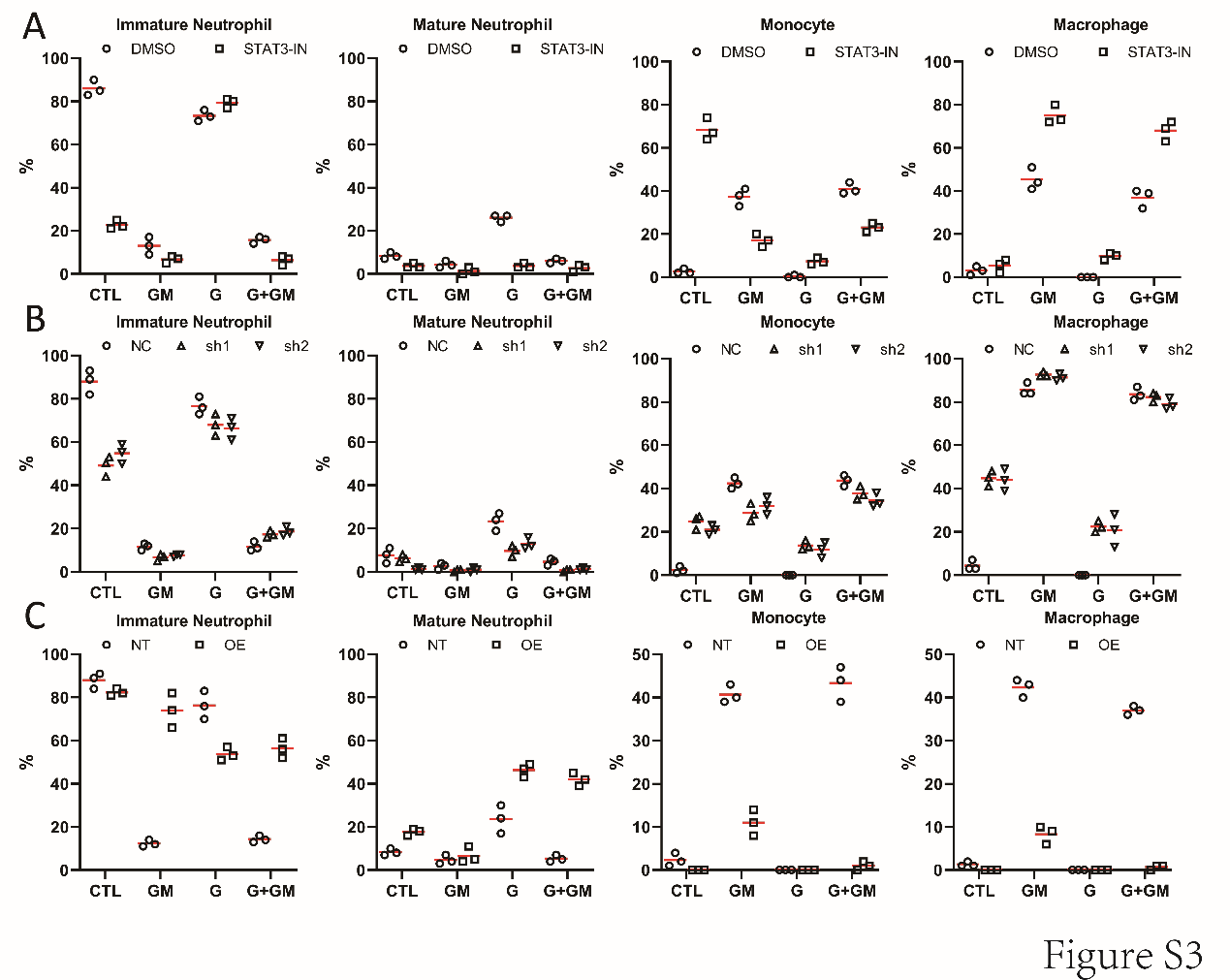


**Figure S3. Supplementary Statistical data for Figure 3.** The statistics of the results described in Figure 3 are analyzed separately for immature neutrophils, mature neutrophils, monocytes, and macrophages.


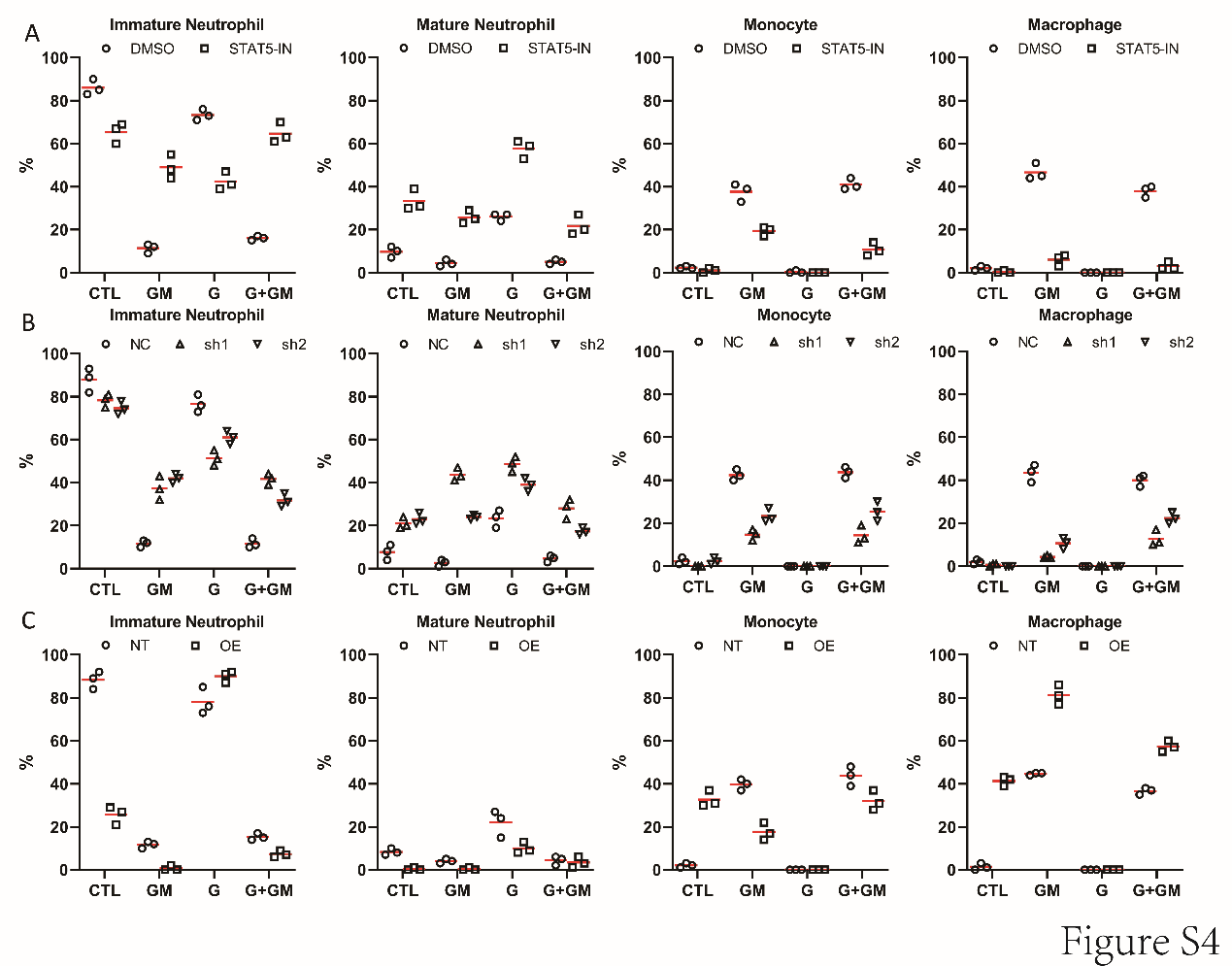


**Figure S4. Supplementary Statistical data for Figure 4.** The statistics of the results of Figure 4 are analyzed separately for immature neutrophils, mature neutrophils, monocytes, and macrophages.


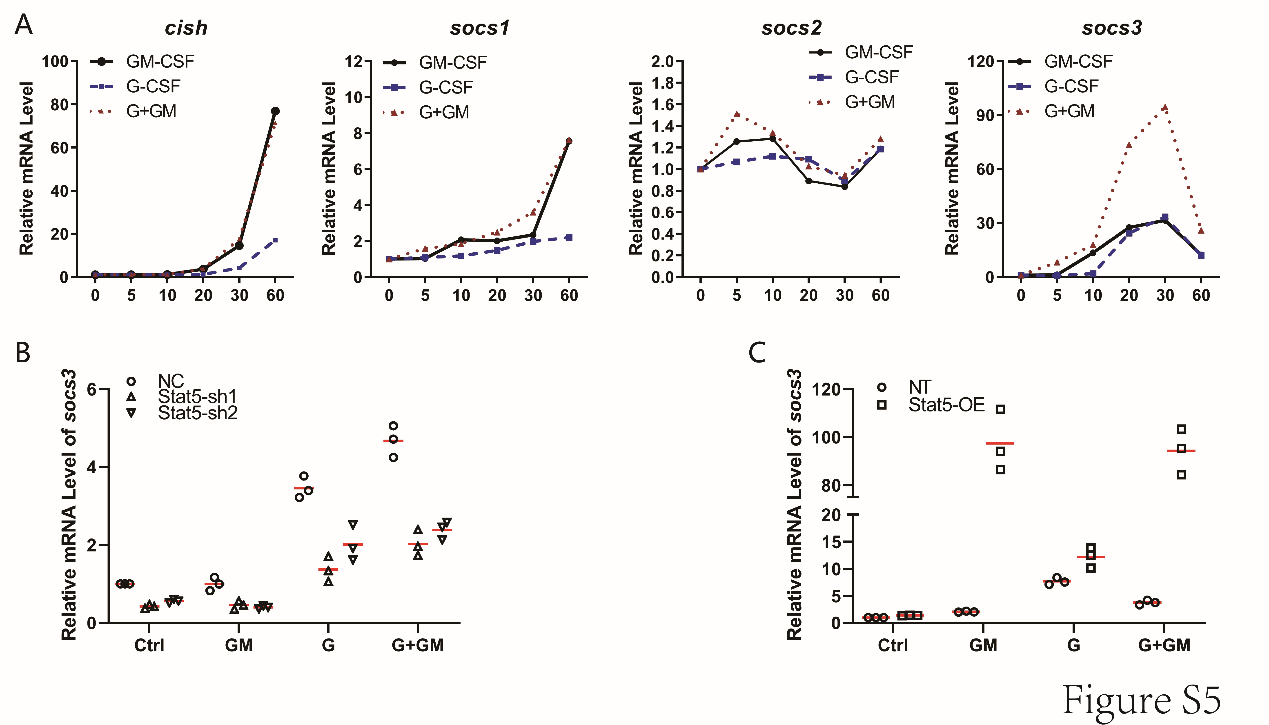


**Figure S5. The mRNA level of socs3 was regulated by STAT5.** (A) The mEB8-ER cells were stimulated by G-CSF and GM-CSF for 0, 10, 20, 30, and 60 min, and the mRNA levels of socs members were detected with qPCR. (B, C) The mEB8-ER cells were infected with retroviral particles containing stat5-shRNA (B) or stat5 over-expressing sequences (C) and were induced by 2 ng/mL G-CSF or/and GM-CSF for 24 hours. The mRNA levels of SOCS3 were detected with qPCR.


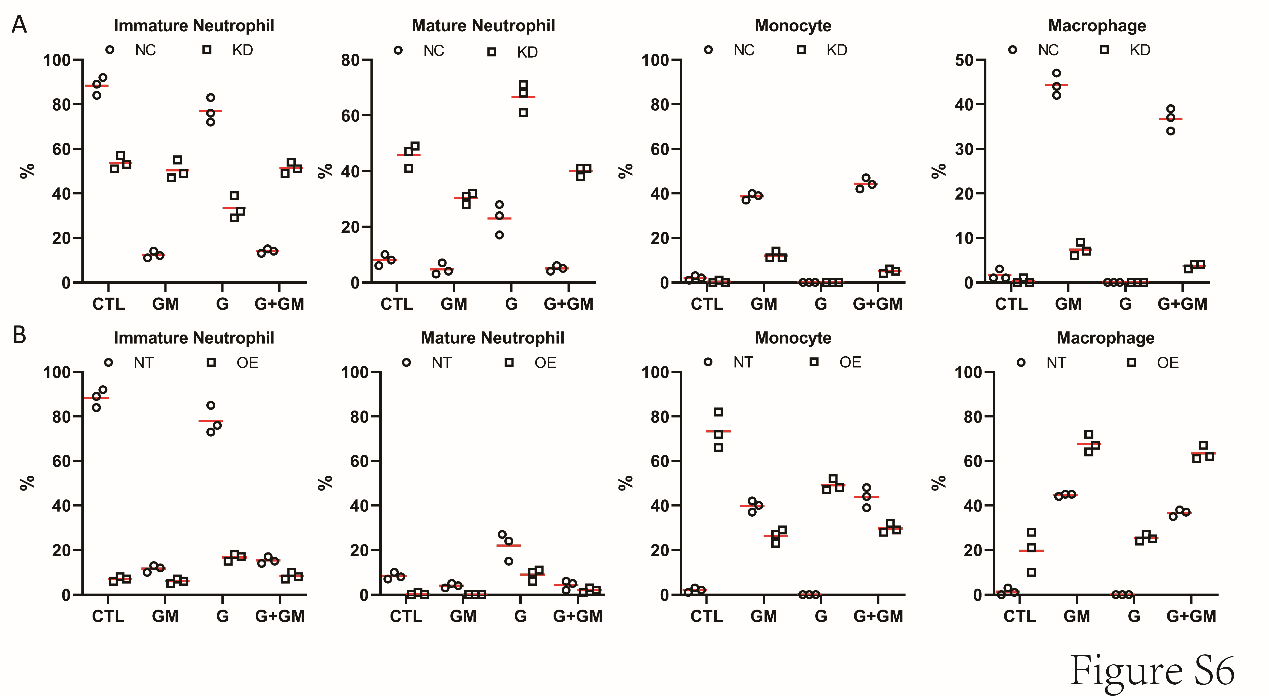


**Figure S6. Supplementary Statistical data for Figure 6.** The statistics of the results in Figure 6 are analyzed separately for immature neutrophils, mature neutrophils, monocytes, and macrophages.


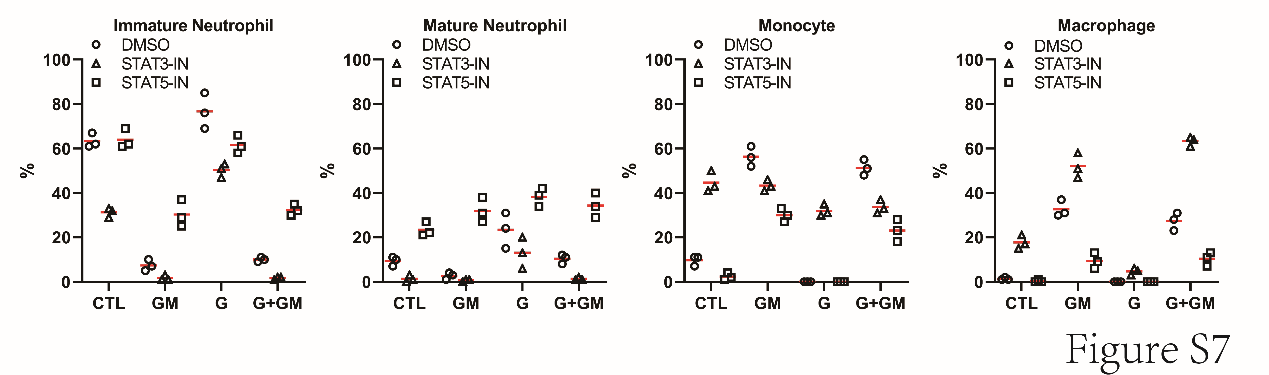


**Figure S7. Supplementary Statistical data for Figure 7C.** The statistics of the results of Figure 7C are analyzed separately for immature neutrophils, mature neutrophils, monocytes, and macrophages.


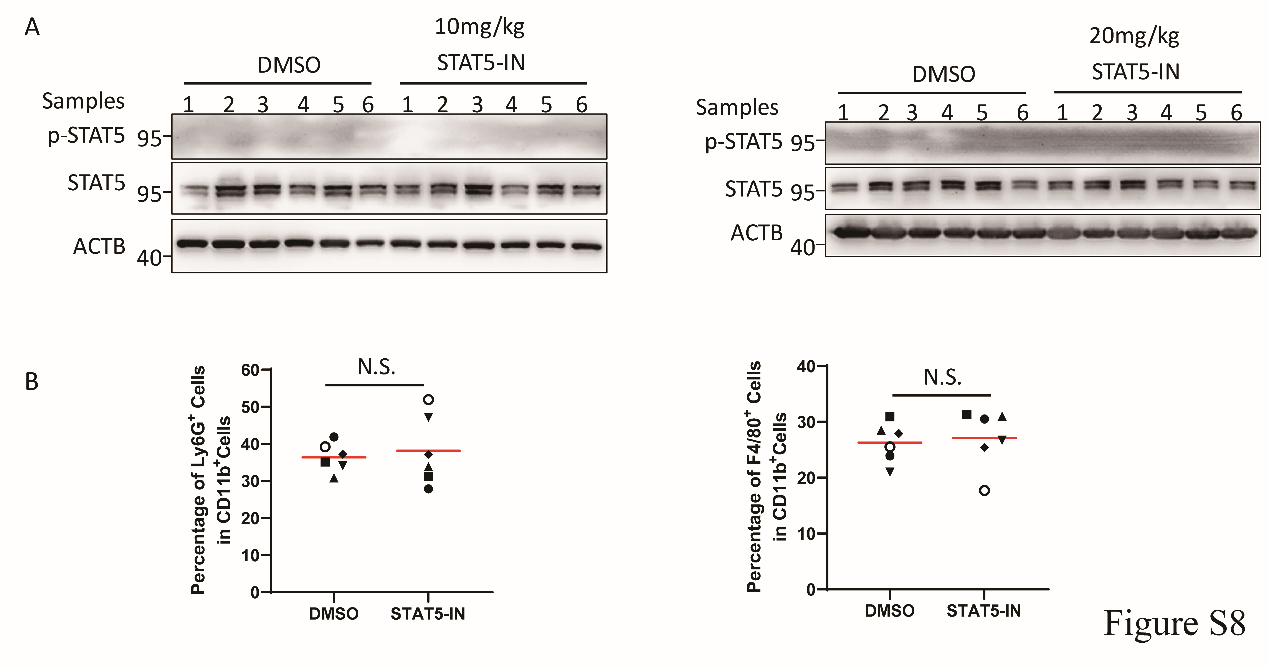


**Figure S8. The effects of STAT5-IN treatment on STAT5 phosphorylation levels and neutrophils and monocytes/macrophages in mouse peripheral blood.**

(A) 10 or 20 mg/kg STAT3-IN or STAT5-IN were injected into 6-week-old male mice intraperitoneally. 24 hours later, peripheral blood was collected, and the indicated proteins of nucleated cells were detected by using Western Blot.

(B) 20 mg/kg STAT3-IN or STAT5-IN were injected into 6-week-old male mice intraperitoneally every 2 days. After 7 days, peripheral blood and bone marrow cells were separated, and the levels of CD11b, Ly6G and F4/80 were detected by using flow cytometry.
