# Supplementary material for: Selective activation of STAT3 and STAT5 dictates the fate of myeloid progenitor cells
Source: Cell Death Discov. 2023 Jul 28;9:274. doi: 10.1038/s41420-023-01575-y (PMC10382539; doi:10.1038/s41420-023-01575-y)
Supplement: Supplementary file 2 — Key resources table [file 41420_2023_1575_MOESM2_ESM.docx]

**Key resources table**

| REAGENT or RESOURCE | SOURCE | IDENTIFIER |
| --- | --- | --- |
| Antibodies | | |
| Rabbit monoclonal anti-STAT3 | Cell Signaling Technology | Cat: #12640  RRID: AB_2629499 |
| Rabbit monoclonal anti-STAT5 | Cell Signaling Technology | Cat: #94205  RRID: AB_2737403 |
| Rabbit monoclonal anti-Phospho-STAT3 (Tyr705) | Cell Signaling Technology | Cat: #9145  RRID: AB_2491009 |
| Rabbit monoclonal Phospho-STAT5 (Tyr694) | Cell Signaling Technology | Cat: #4322  RRID: AB_10544692 |
| Mouse monoclonal anti-β-ACTIN | Cell Signaling Technology | Cat: #3700  RRID: AB_2242334 |
| PE anti-mouse/human CD11b antibody | Biolegend | Cat: #101208  RRID: AB_312791 |
| FITC anti-mouse Ly-6G Antibody | Biolegend | Cat: #127606  RRID: AB_1236494 |
| APC anti-mouse F4/80 Recombinant Antibody | Biolegend | Cat: #157306  RRID: AB_2832549 |
| PE rat IgG2b, κ isotype ctrl antibody | Biolegend | Cat: #400636  RRID: AB_893669 |
| FITC rat IgG2a, κ isotype ctrl antibody | Biolegend | Cat: #400506  RRID: AB_2736919 |
| APC mouse IgG1, κ isotype ctrl antibody | Biolegend | Cat: #400119  RRID: AB_2888687 |
|  |  |  |
| Chemicals, peptides, and recombinant proteins | | |
| STAT3-IN-1 | MedChem Express | HY-100753; CAS: 2059952-75-5 |
| STAT5-IN-2 | MedChem Express | HY-102048; CAS: 2111834-61-6 |
| Recombinant Murine SCF | Pepro Tech | Cat: 250-03 |
| Recombinant Murine G-CSF | Pepro Tech | Cat: 250-05 |
| Recombinant Murine GM-CSF | Pepro Tech | Cat: 315-03 |
| Recombinant Murine IL3 | Pepro Tech | Cat: 213-13 |
| Recombinant Murine IL6 | Pepro Tech | Cat: 216-16 |
| Recombinant Murine puromycin | Shanghai Yeasen Biotechnology | Cat: #60210ES25 |
| polybrene | Shanghai Yeasen Biotechnology | Cat: #40804ES76 |
|  |  |  |
| Experimental models: Cell lines | | |
| mEB8-ER | In house | N/A |
|  |  |  |
| Experimental models: Organisms/strains | | |
| Mouse: C57BL/6 | Shanghai JieSiJie Laboratory Animal Co., Ltd. | MGI:2159769 |
|  |  |  |
| Oligonucleotides | | |
| *actb*_Fw: GGCTGTATTCCCCTCCATCG | Azenta Life Sciences | N/A |
| *actb*_Rev: CCAGTTGGTAACAATGCCATGT | Azenta Life Sciences | N/A |
| socs3_Fw: CGTGCGCCATGGTCACCC | Azenta Life Sciences | N/A |
| socs3_Rev: GCCTCGGAGGAGAGGCGA | Azenta Life Sciences | N/A |
|  |  |  |
| Recombinant DNA | | |
| pSIREN-RetroQ | Clontech | Addgene_ 631526 |
| p-SIREN-stat3-sh1 | This paper | N/A |
| p-SIREN-stat3-sh2 | This paper | N/A |
| p-SIREN-stat5-sh1 | This paper | N/A |
| p-SIREN-stat5-sh2 | This paper | N/A |
| p-SIREN-socs3-sh | This paper | N/A |
| pBABE-puro | Public Protein/Plasmid Library | Addgene_1764 |
| pBABE-stat3-flag | This paper | N/A |
| pBABE-stat5a | This paper | N/A |
| pBABE-stat5b | This paper | N/A |
| p-BABE-socs3-flag | This paper | N/A |
|  |  |  |
| Software and algorithms | | |
| Flowjo | FlowJo,LLC | www.flowjo.com |
| GraphPad Prism8 | Dotmatics | www.graphpad.com |
