# Supplementary material for: Selective activation of STAT3 and STAT5 dictates the fate of myeloid progenitor cells
Source: Cell Death Discov. 2023 Jul 28;9:274. doi: 10.1038/s41420-023-01575-y (PMC10382539; doi:10.1038/s41420-023-01575-y)

Figure 2

Figure 2A

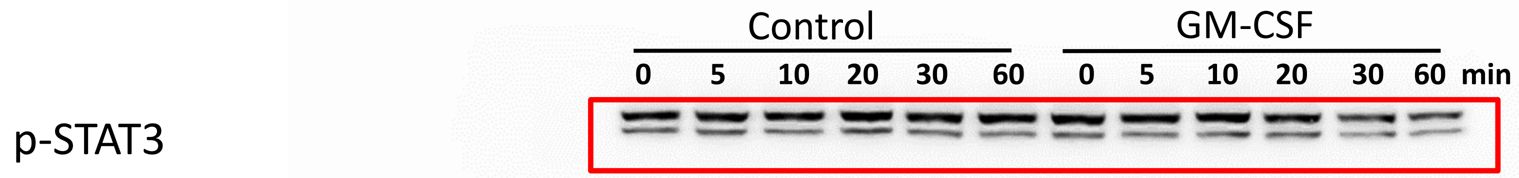

Figure 2A

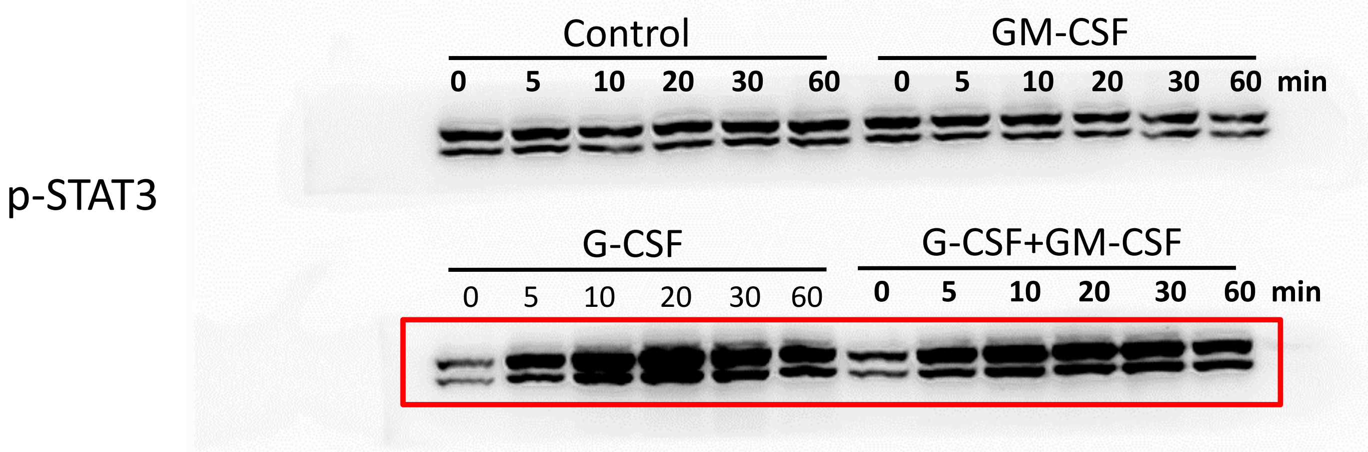
p-STAT3
Control
GM-CSF
0 5 10 20 30 60
0 5 10 20 30 60 min
G-CSF
G-CSF+GM-CSF
0 5 10 20 30 60
0 5 10 20 30 60 min

Figure 2A

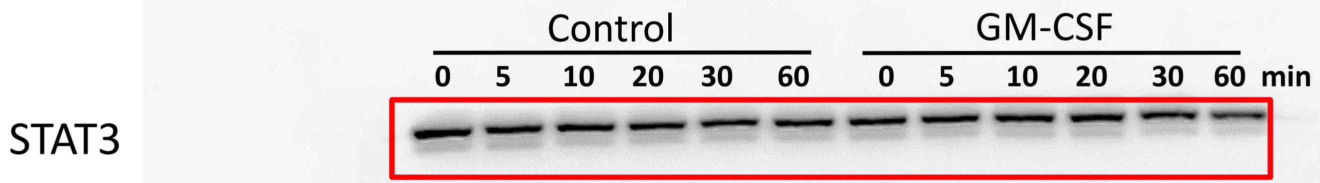

Figure 2A

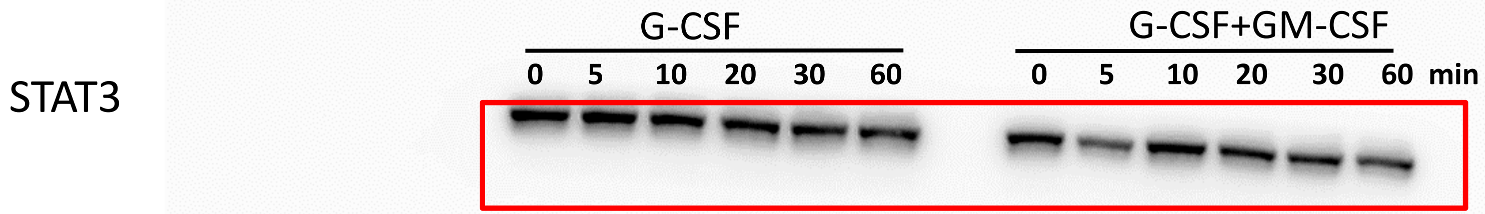

Figure 2A

p-STAT5

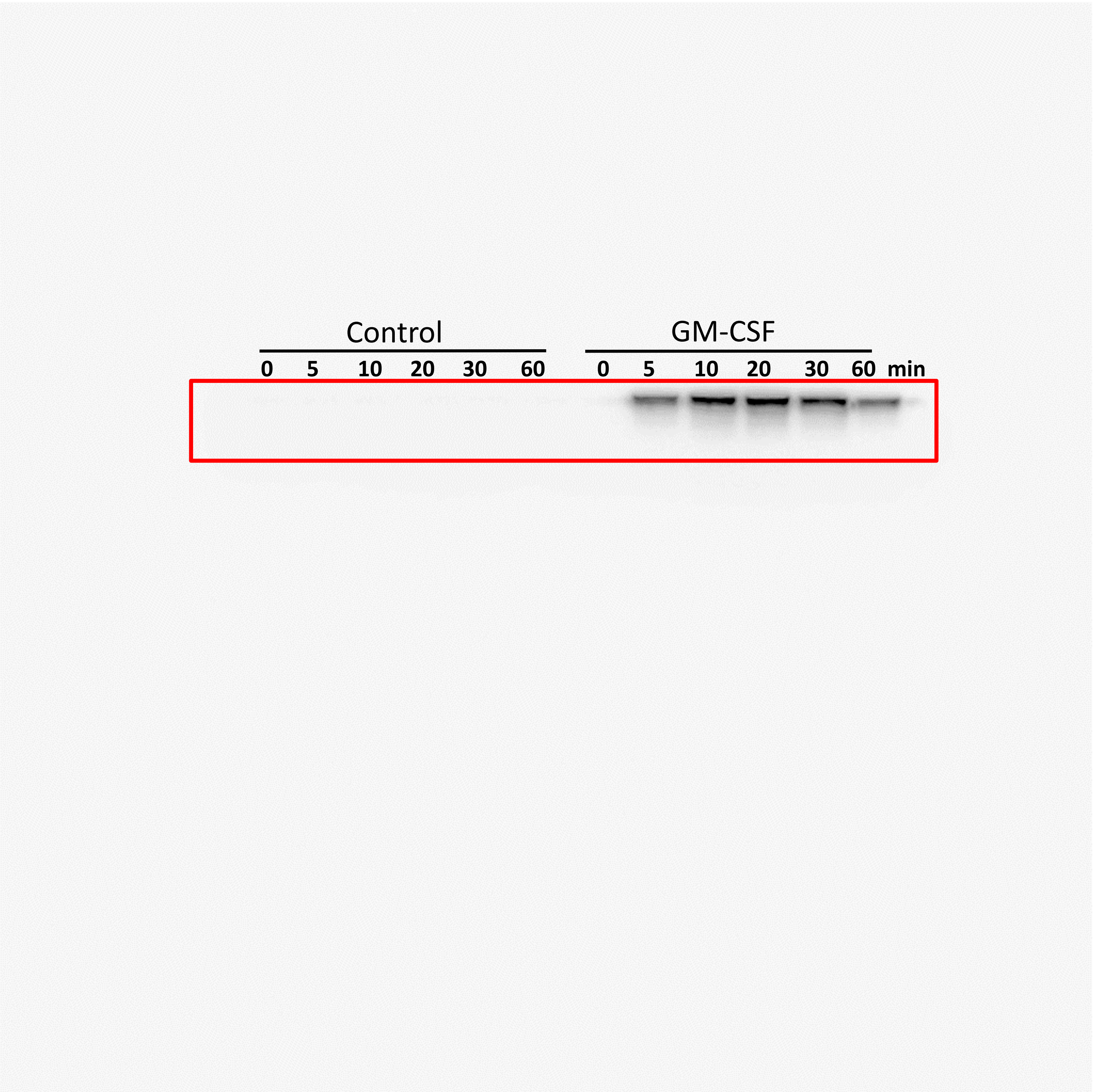

Figure 2A

p-STAT5

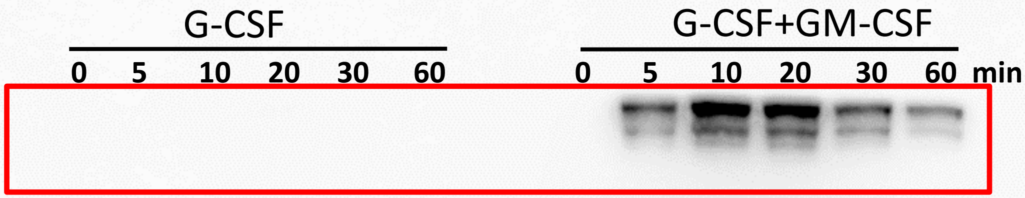

Figure 2A

p-STAT5

Long time exposure

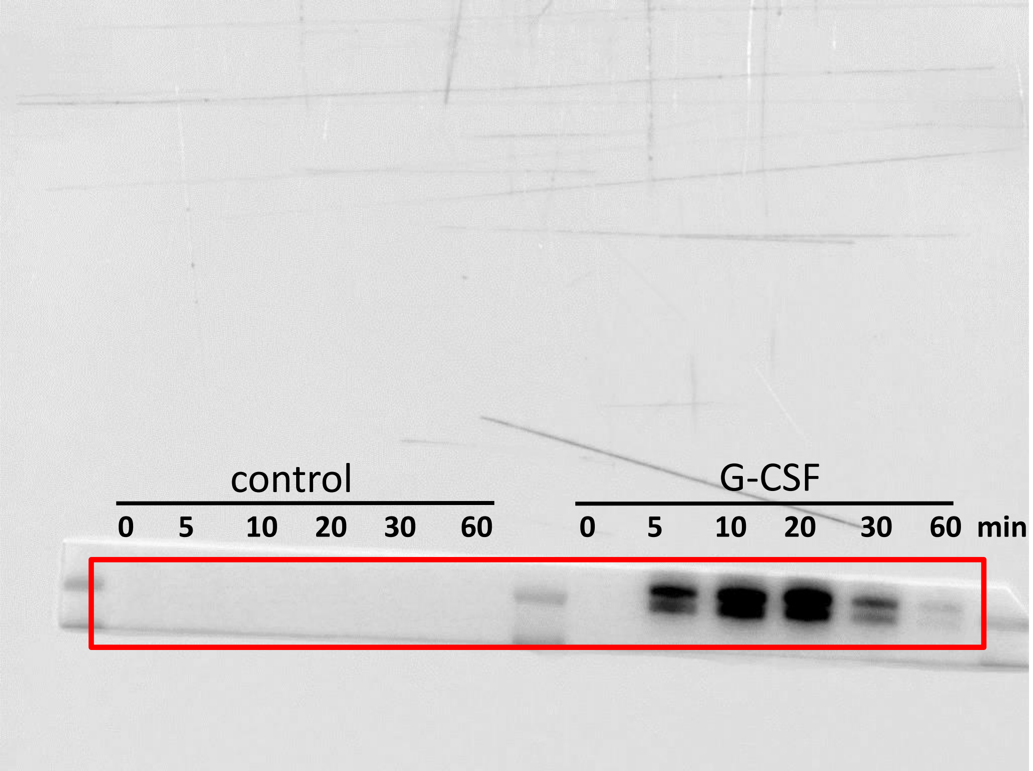

Figure 2A

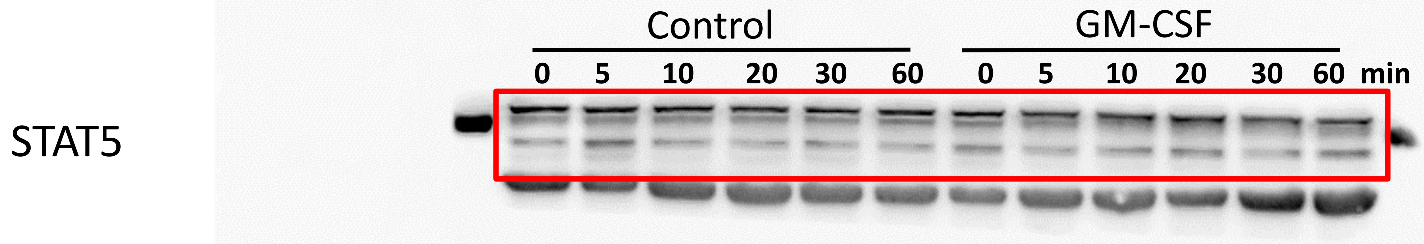

Figure 2A

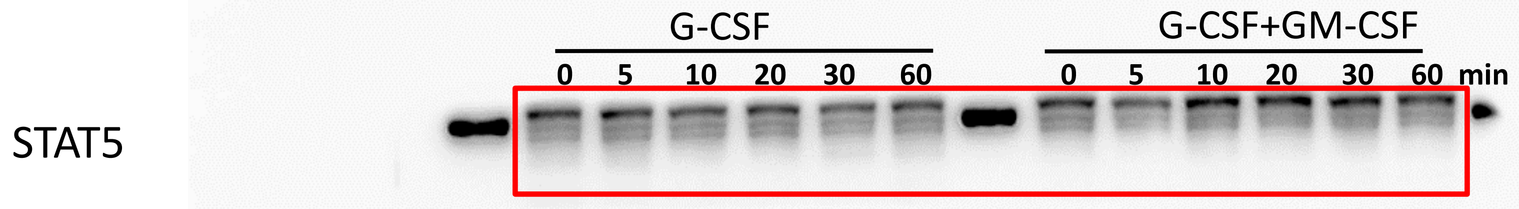

Figure 2A

ACTB

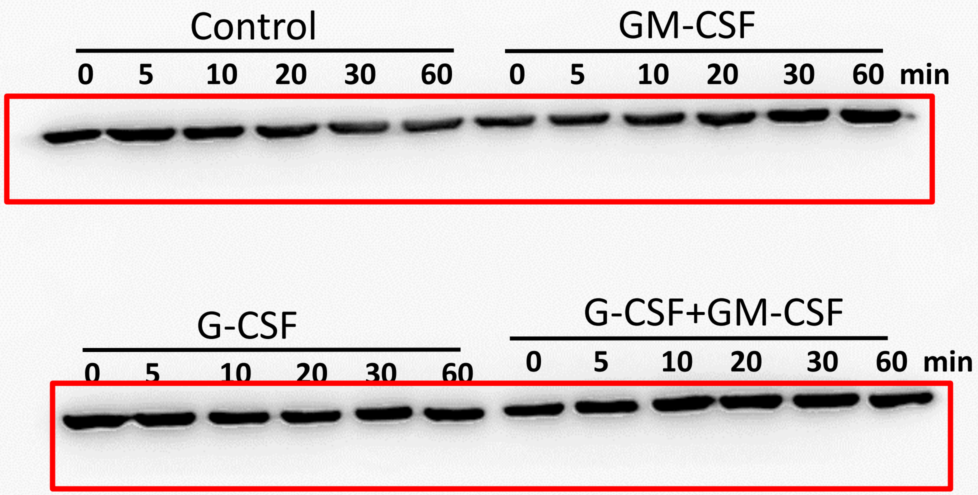

Figure 2B

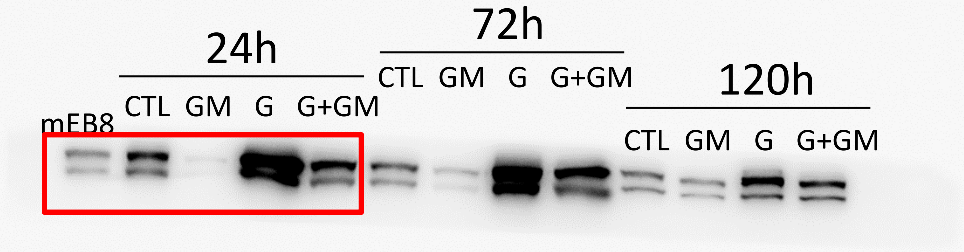

p-STAT3

Figure 2B

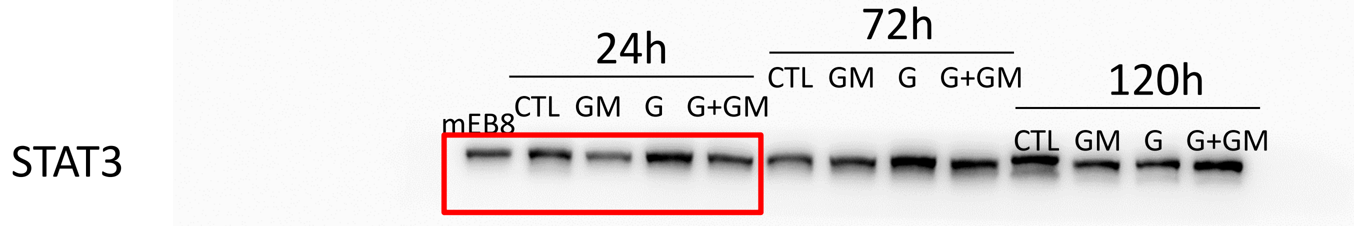

Figure 2B

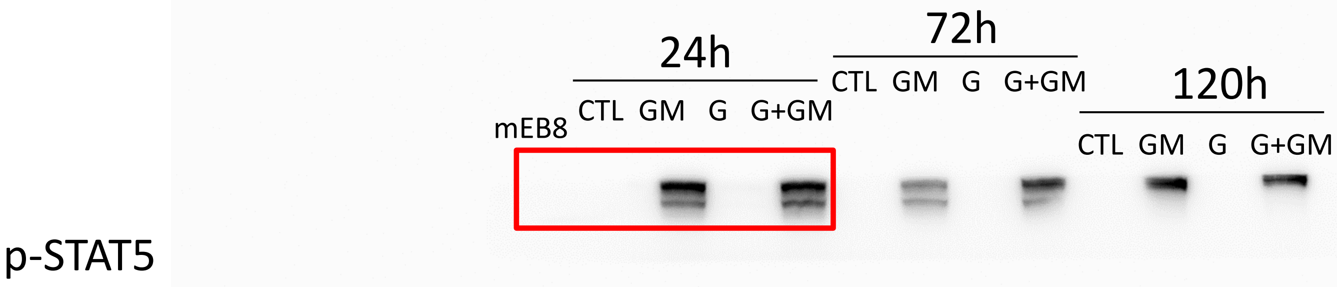

Figure 2B

STAT5

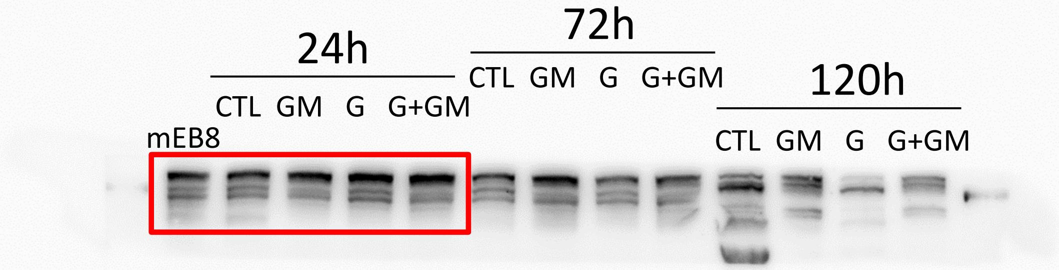

Figure 2B

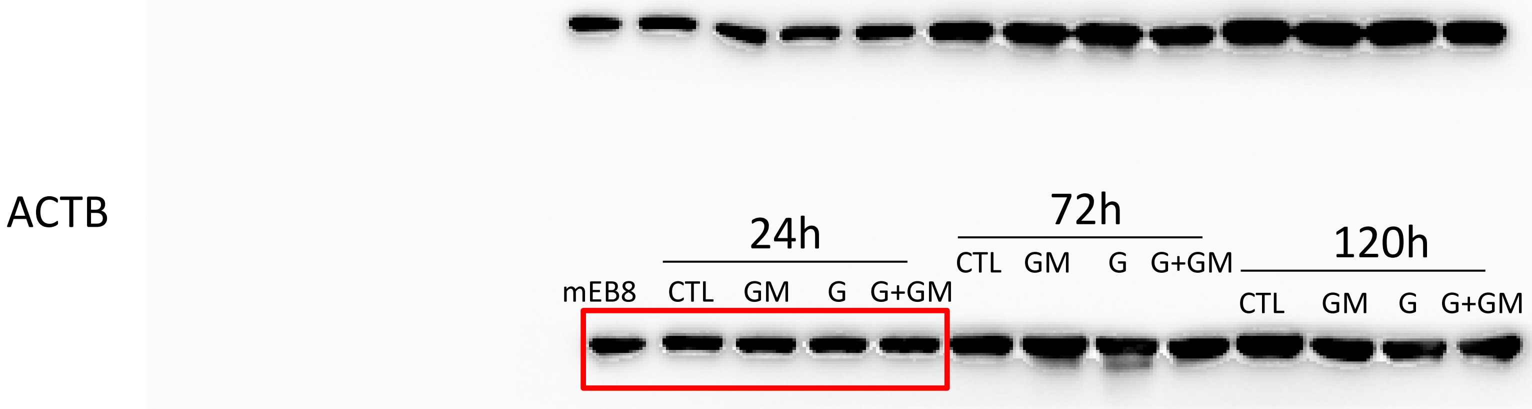

Figure 5

Figure 5A

p-STAT3

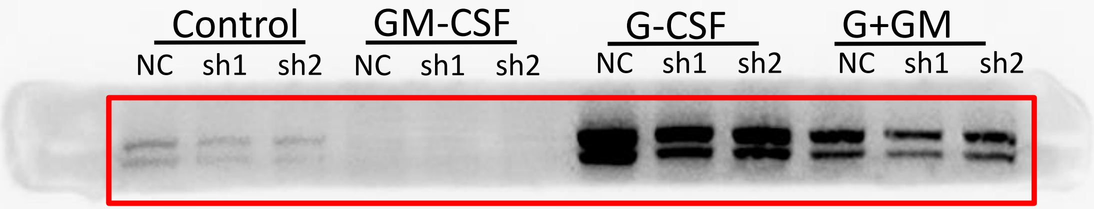

Figure 5A

STAT3

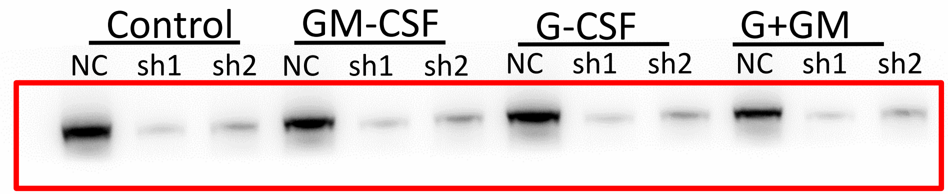

Figure 5A

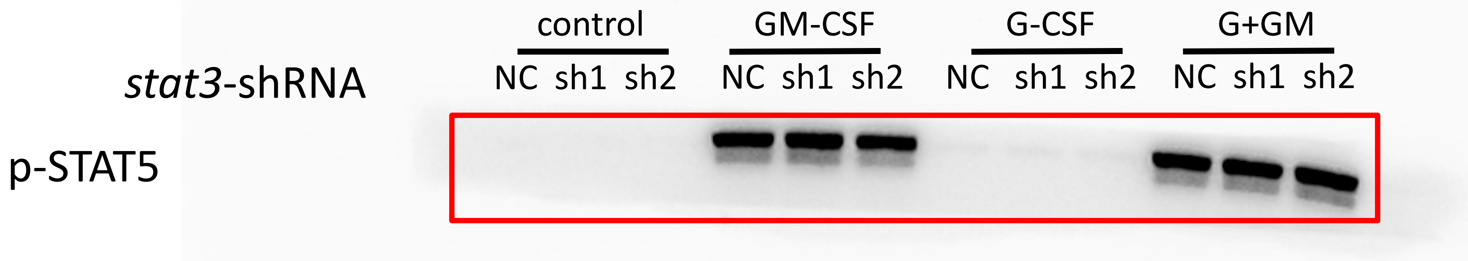
control
GM-CSF
G-CSF
G+GM
stat3-shRNA
NC sh1 sh2 NC sh1 sh2 NC sh1 sh2 NC sh1 sh2
p-STAT5

Figure 5A

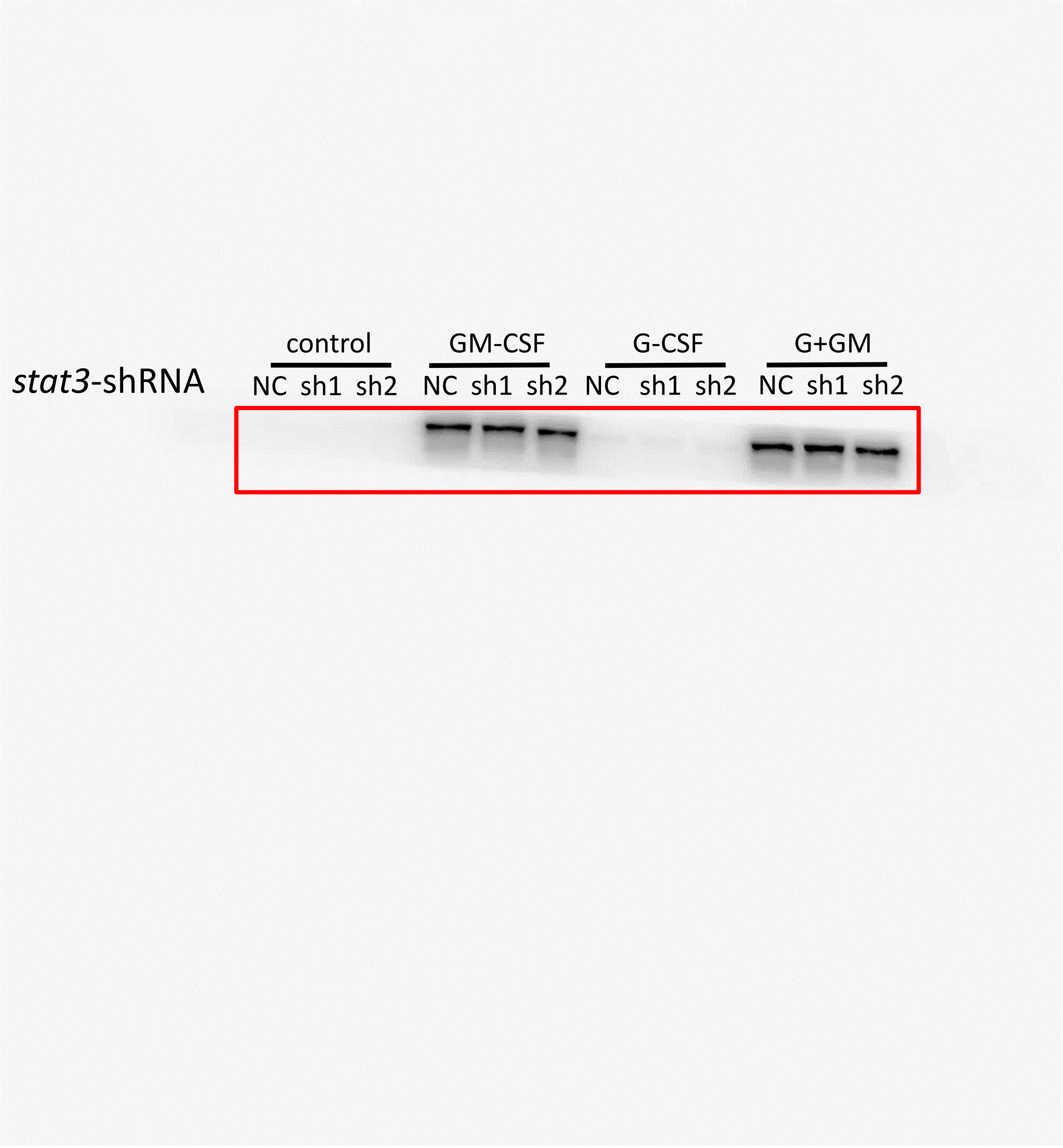
p-STAT5
stat3-shRNA
control
GM-CSF
G-CSF
G+GM
NC sh1 sh2 NC sh1 sh2 NC sh1 sh2 NC sh1 sh2

Figure 5A

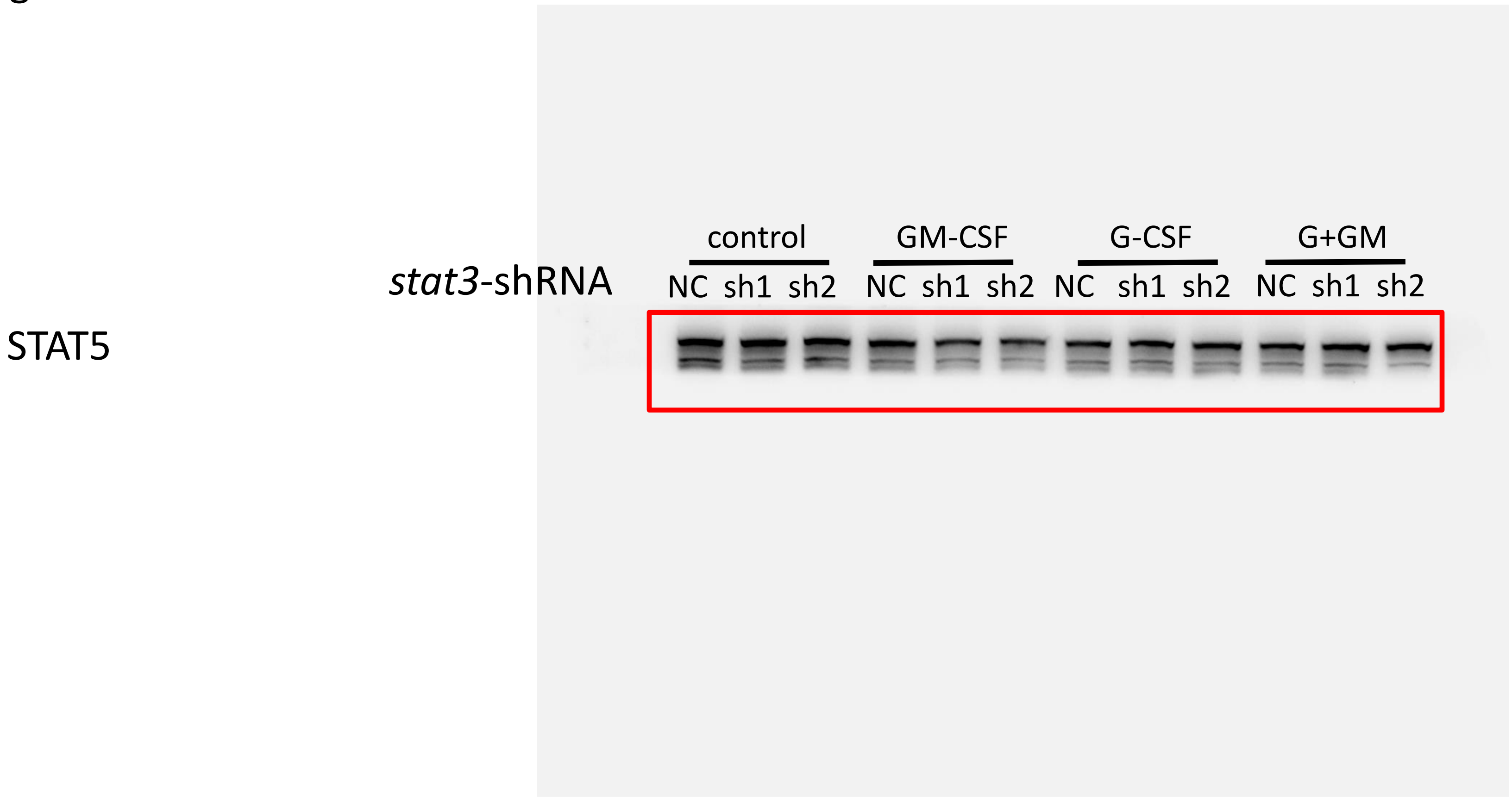
control
GM-CSF
G-CSF
G+GM
stat3-shRNA
NC sh1 sh2 NC sh1 sh2 NC sh1 sh2 NC sh1 sh2
STAT5

Figure 5A

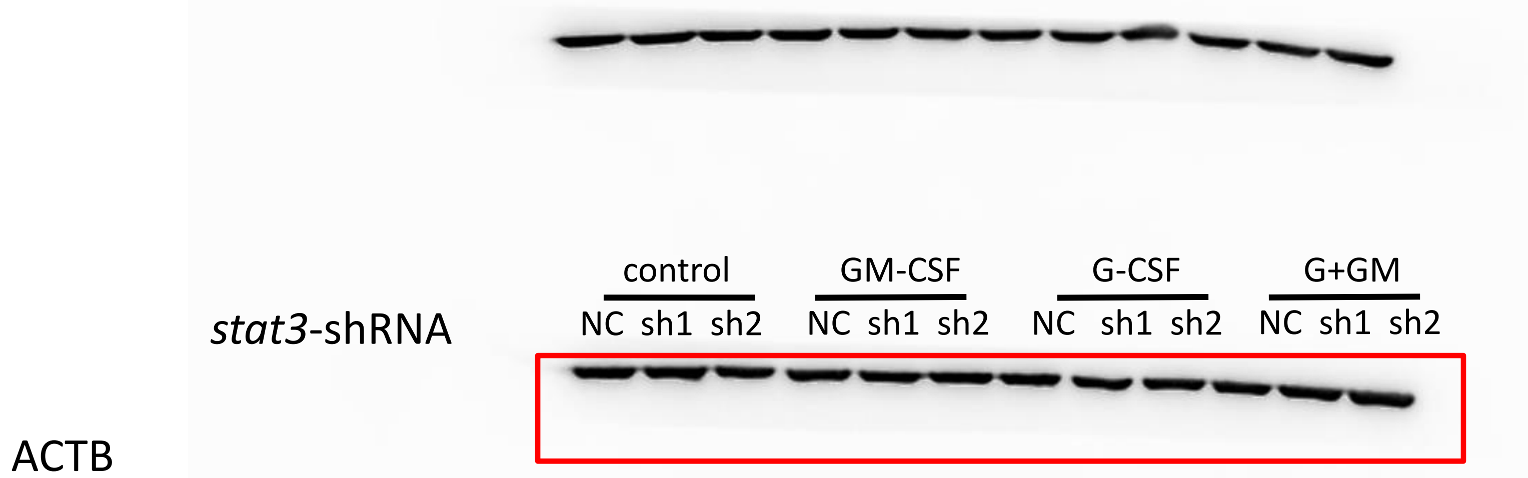
control
GM-CSF
G-CSF
G+GM
stat3-shRNA
NC sh1 sh2
NC sh1 sh2
NC sh1 sh2
NC sh1 sh2
ACTB

Figure 5B

p-STAT3

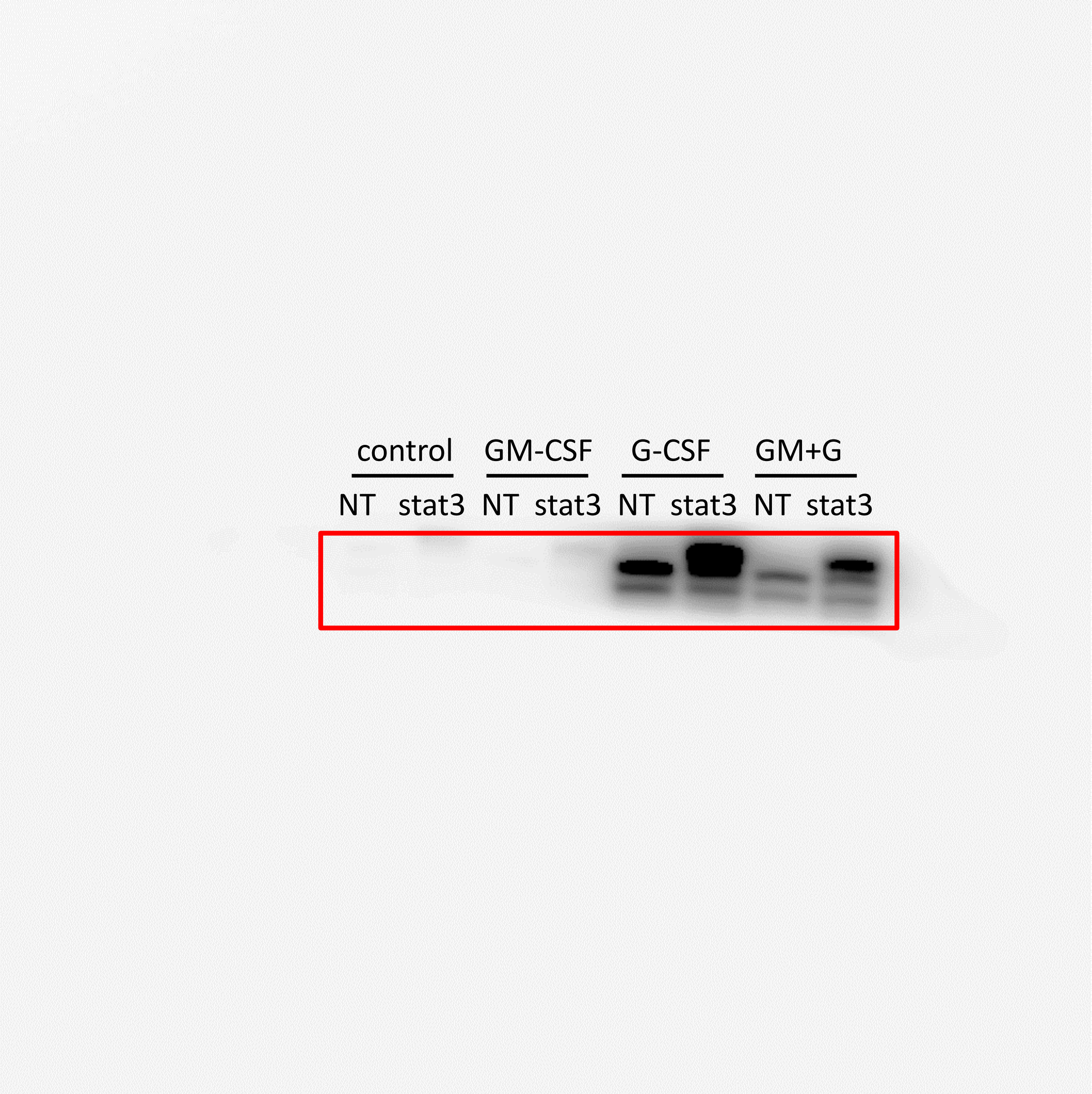

Figure 5B

STATR3

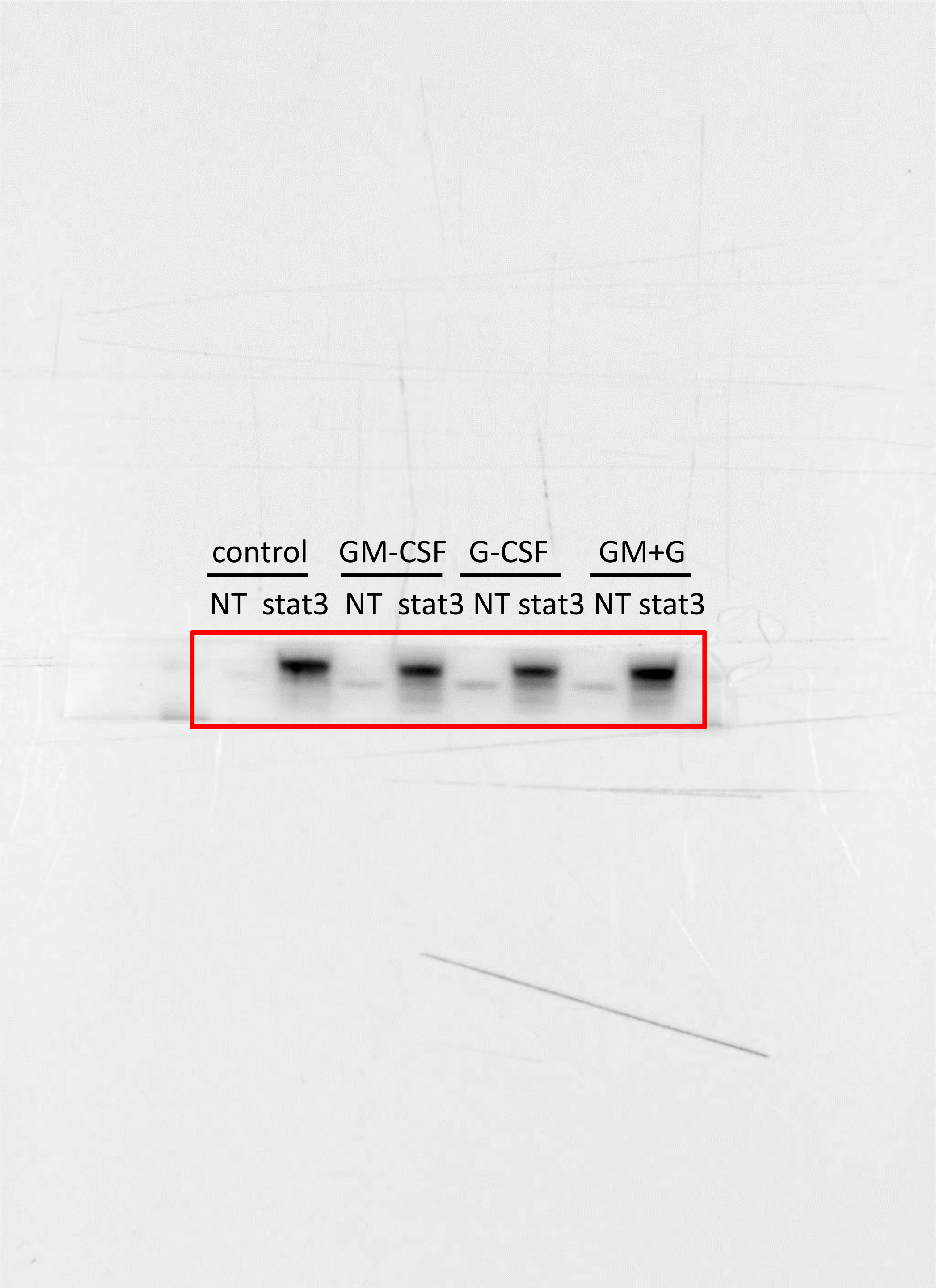

Figure 5B

p-STAT5

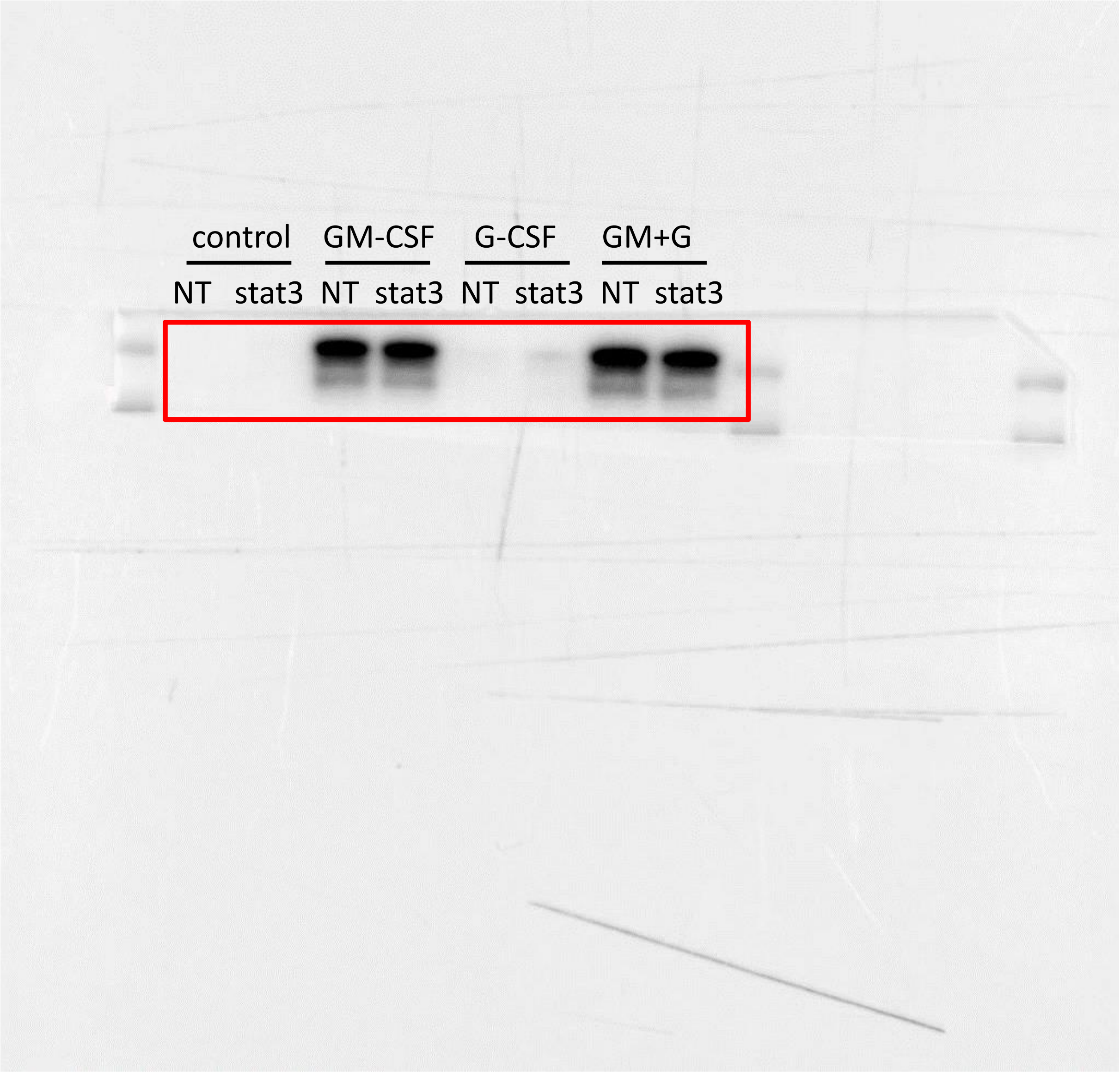

Figure 5B

STAT5

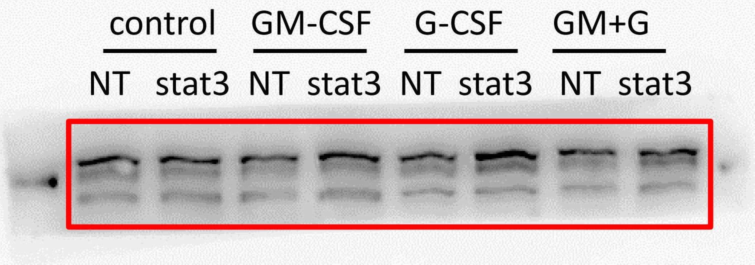

Figure 5B

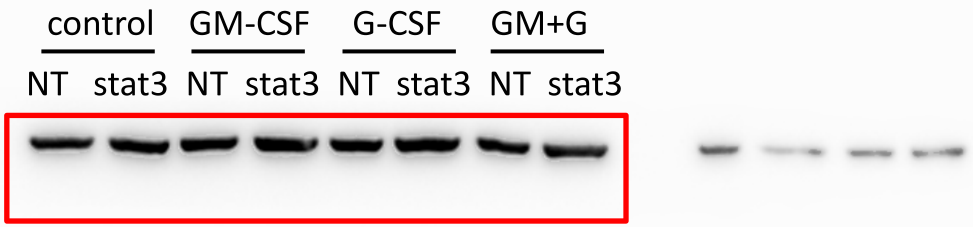

ACTB

Figure 5C

p-STAT5

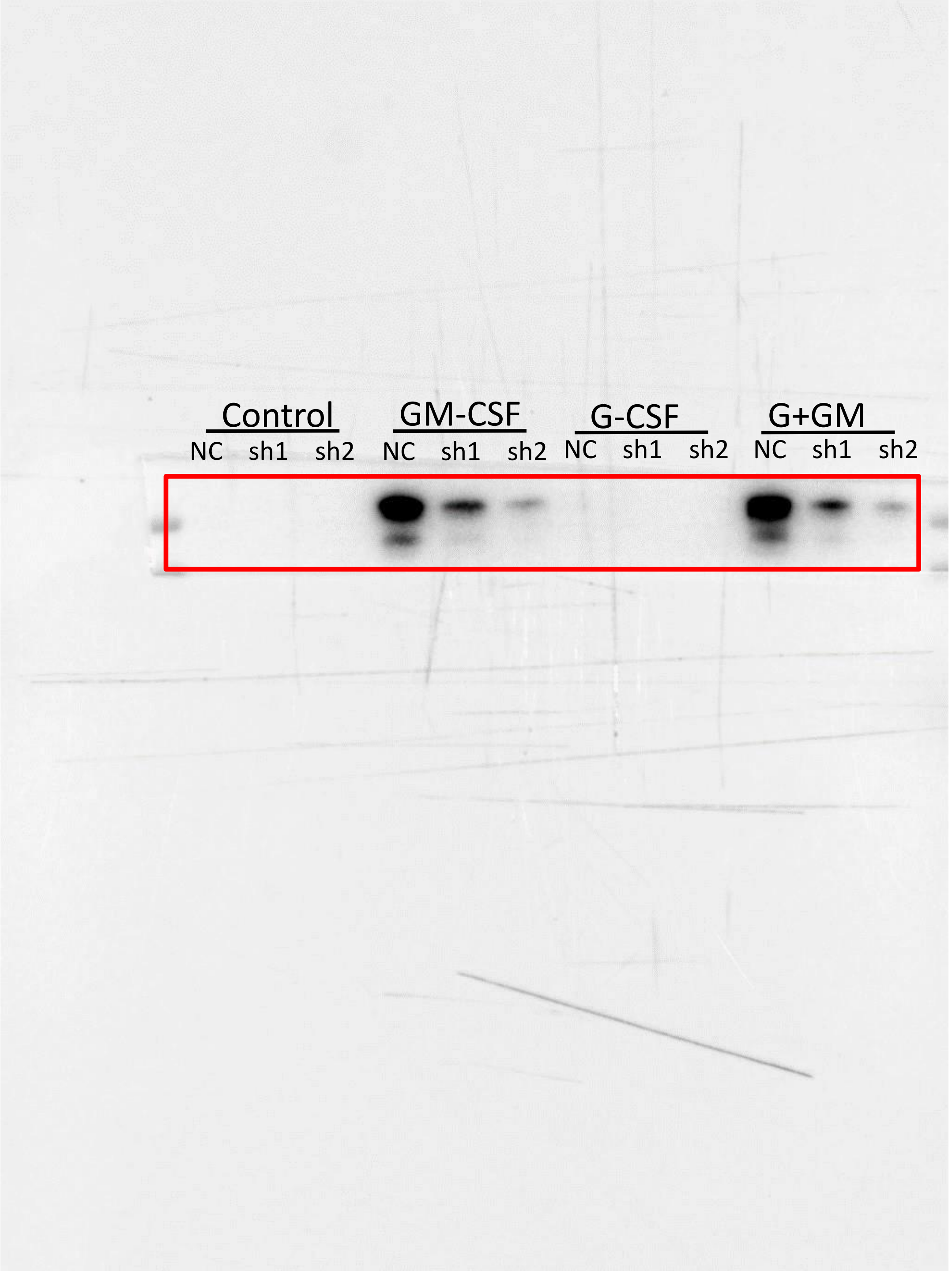

Figure 5C

STAT5

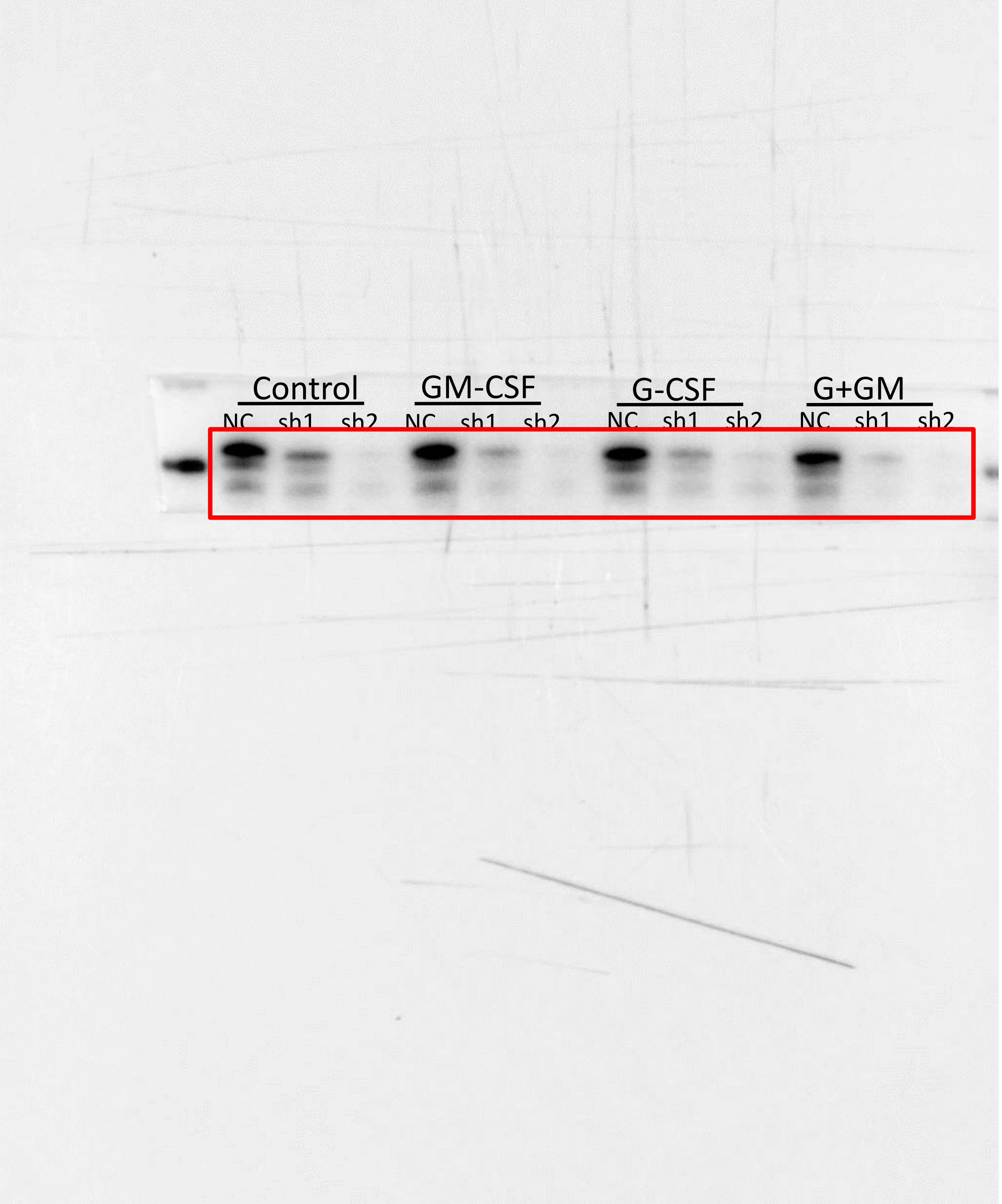

Figure 5C

p-STAT3

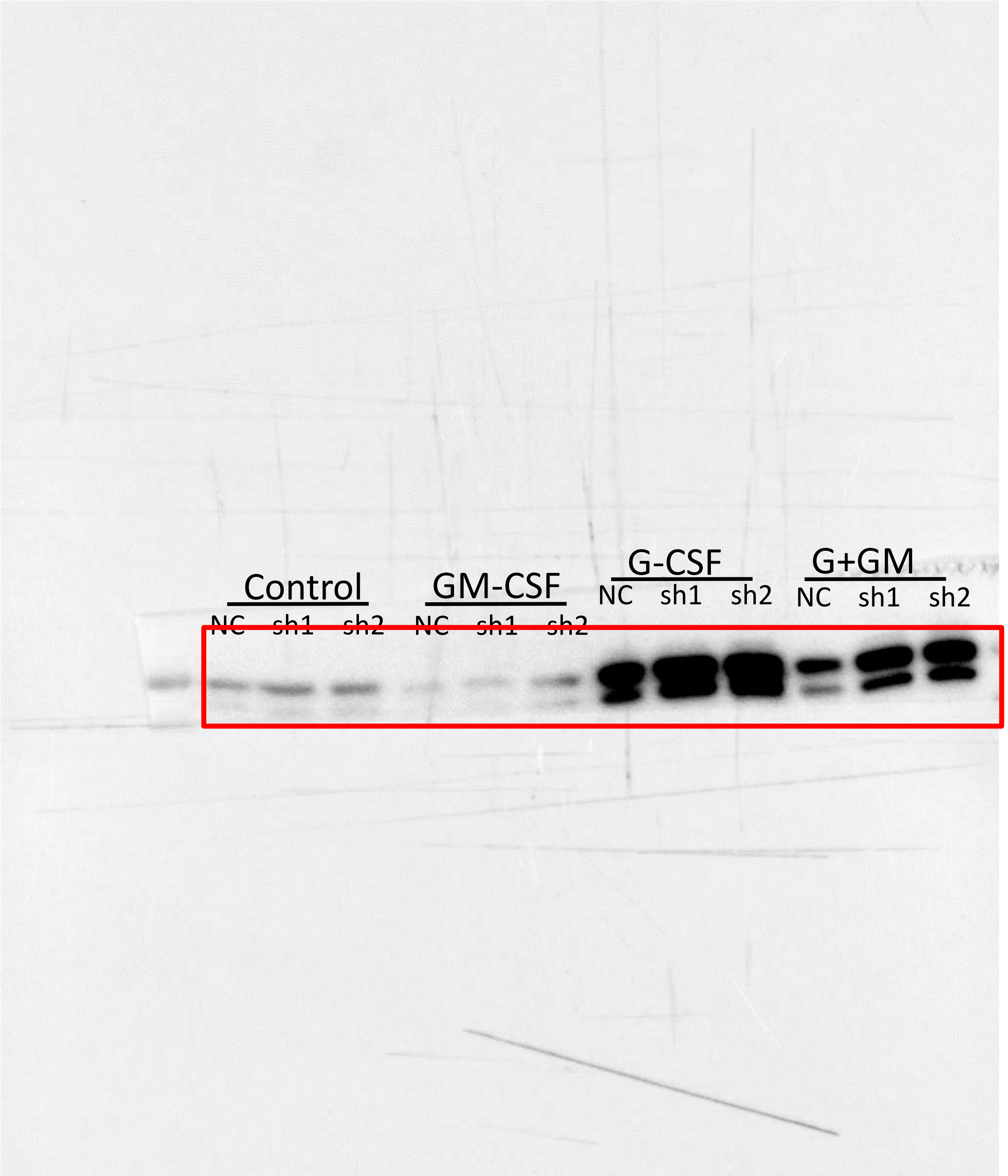

Figure 5C

STAT3

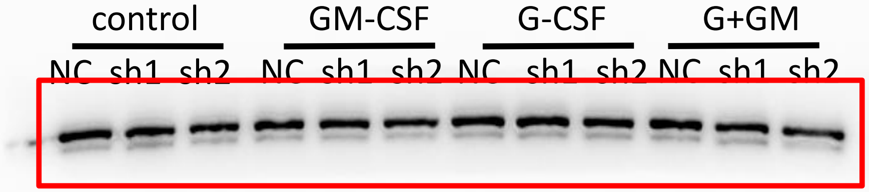

Figure 5C

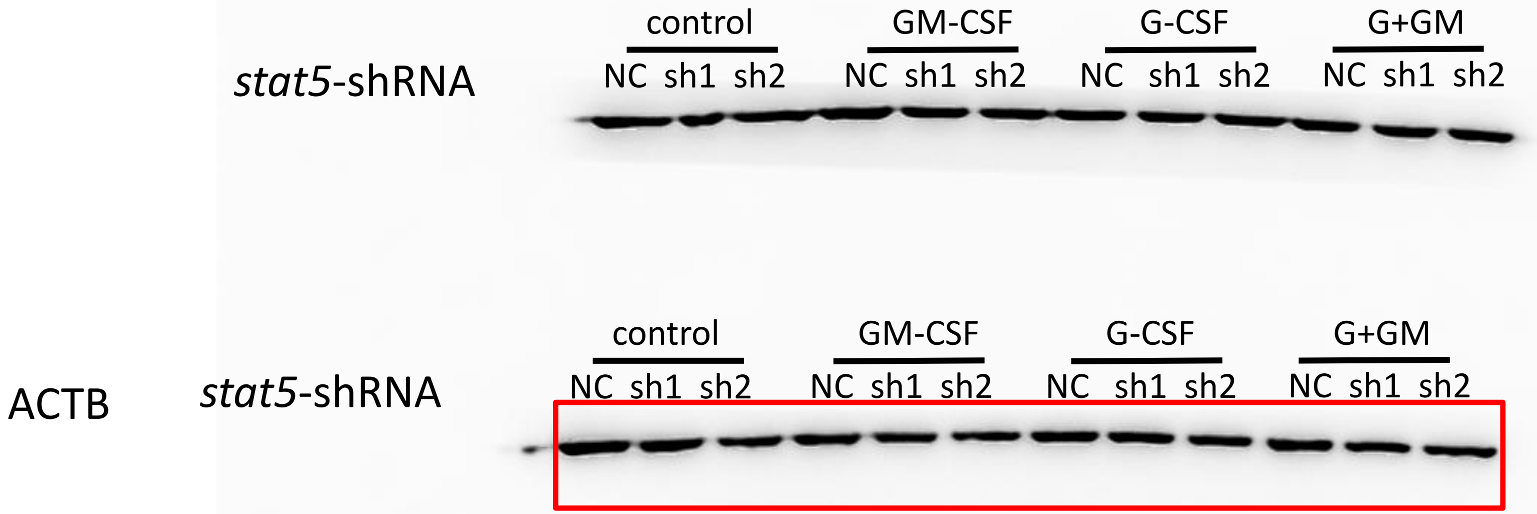
control
GM-CSF
G-CSF
G+GM
stat5-shRNA
NC sh1 sh2 NC sh1 sh2 NC sh1 sh2 NC sh1 sh2
ACTB
control
GM-CSF
G-CSF
G+GM
stat5-shRNA
NC sh1 sh2 NC sh1 sh2 NC sh1 sh2 NC sh1 sh2

Figure 5D

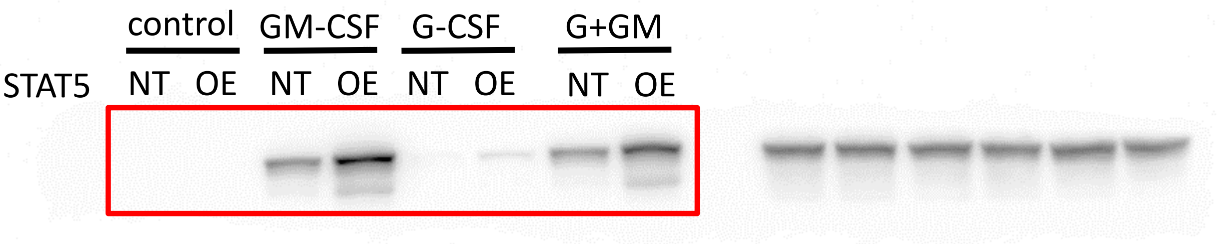

Figure 5D

STAT5

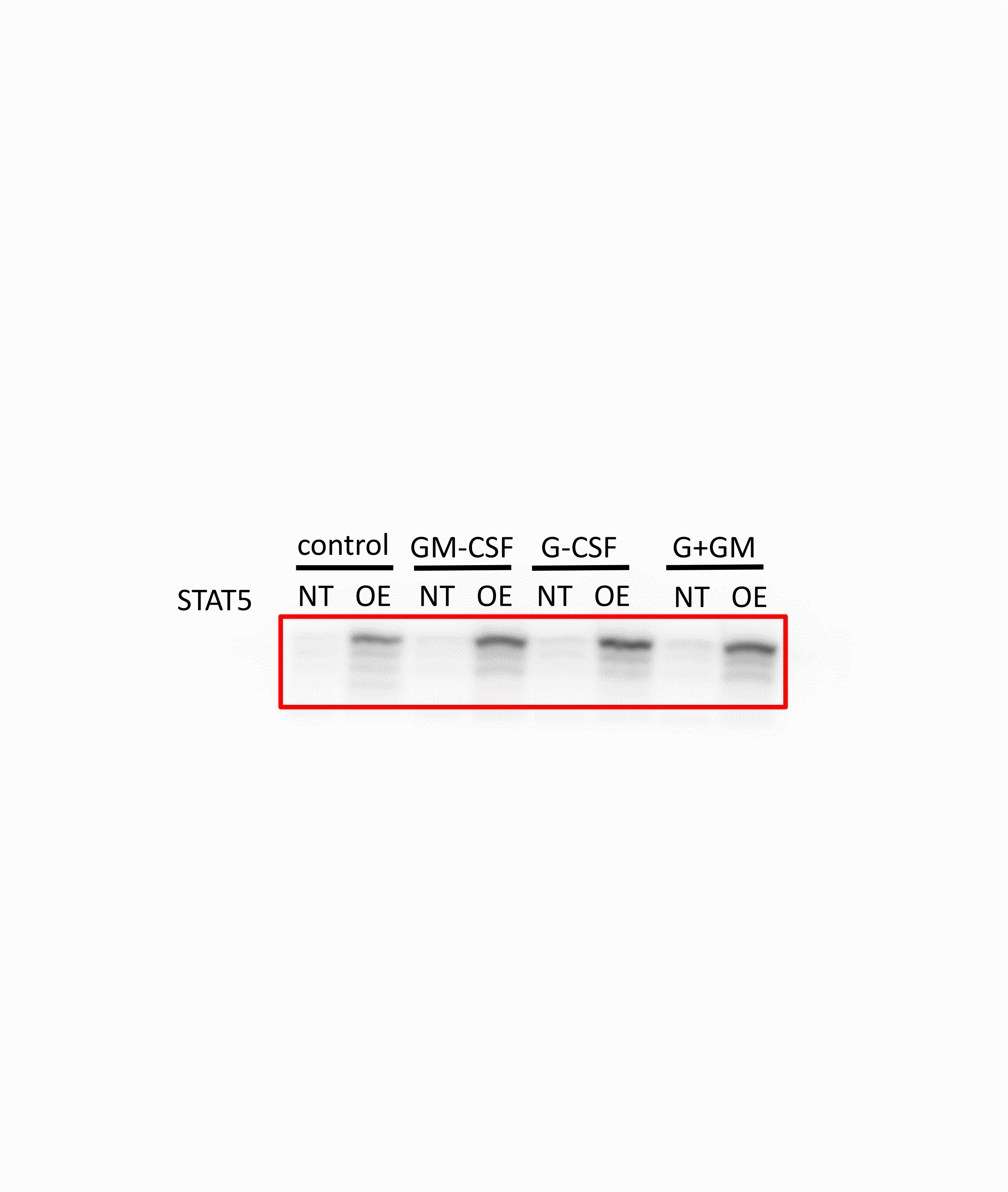

Figure 5D

p-STAT3

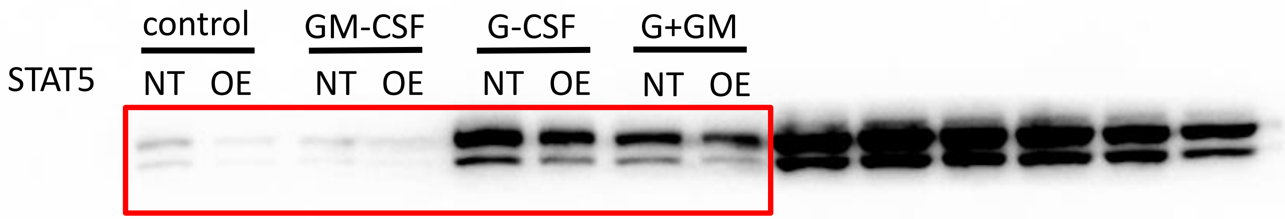

Figure 5D

STAT3

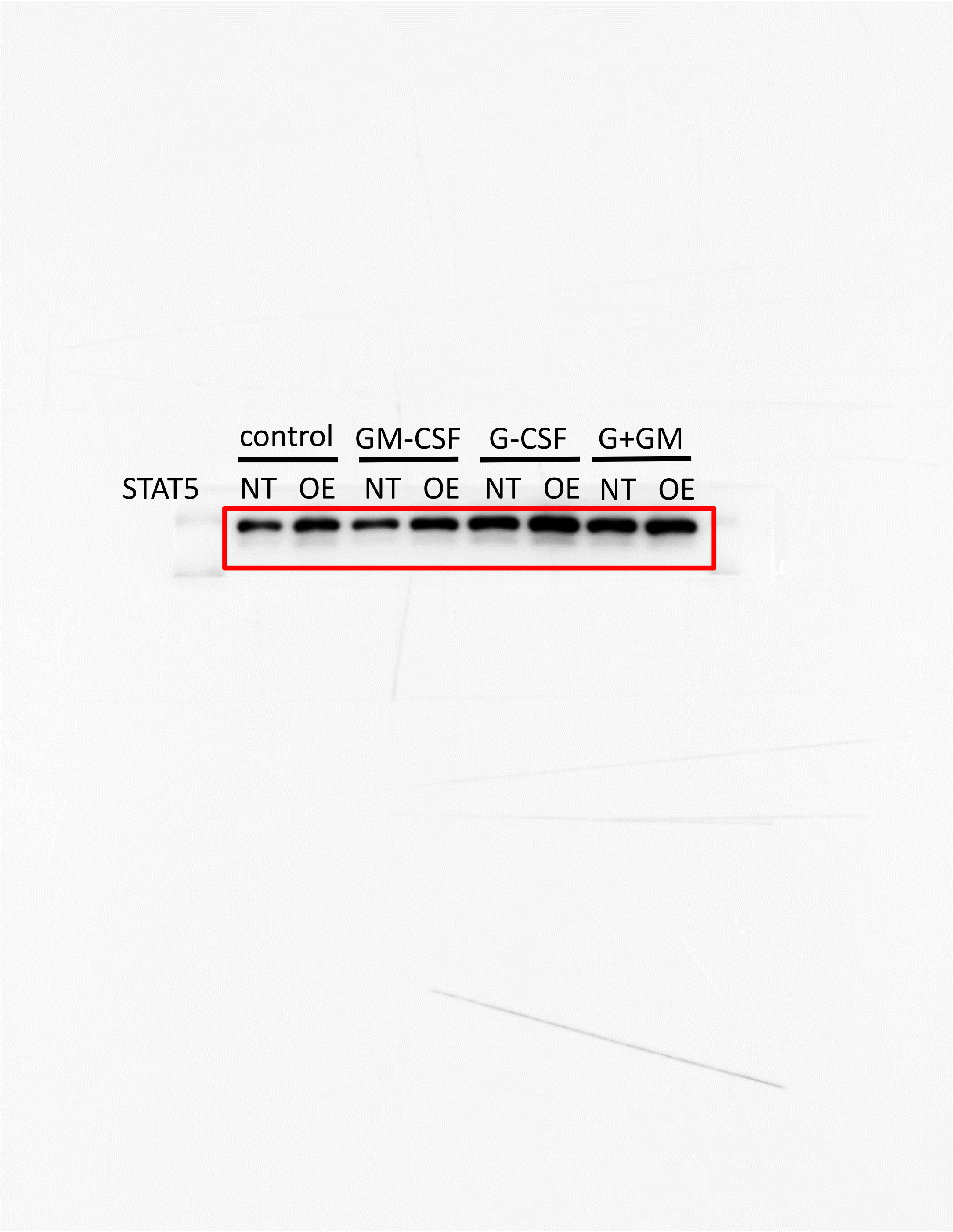

Figure 5D

ACTB

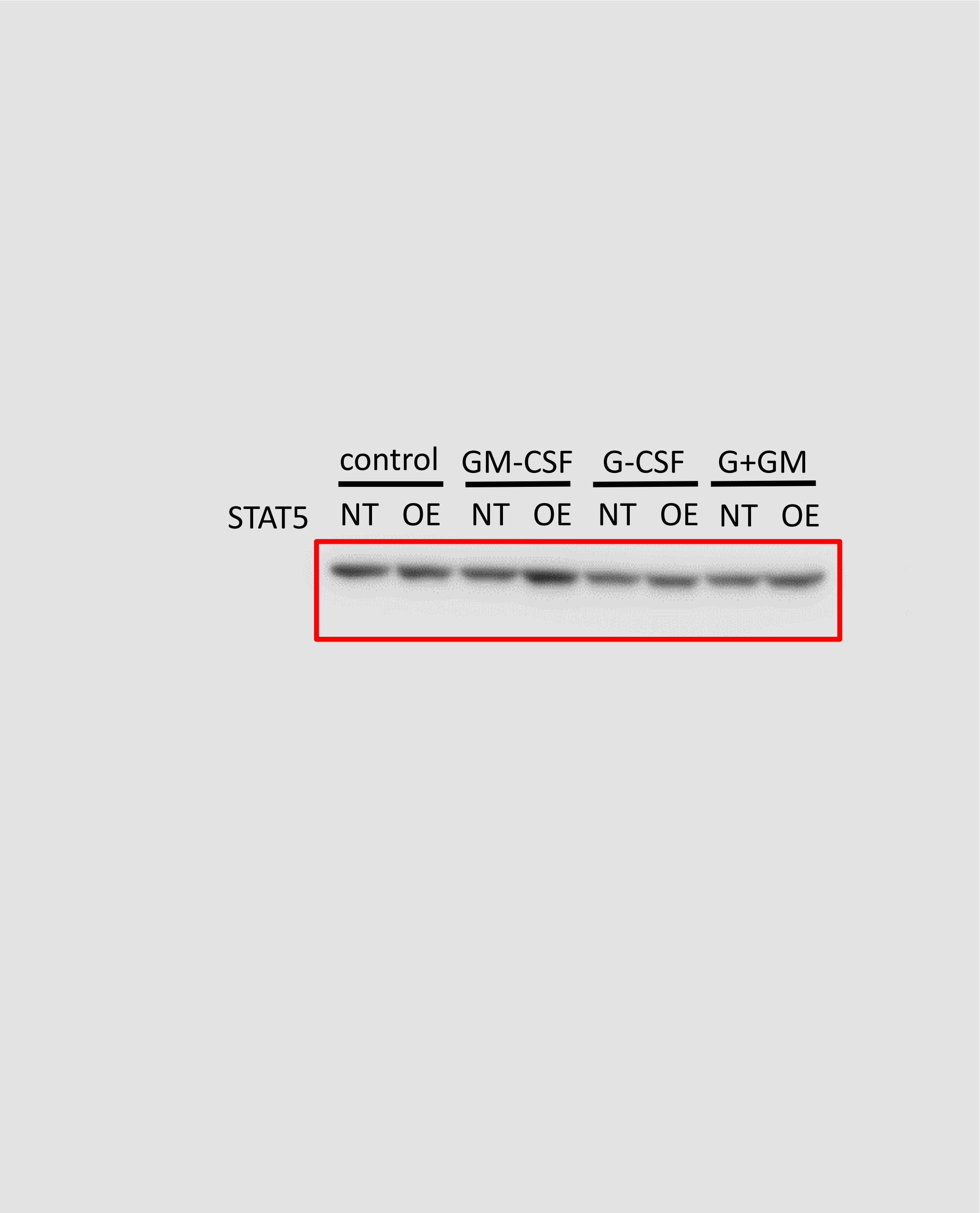

Figure 5E

p-STAT3

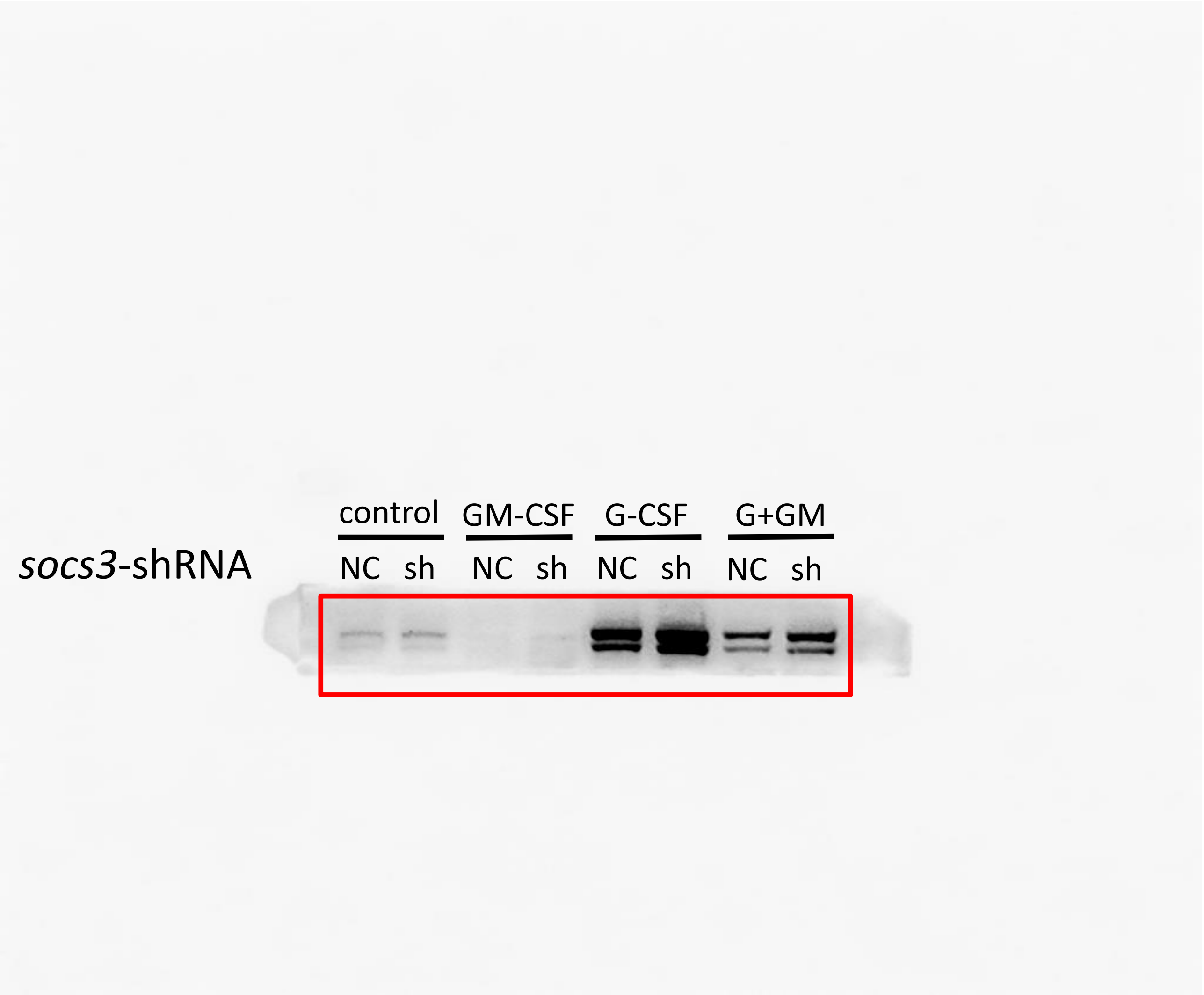

Figure 5E

STAT3

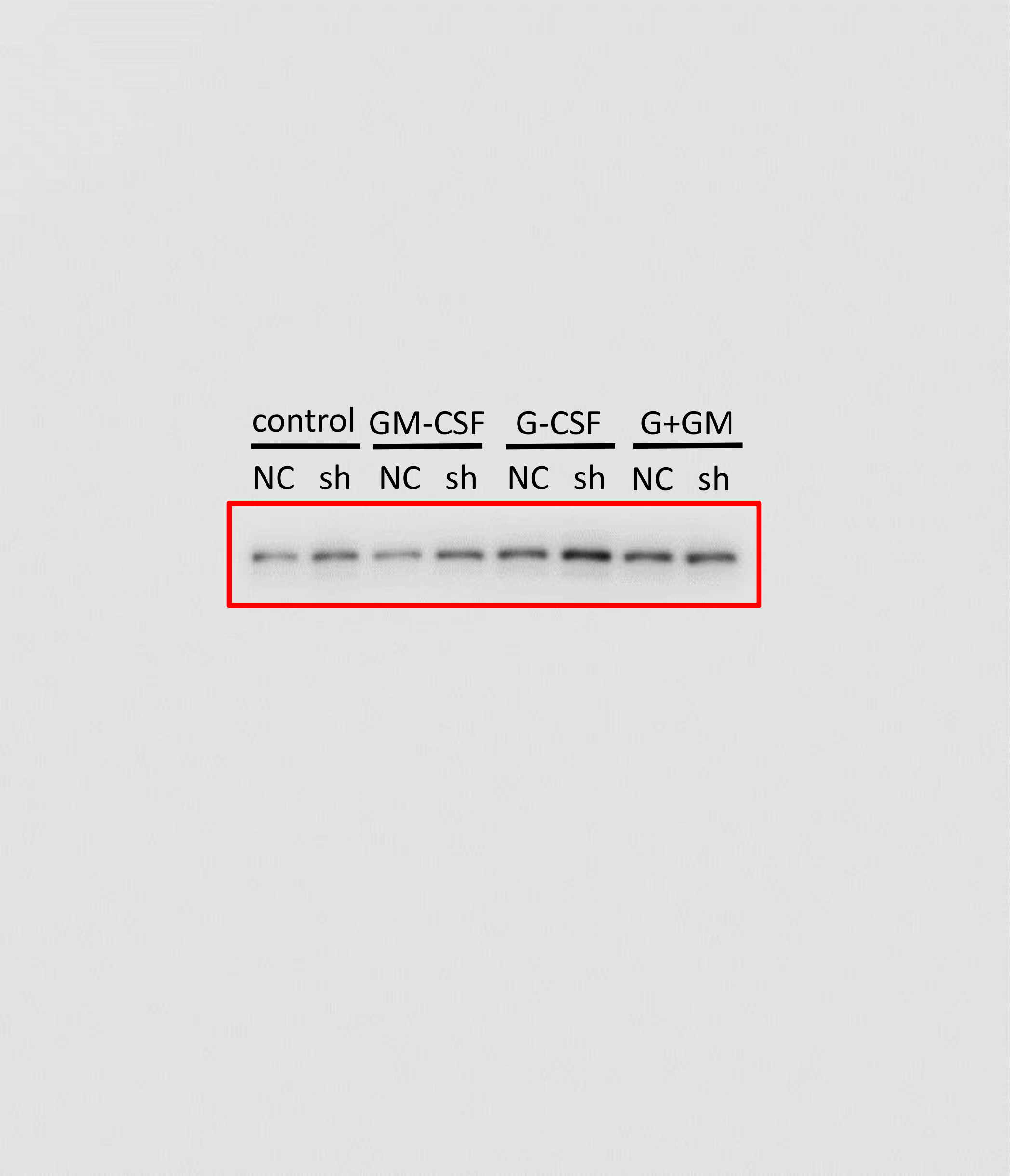

Figure 5E

P-STAT5

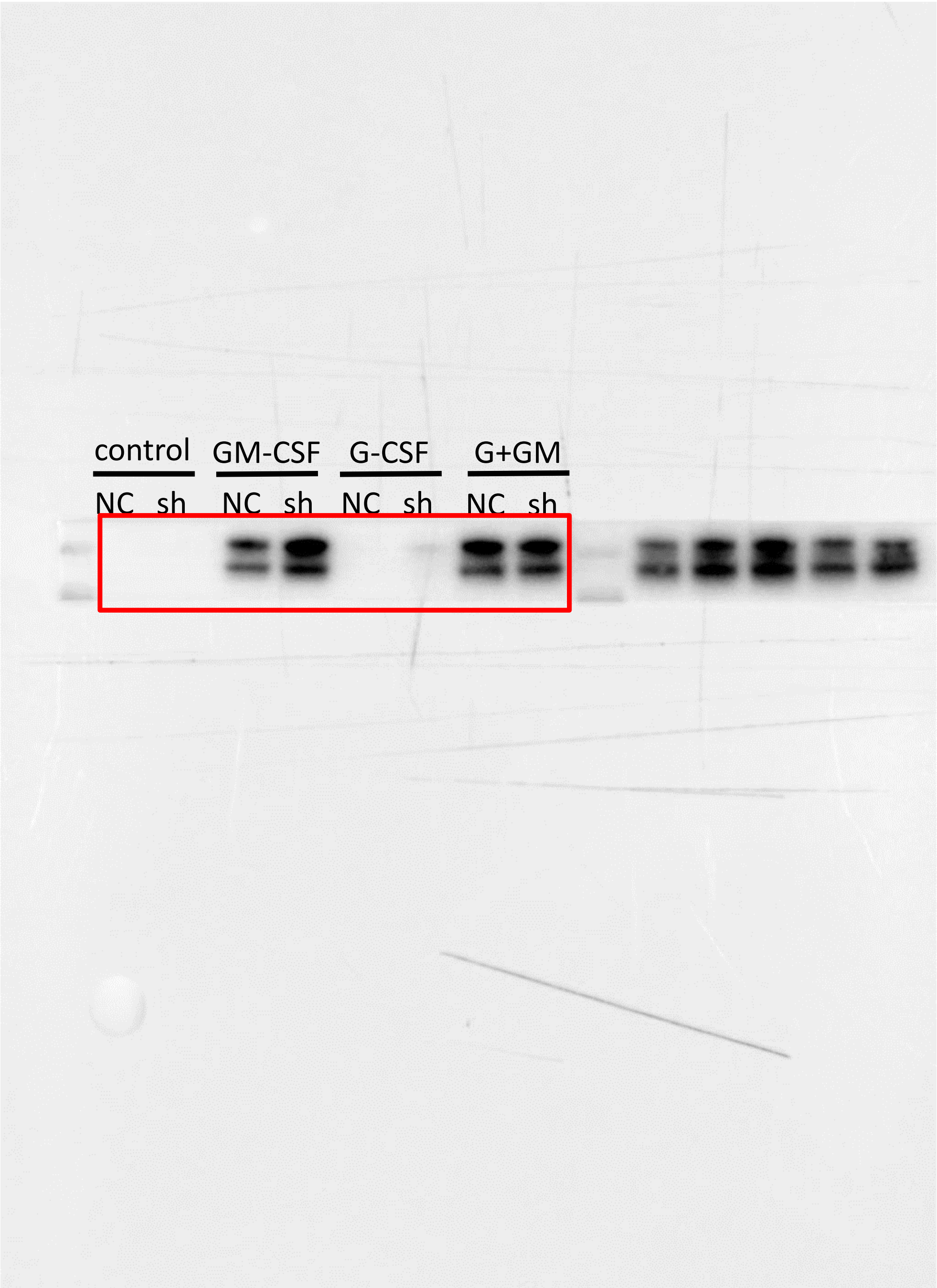

Figure 5E

STAT5

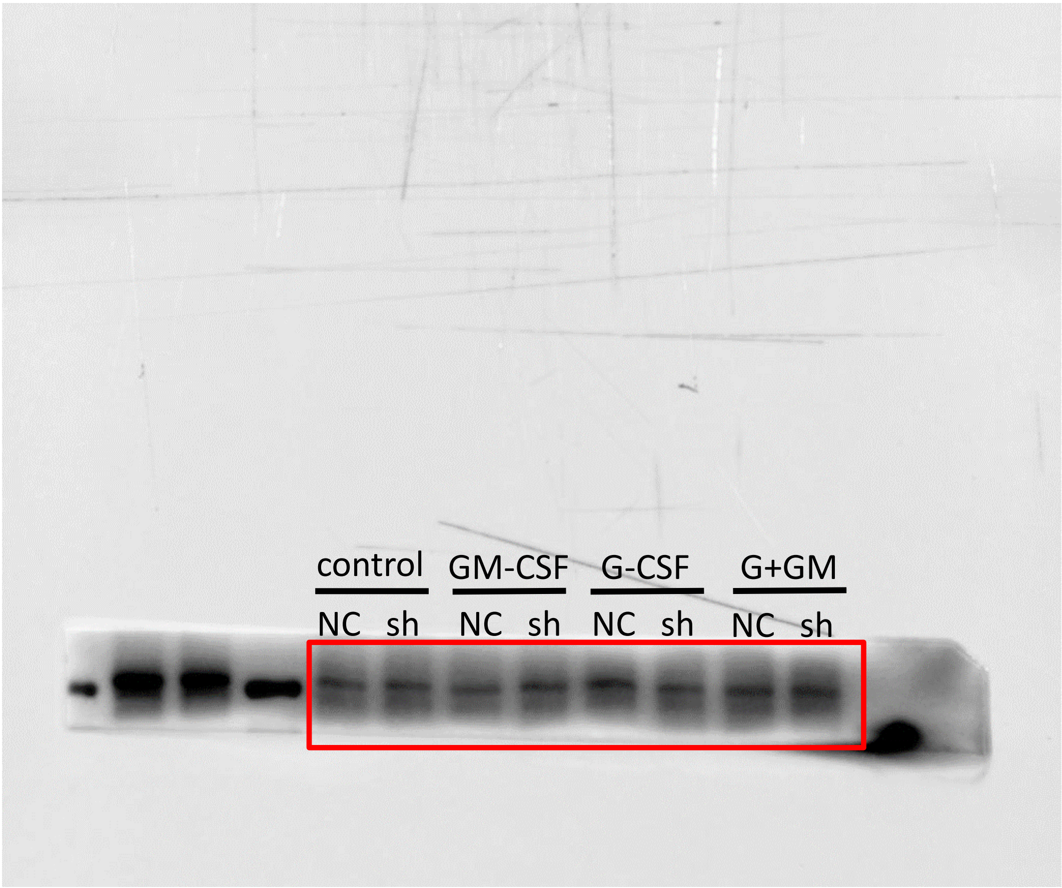

Figure 5E

ACTB

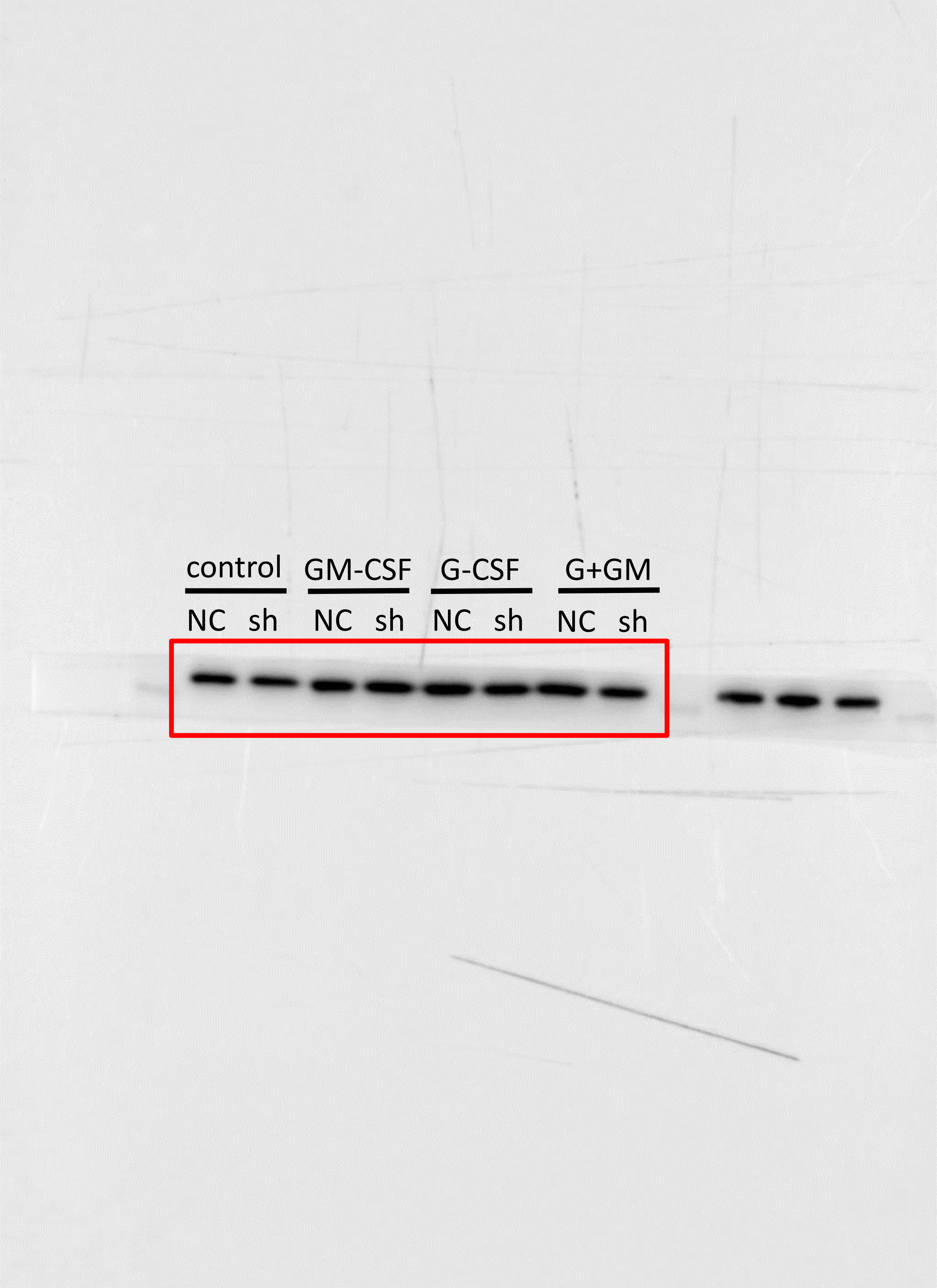

Figure 5F

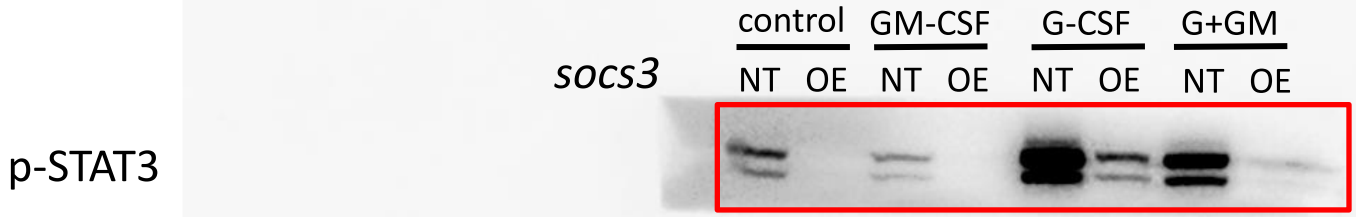

Figure 5F

STATA3

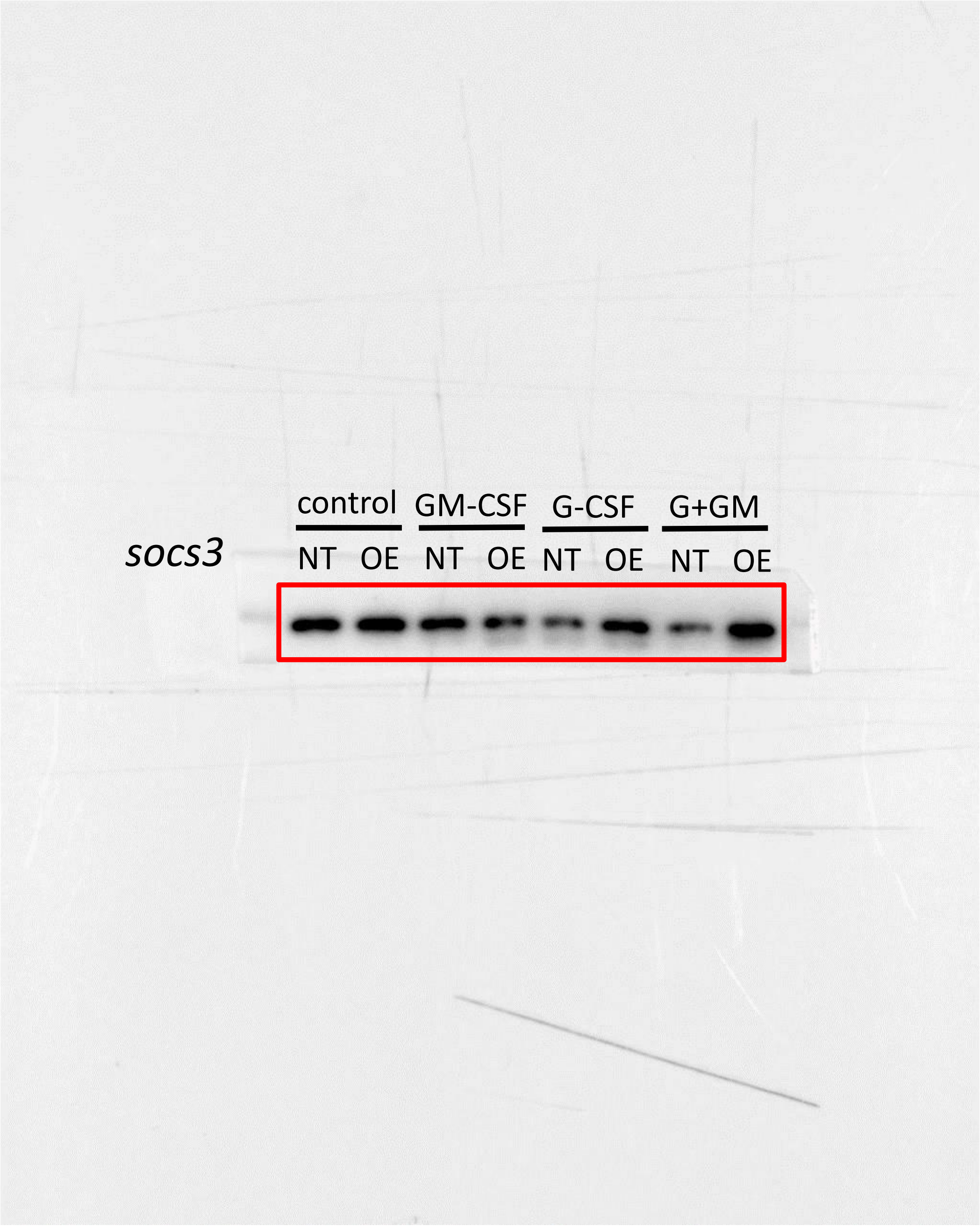

Figure 5F

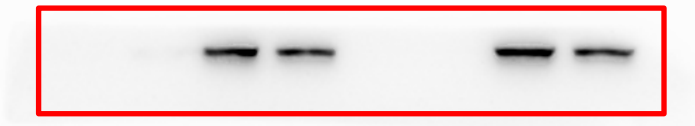

Figure 5F

STATS5

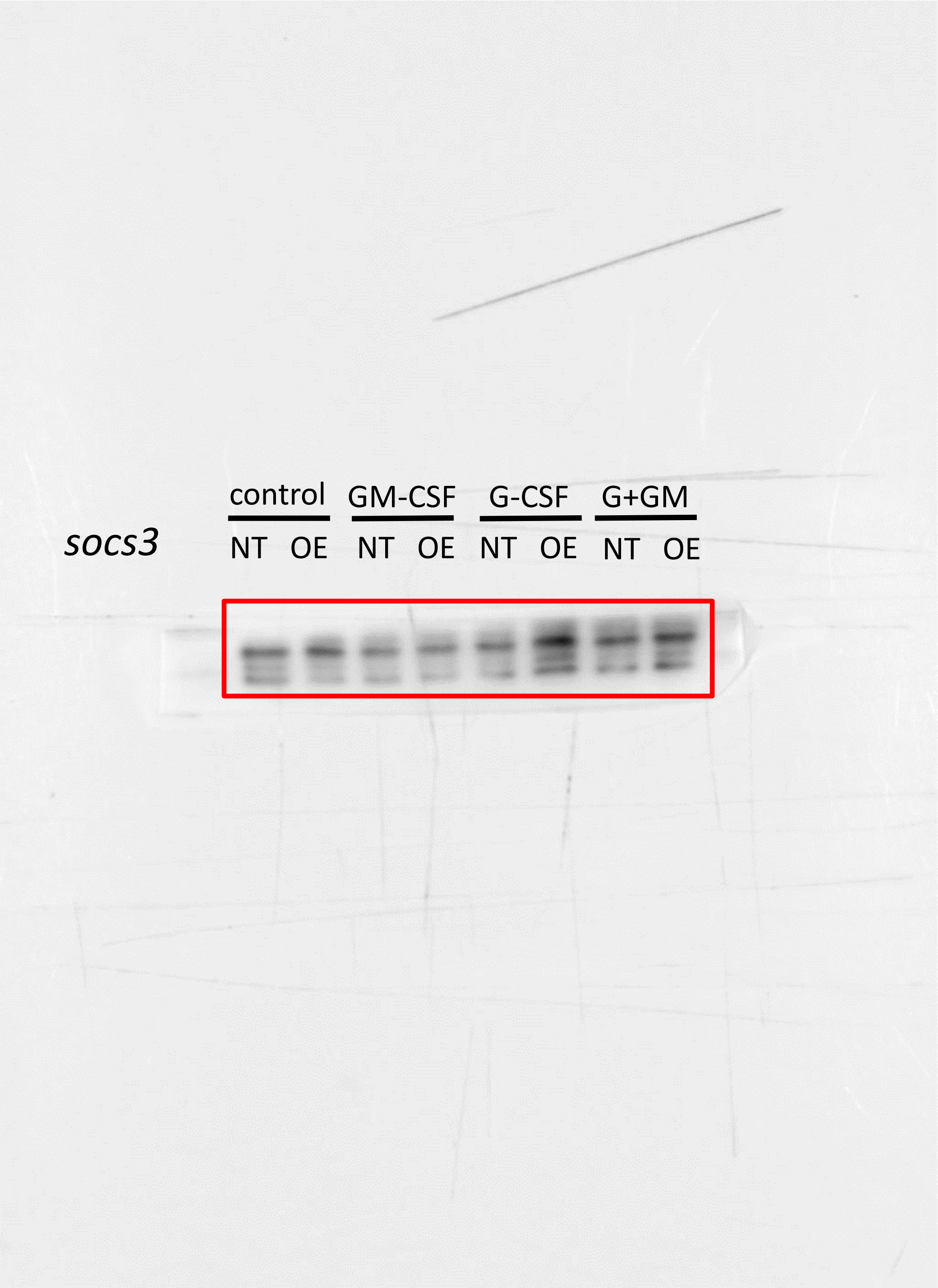

Figure 5F

ACTB

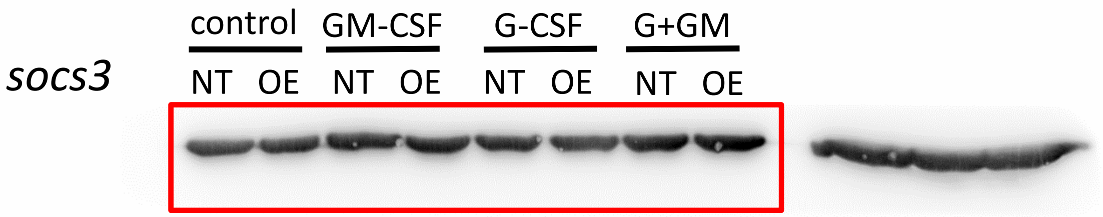

Figure 7

Figure 7A

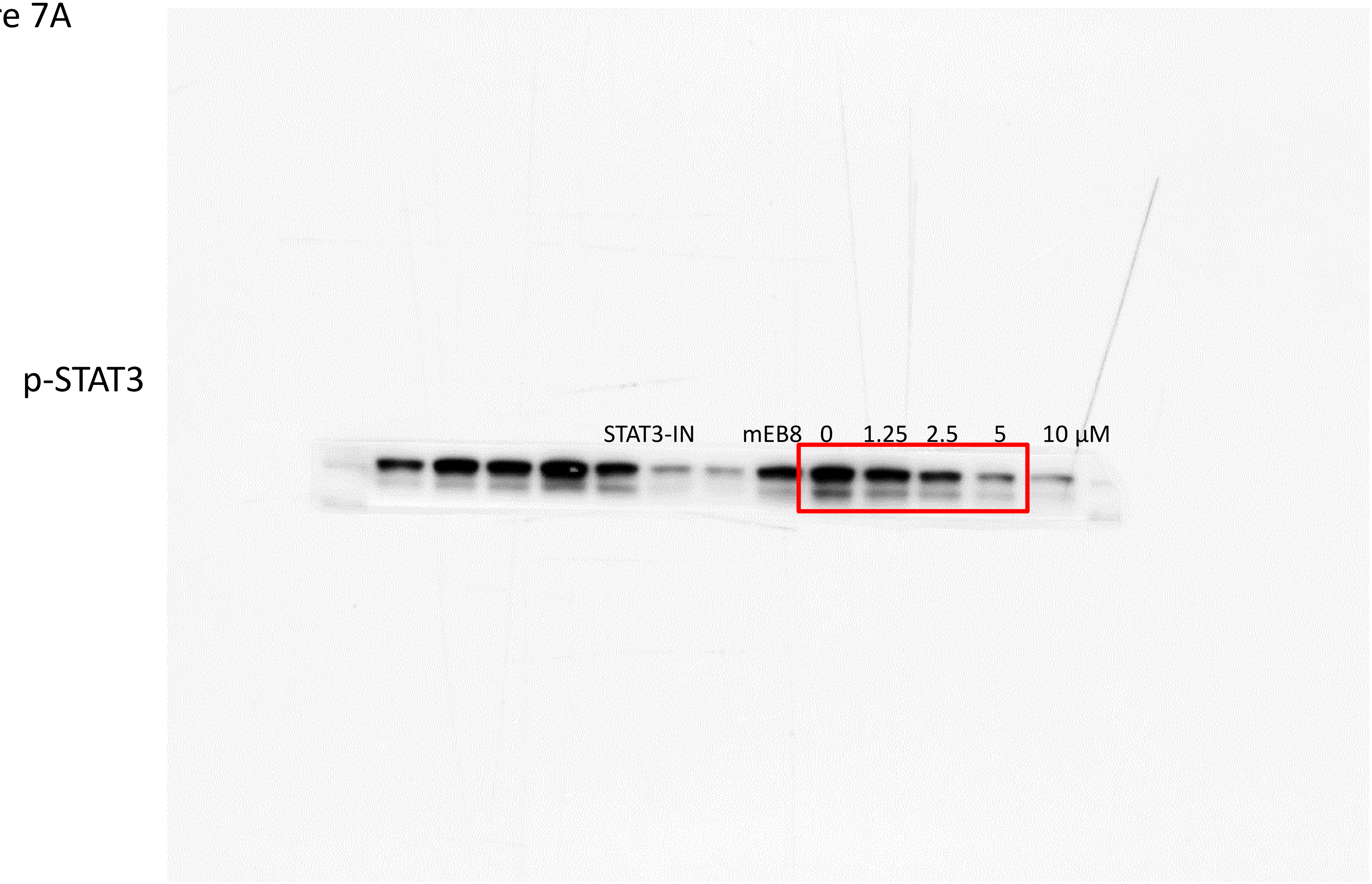

Figure 7A

STAT3

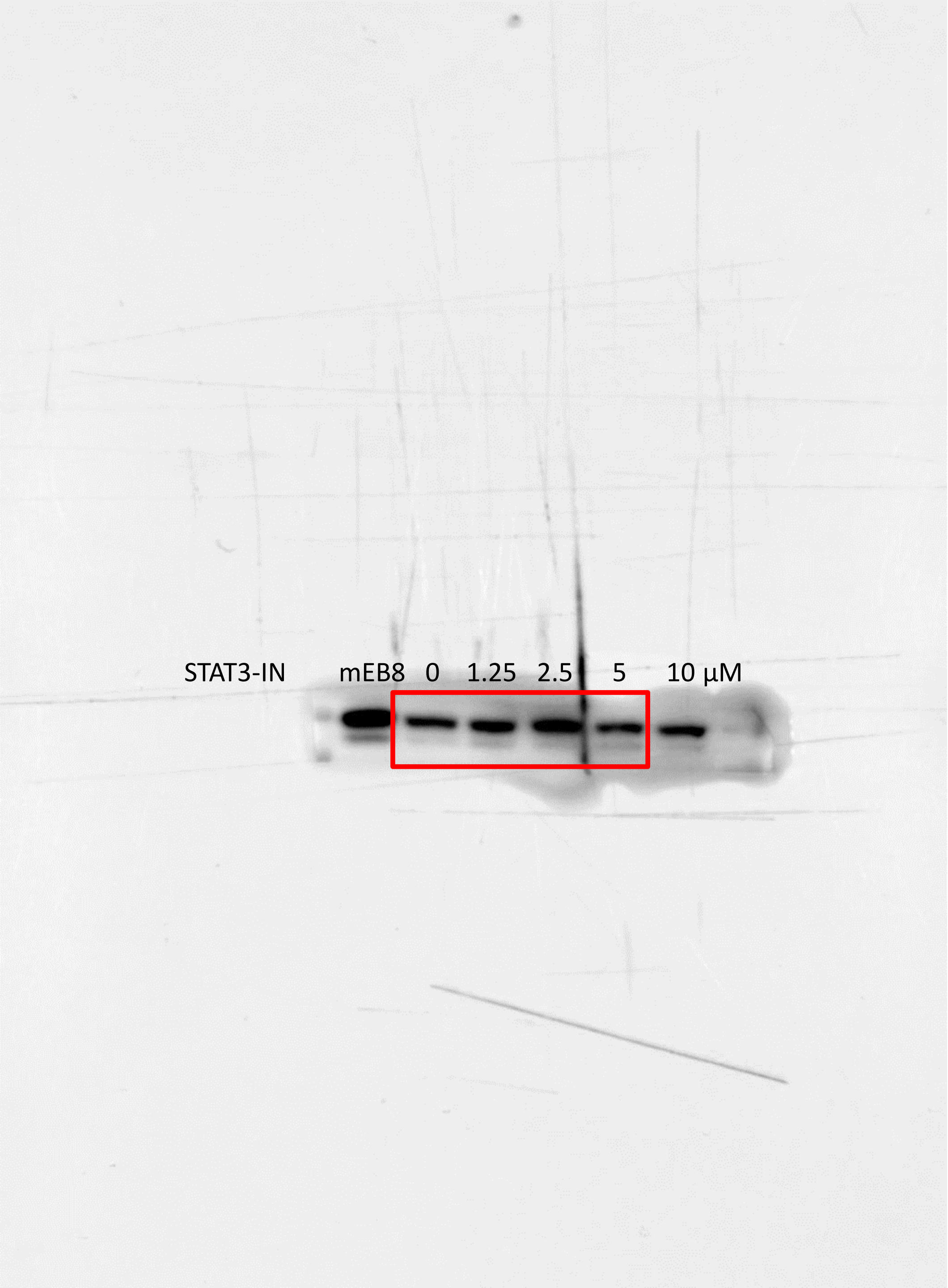

Figure 7A

p-STAT5

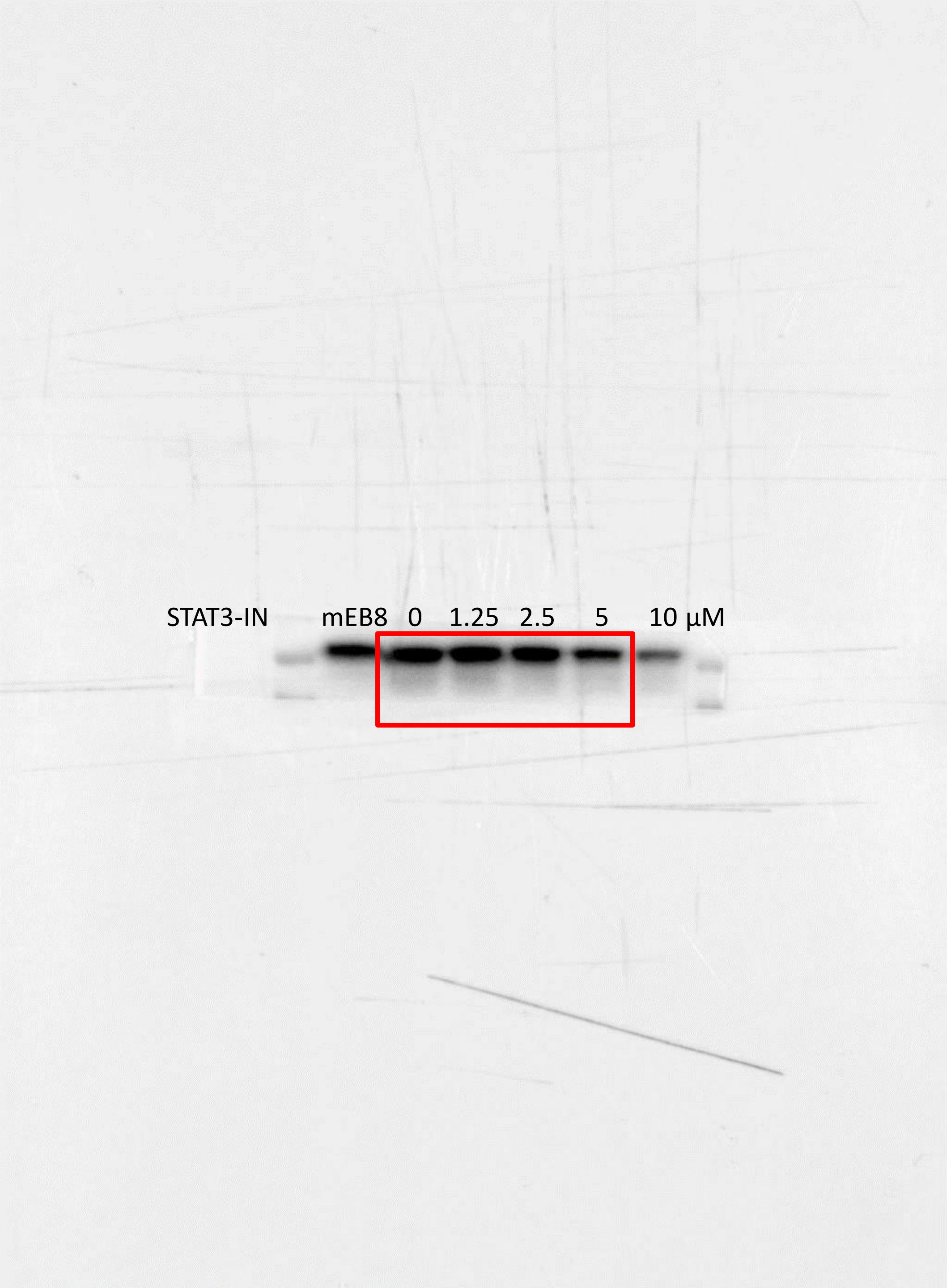

Figure 7A

STAT5

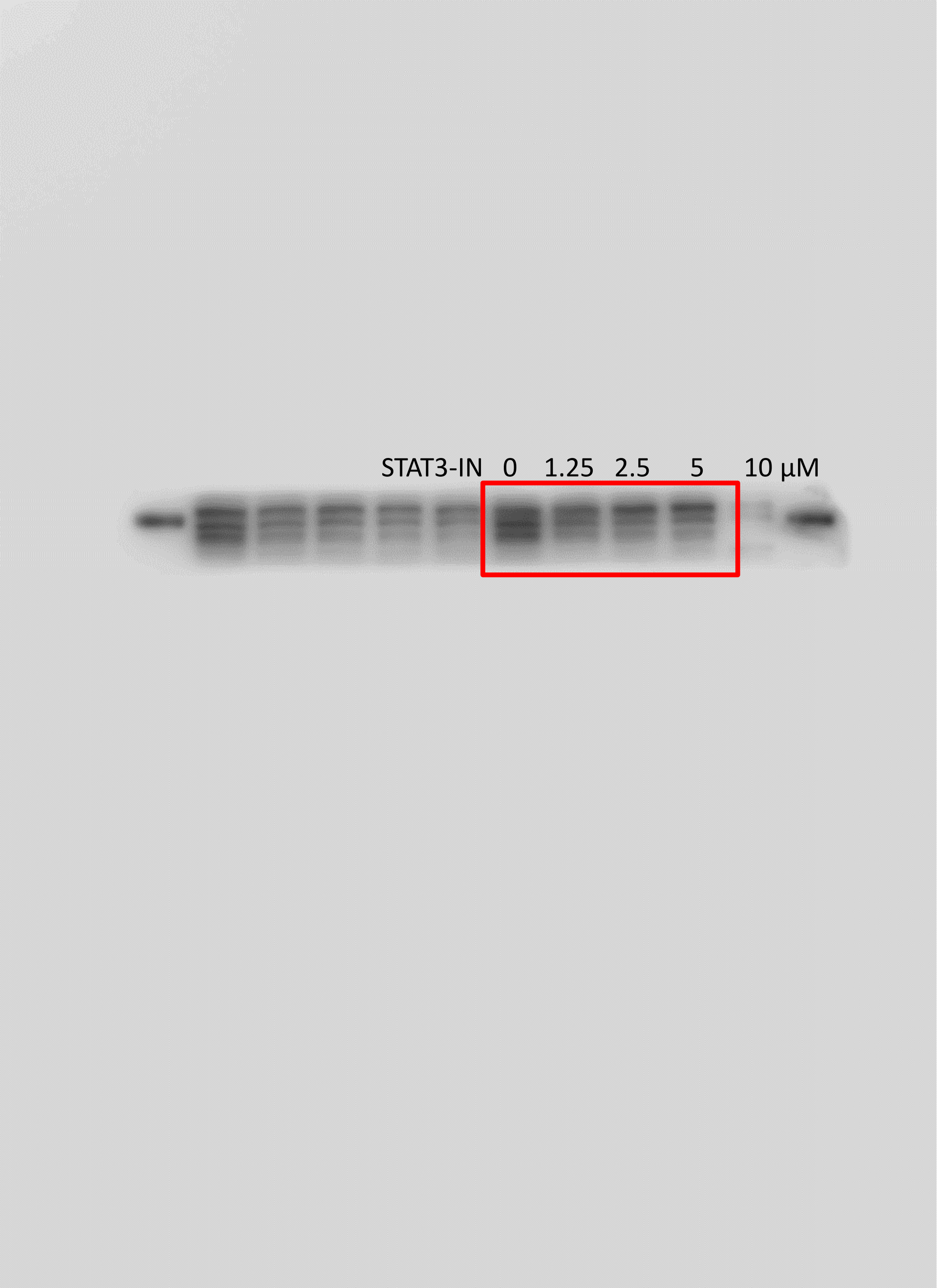

Figure 7A

ACTB

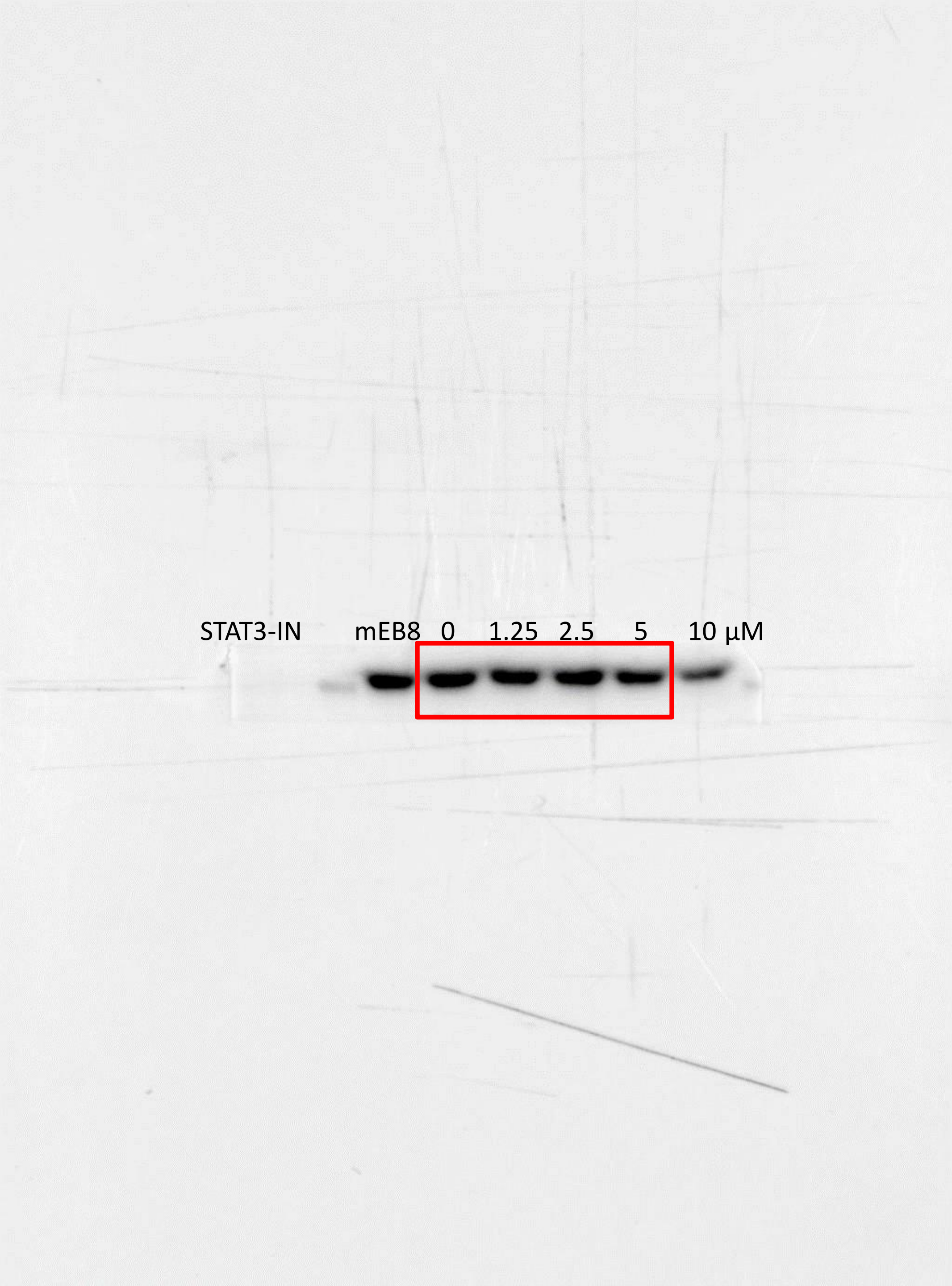

Figure 7B

p-STAT5

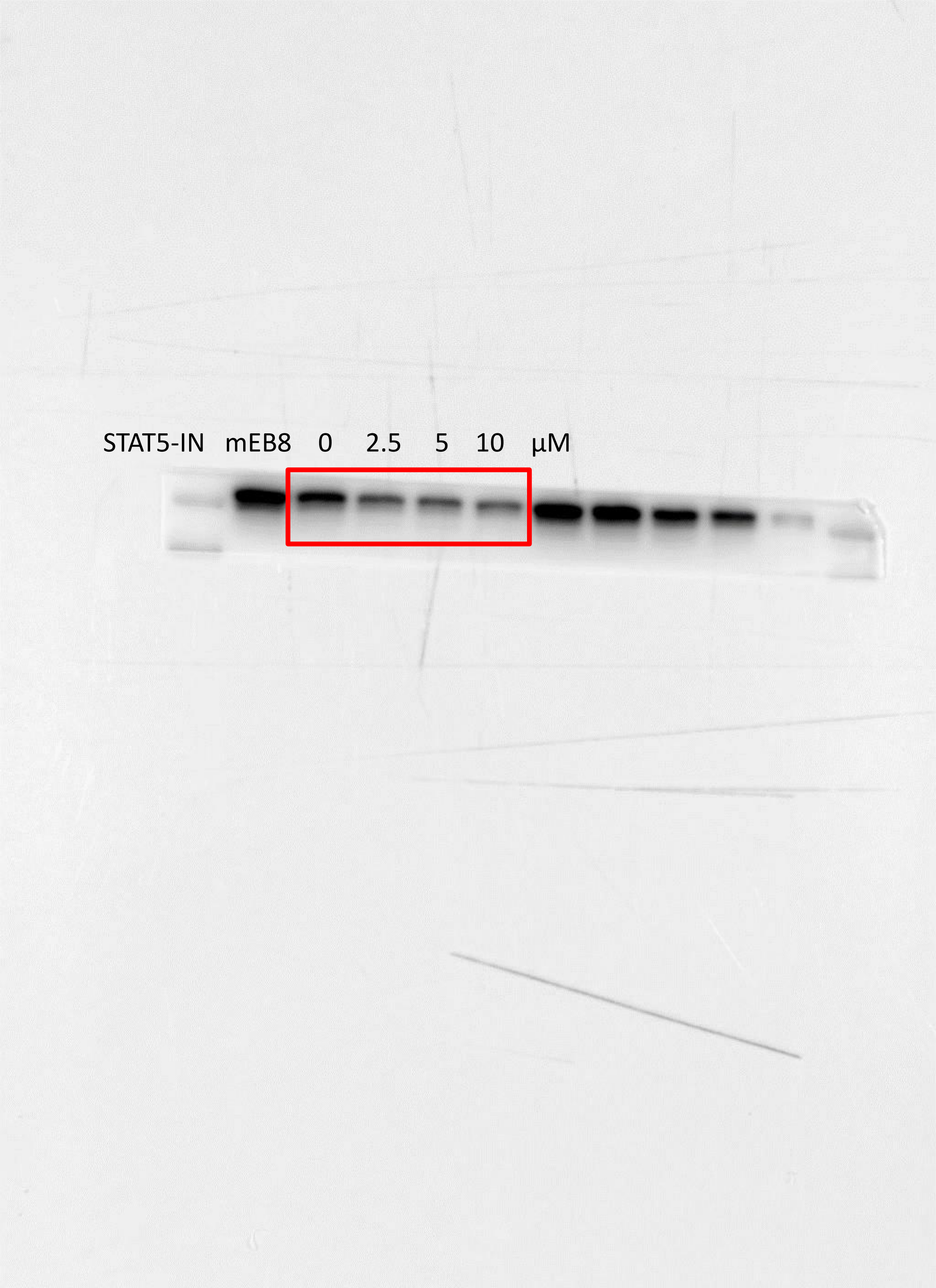

Figure 7B

STAT5

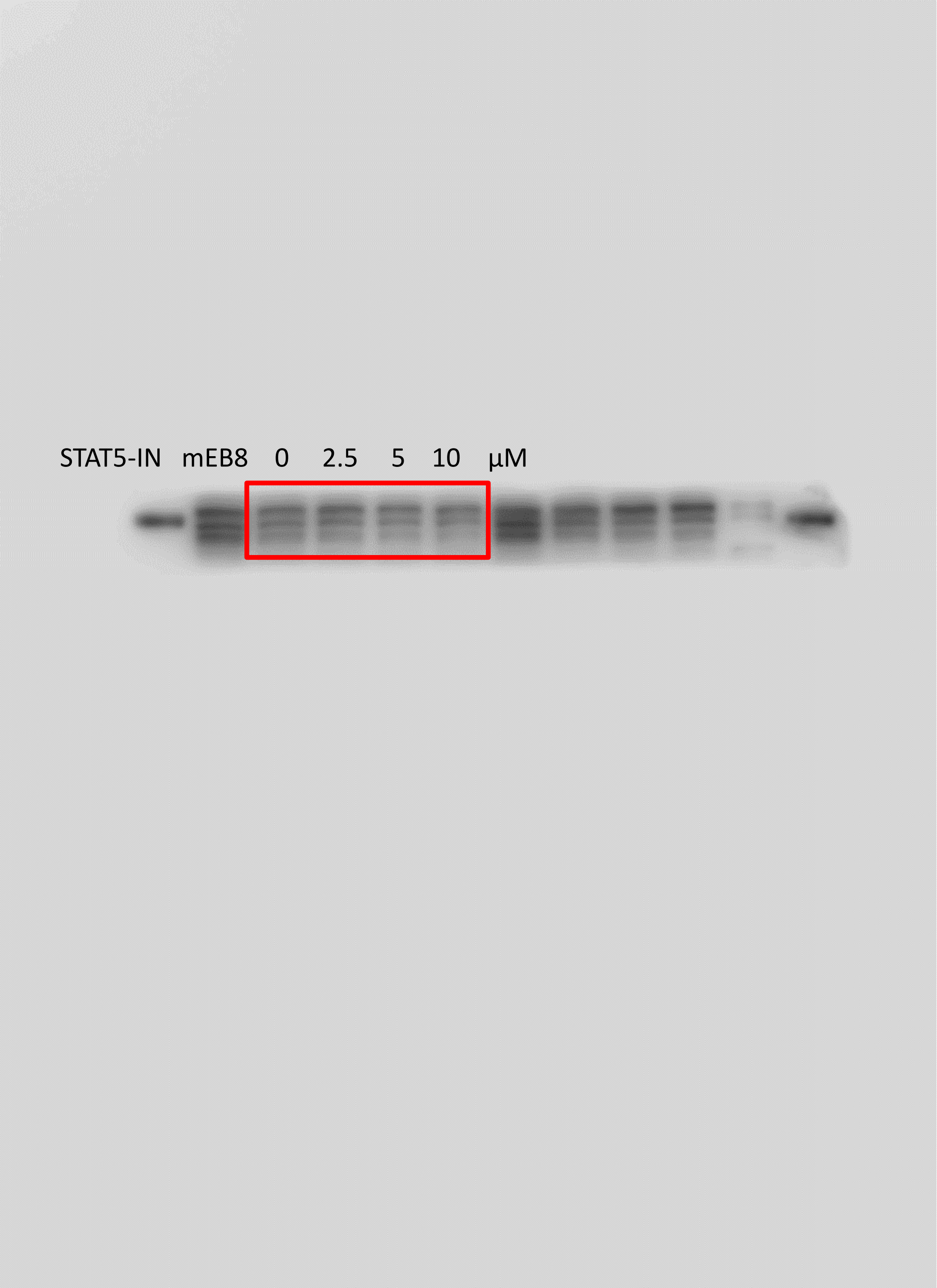

Figure 7B

p-STAT3

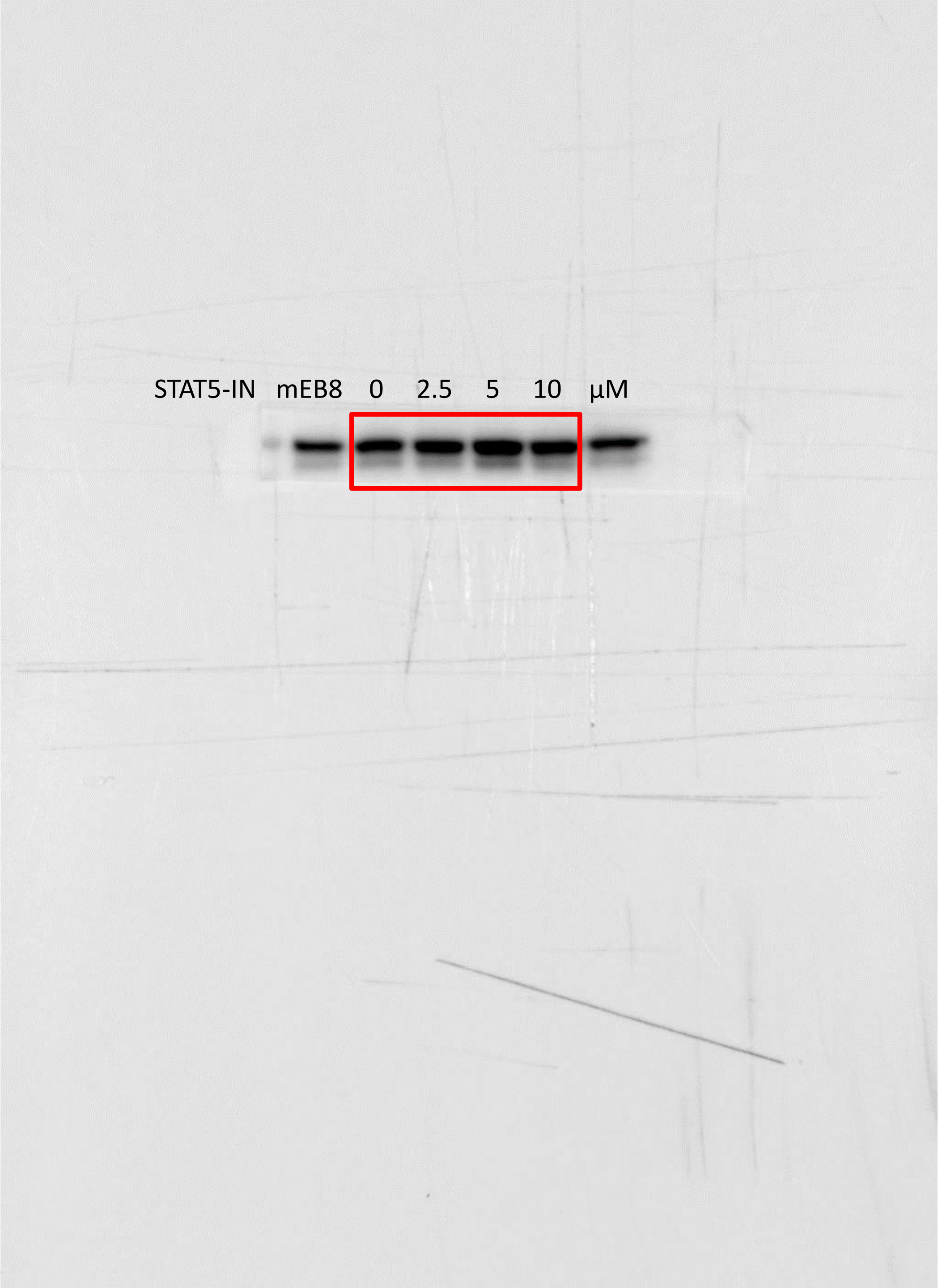

Figure 7B

STAT3

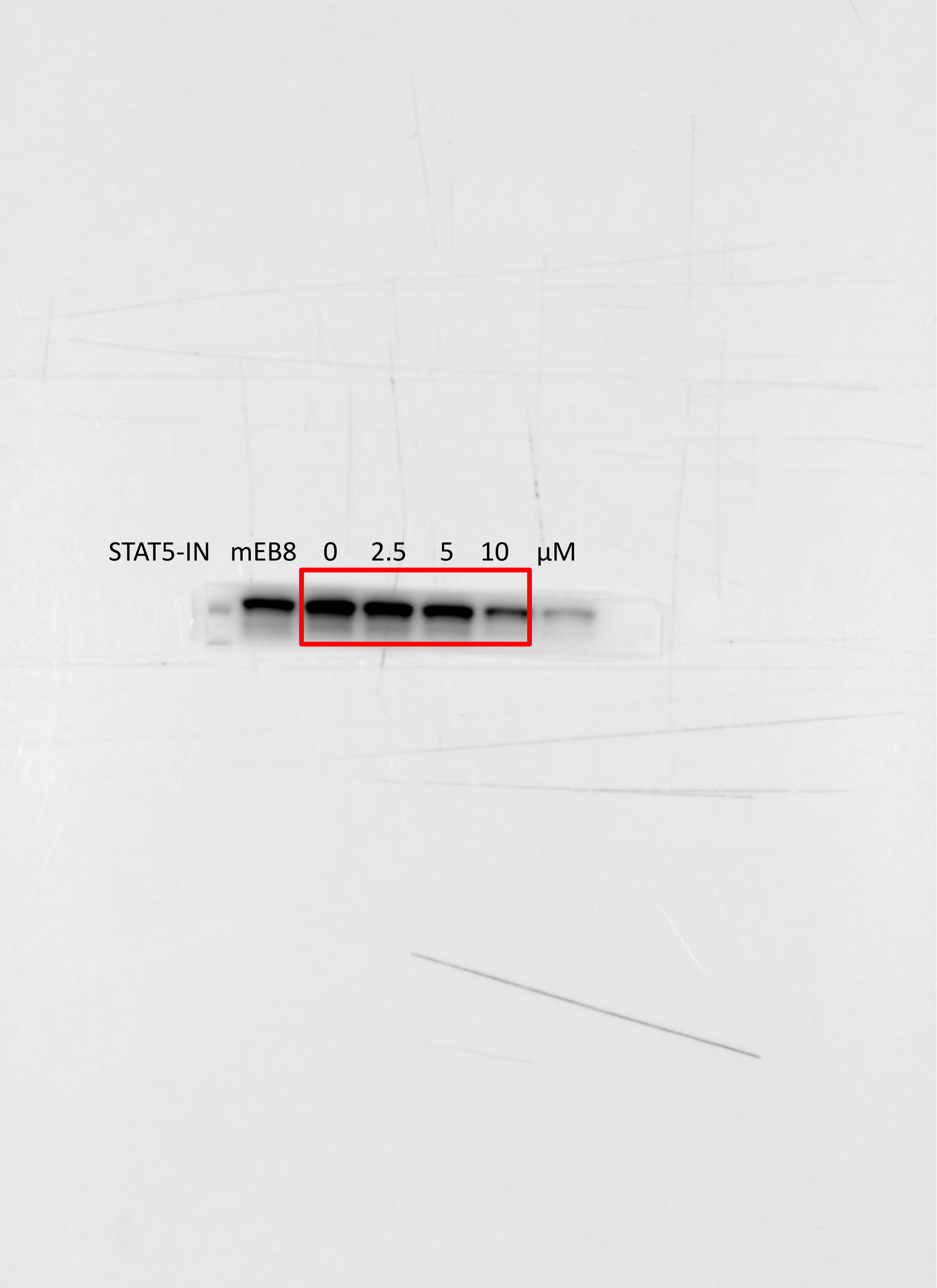

Figure 7B

ACTB

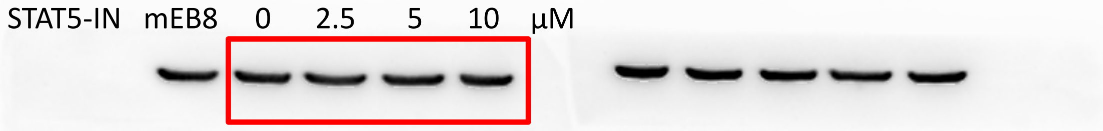

Figure S2

Figure S2A

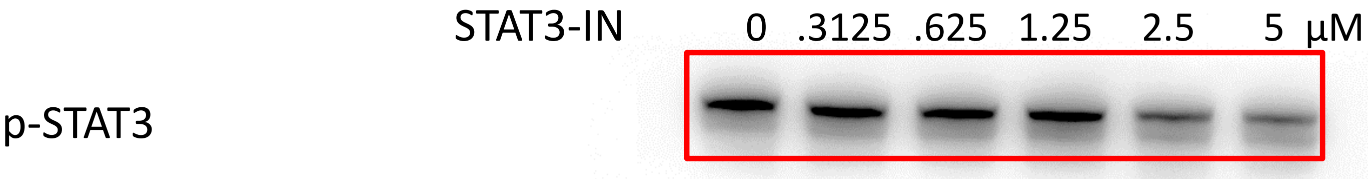

Figure S2A

STAT3

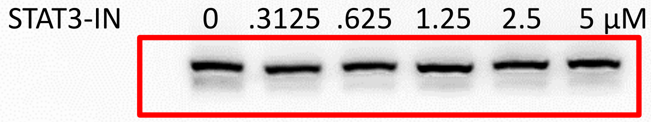

Figure S2A

STAT3-IN 0 .3125 .625 1.25 2.5 5 µM

p-STAT5

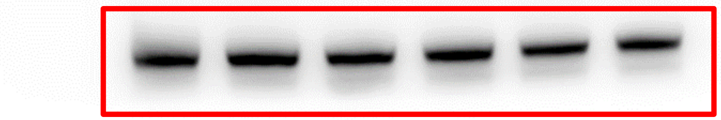

Figure S2A

STAT5

STAT3-IN 0 .3125 .625 1.25 2.5 5 µM

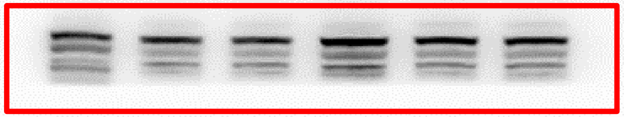

Figure S2A

ACTB

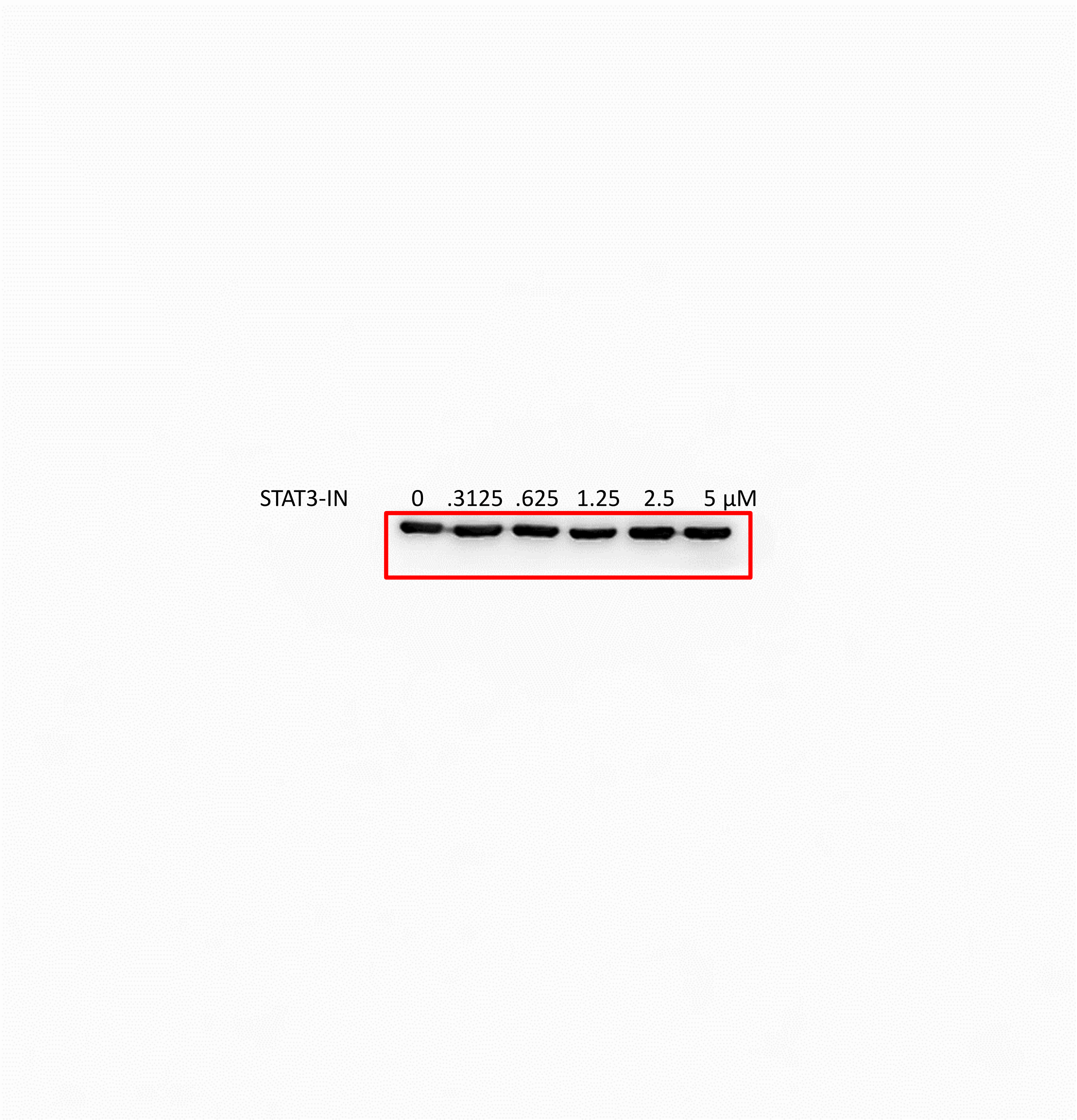

Figure S2B

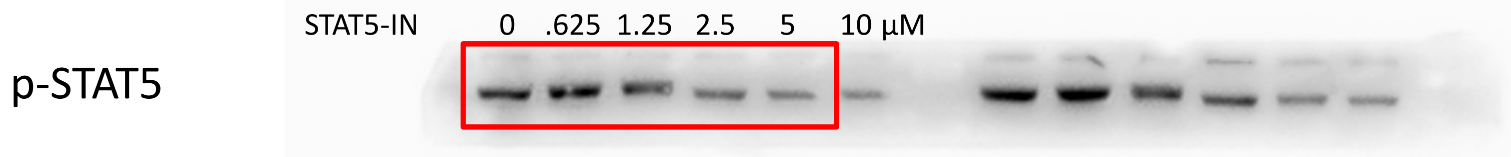

Figure S2B

STAT5

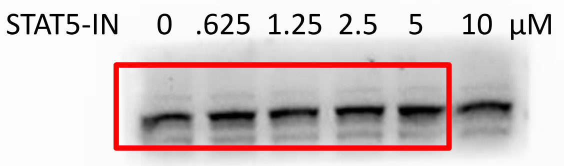

Figure S2B

STAT5-IN 0 .625 1.25 2.5 5 10 µM

p-STAT3

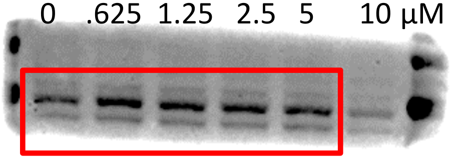

Figure S2B

STAT3

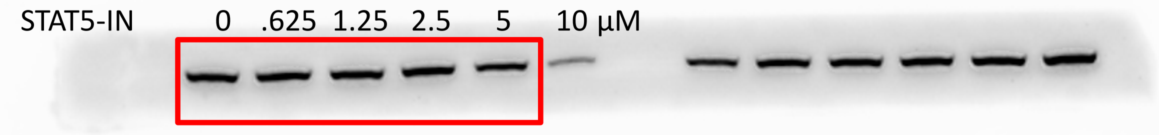

Figure S2B

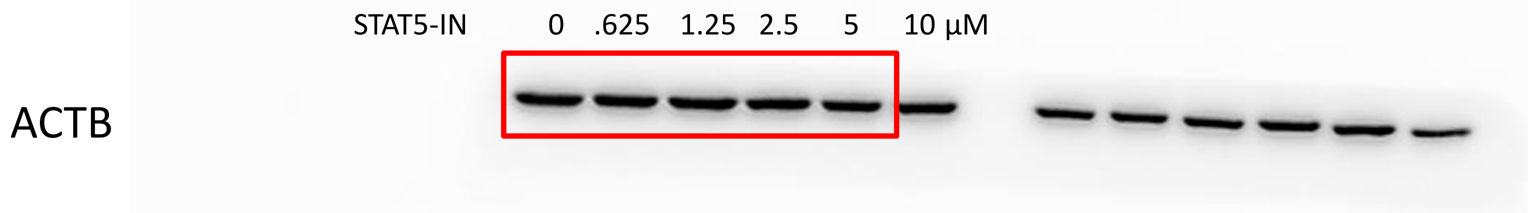

Figure S2C

STAT3

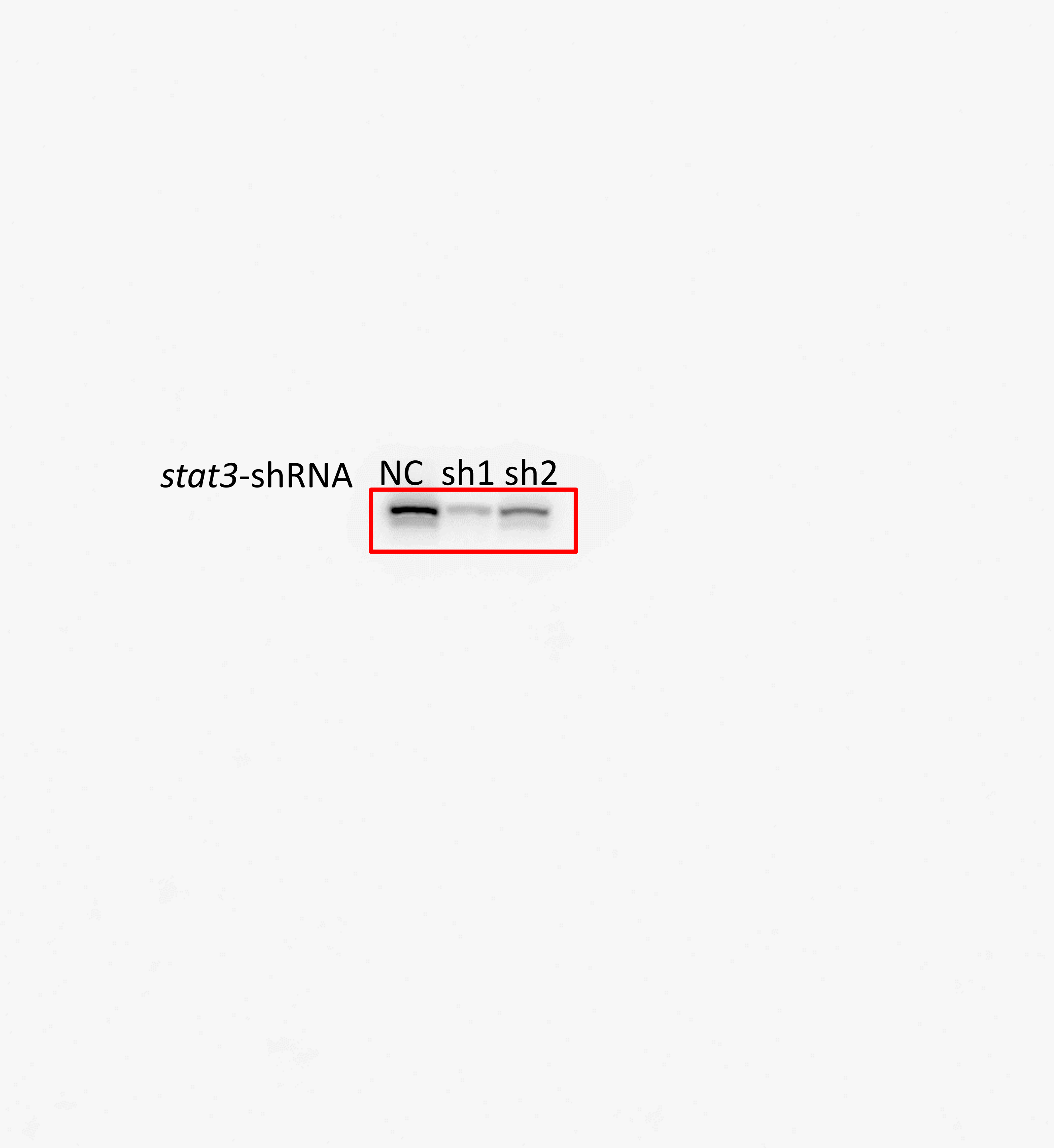

Figure S2C

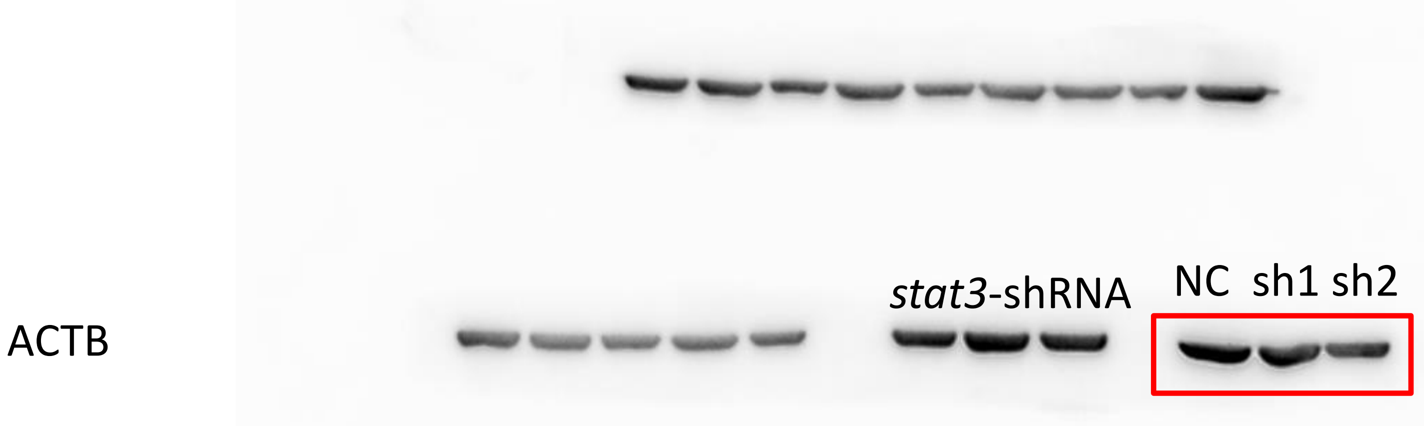

Figure S2D

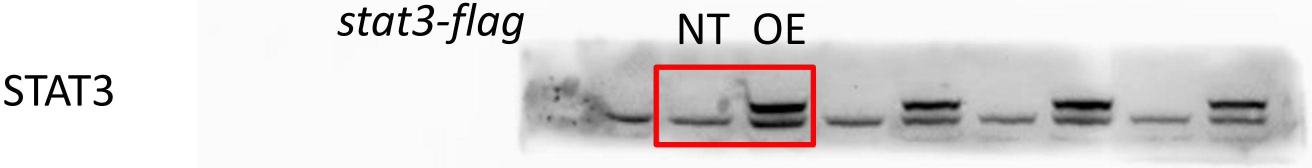

Figure S2D

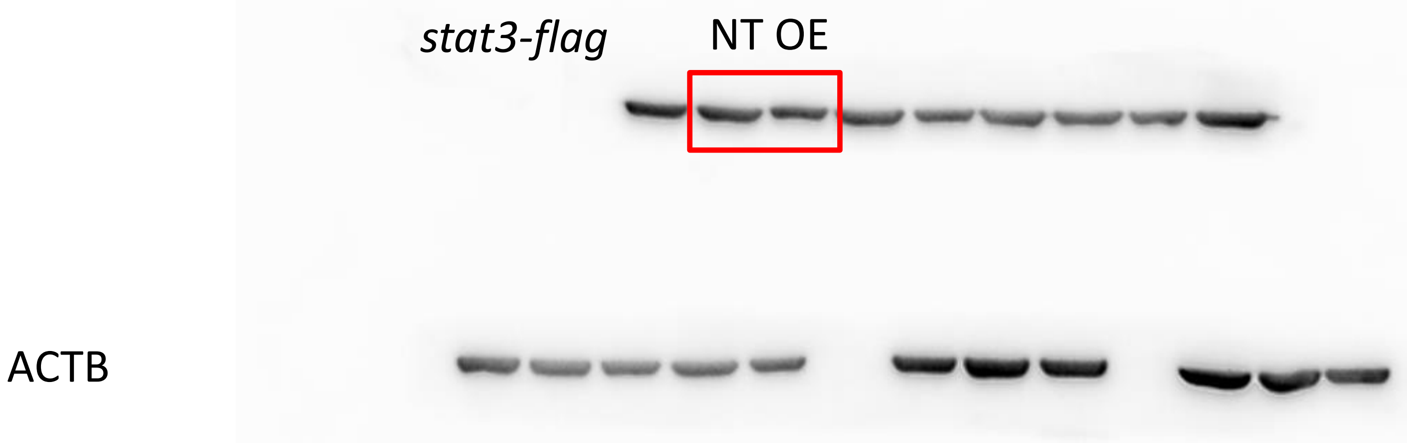

Figure S2E

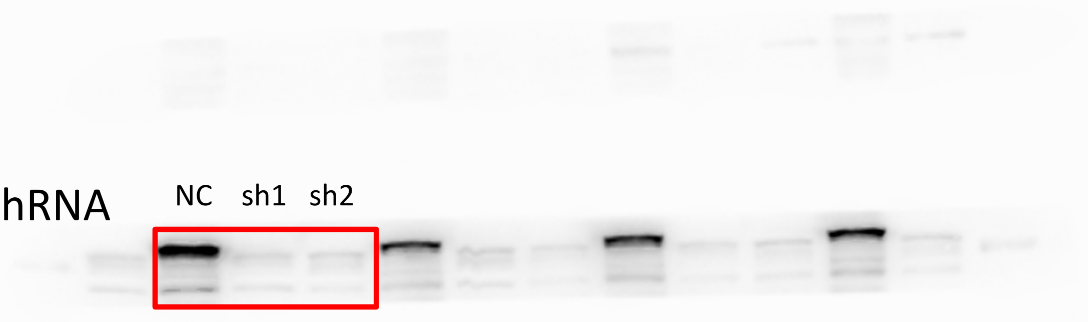

Figure S2E

STAT5

stat5-shRNA

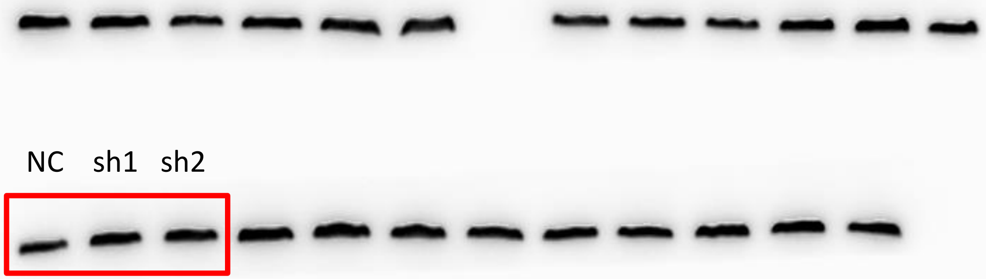

Figure S2F

STAT5

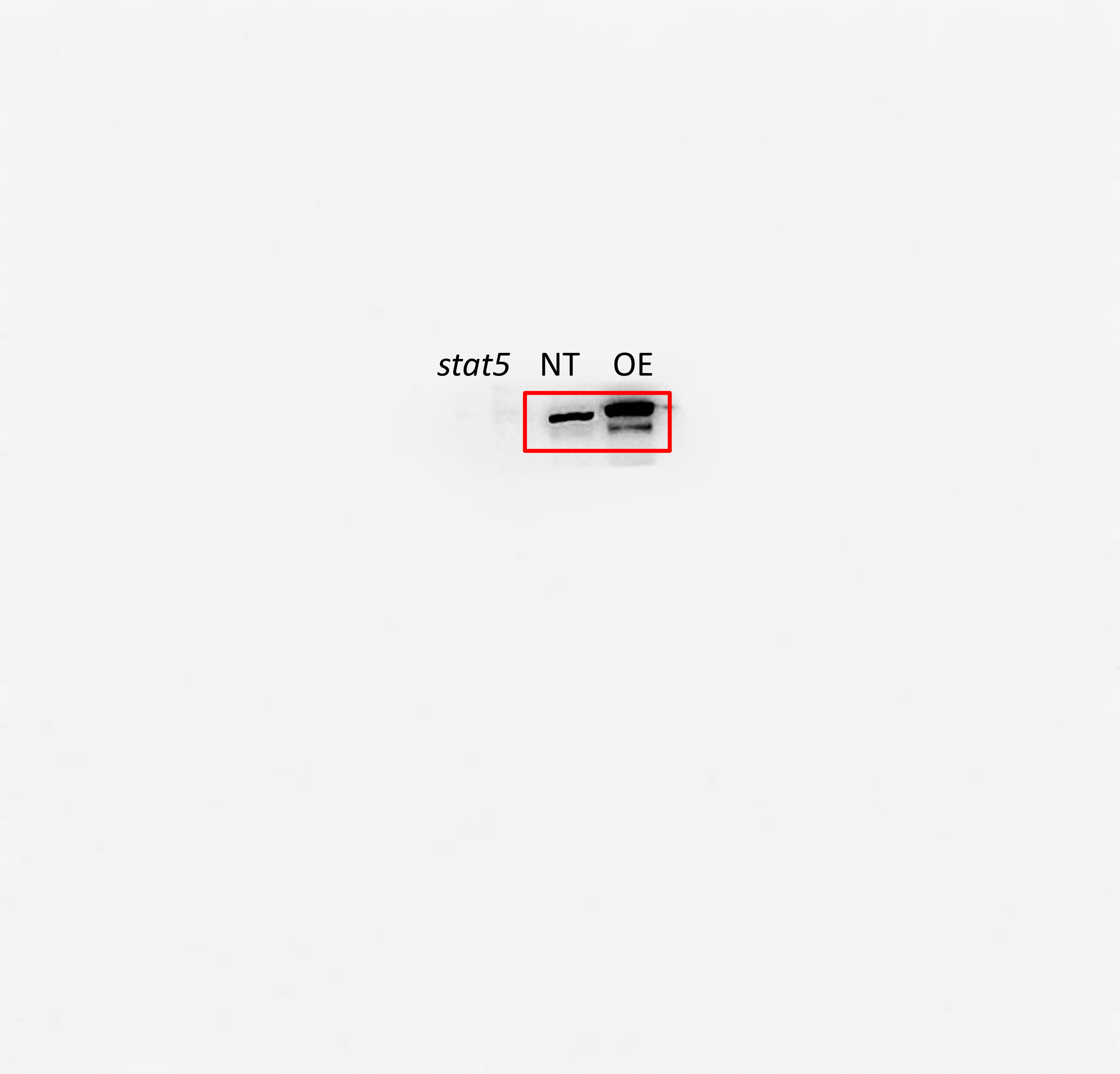

Figure S2F

ACTB

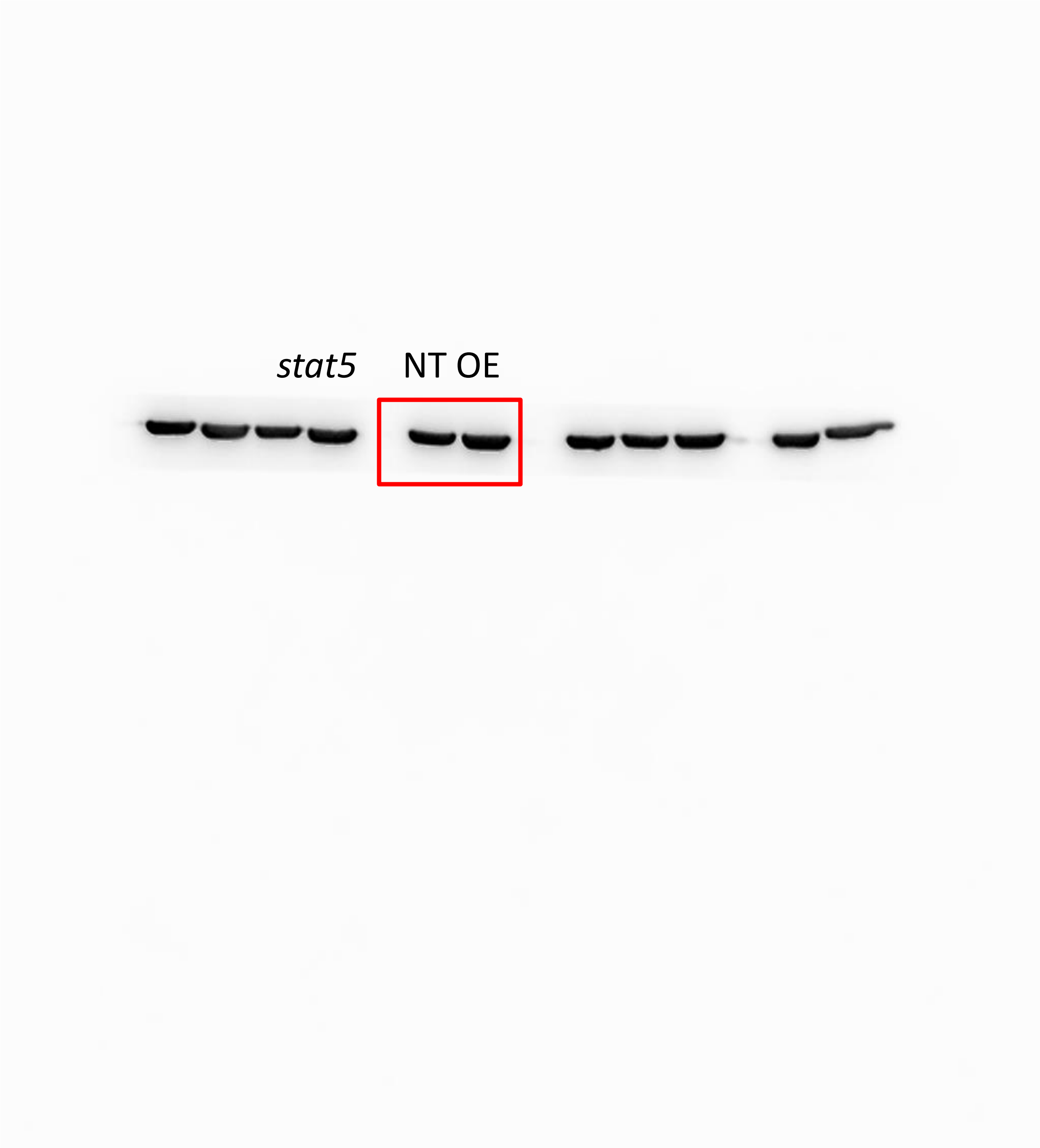

Figure S2G

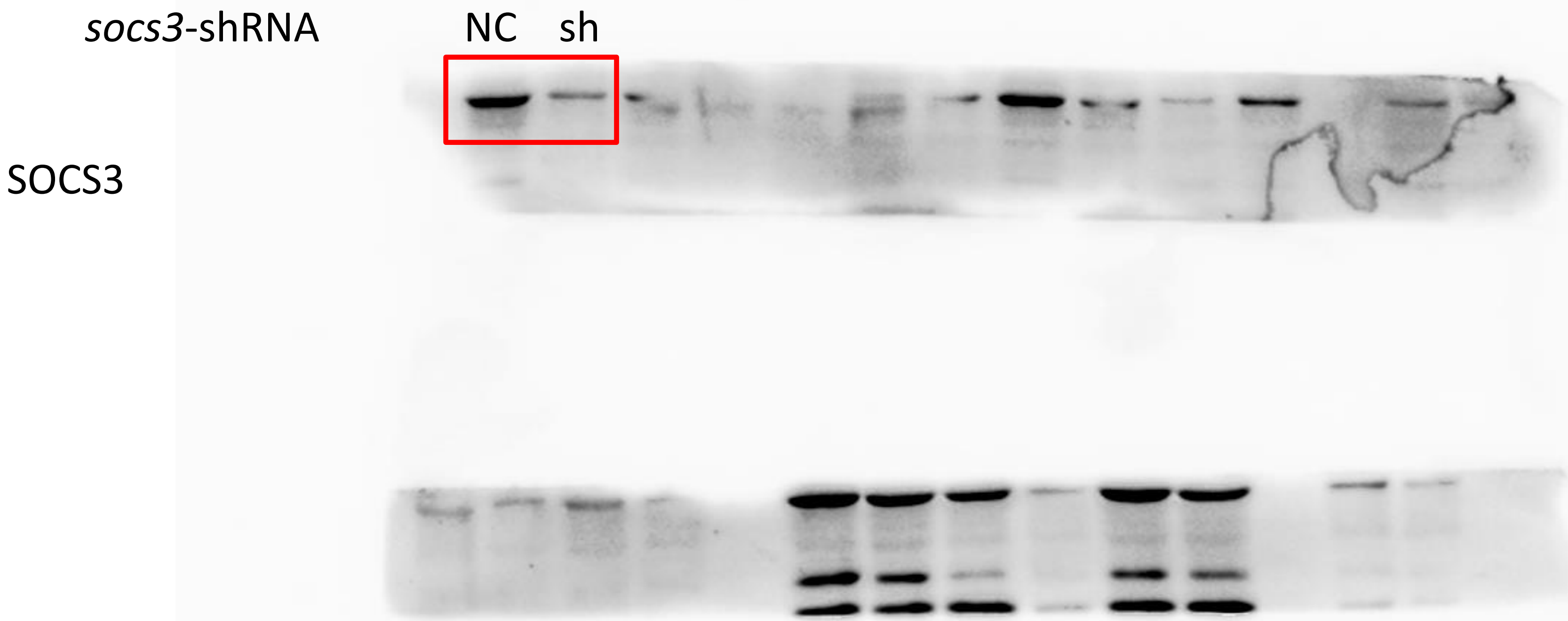

Figure S2G

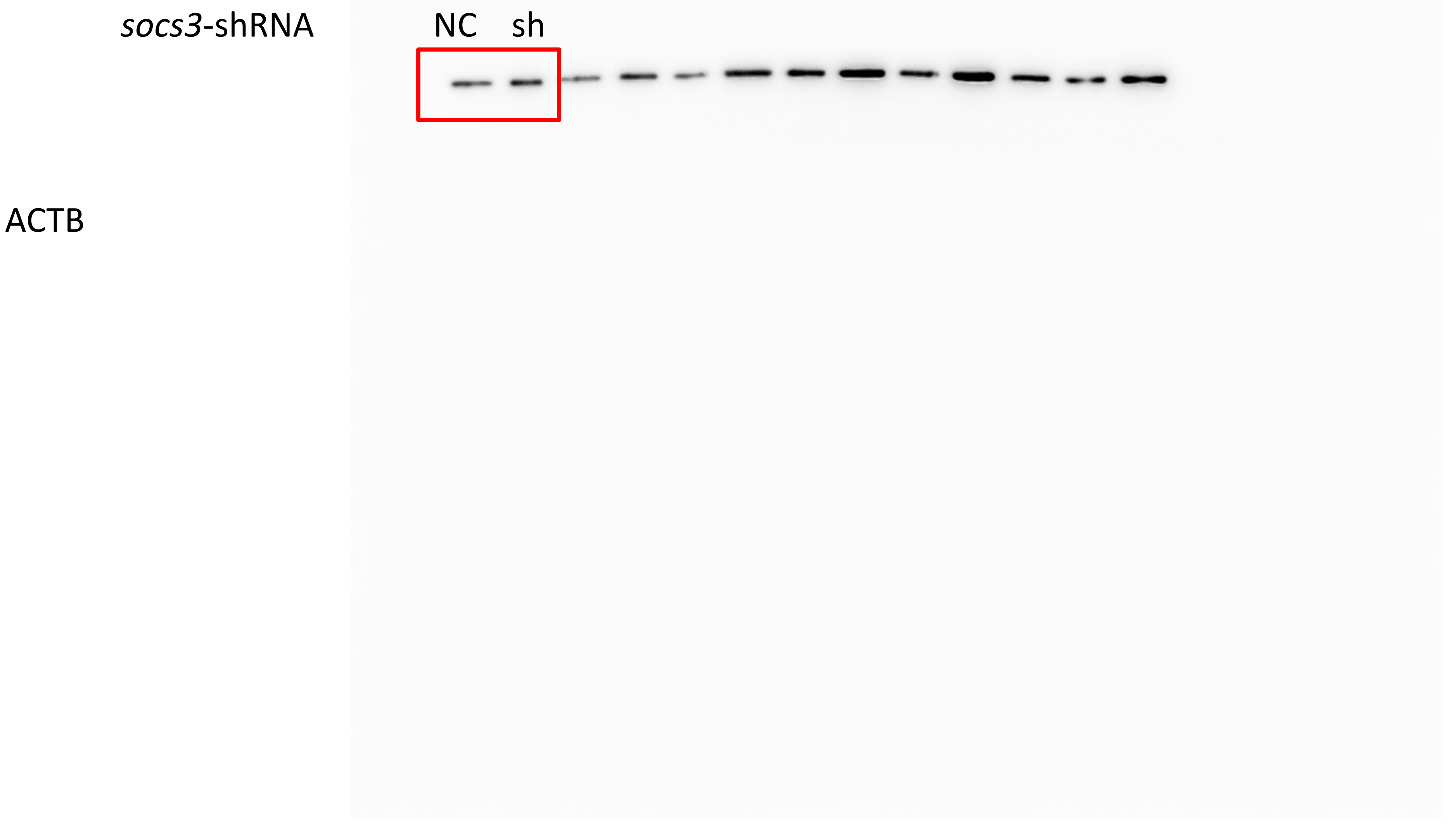

Figure S2H

SOCS3

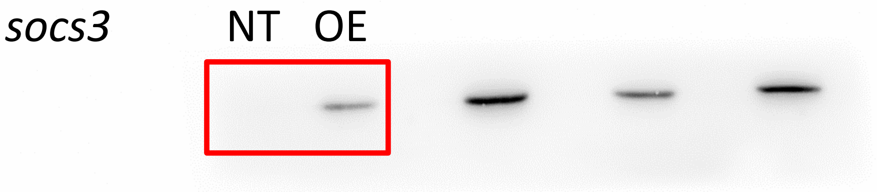

Figure S2H

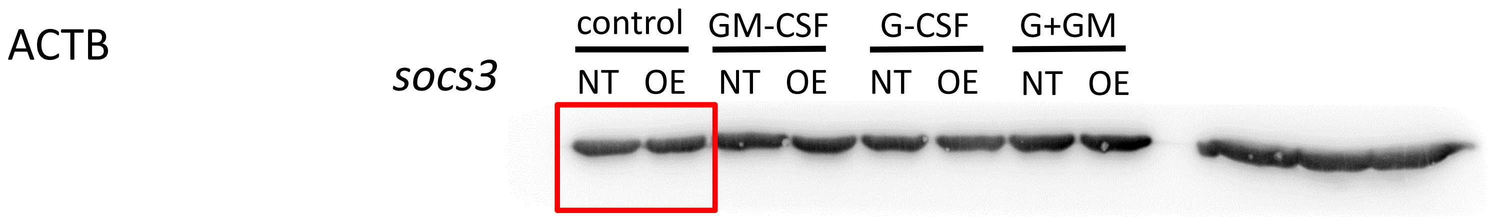

Figure S8

Figure S8A

p-STAT5

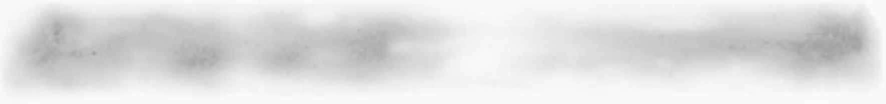

Figure S8A

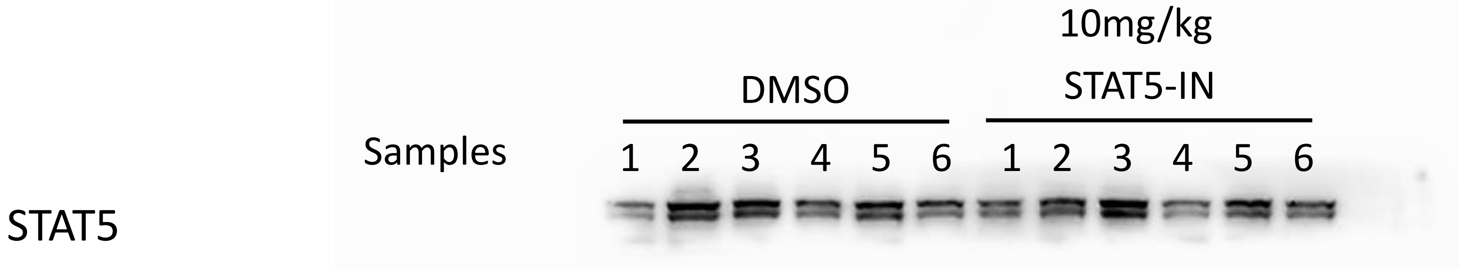

Figure S8A

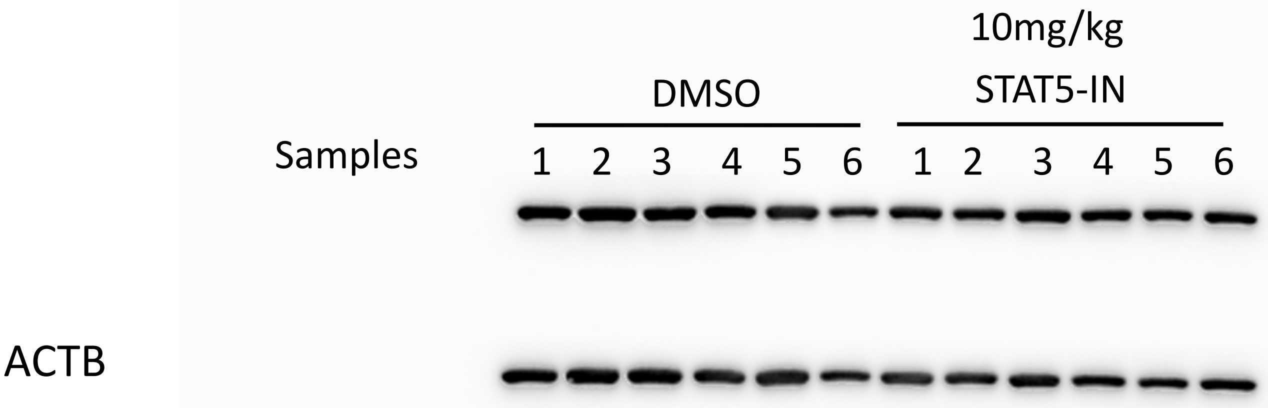

Figure S8A

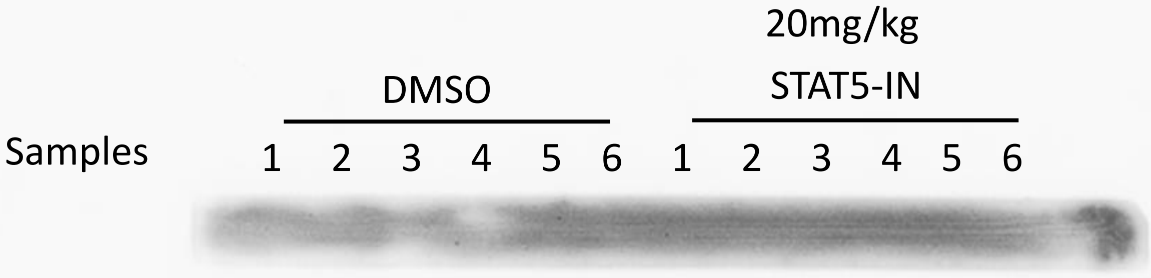

Figure S8A

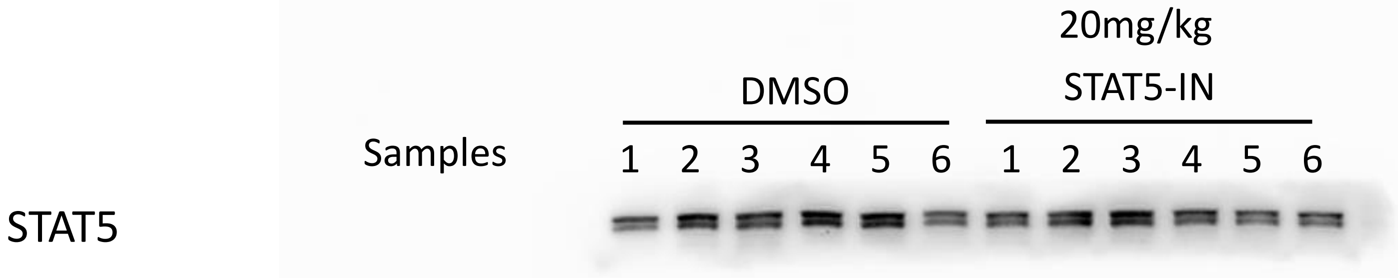

Figure S8A

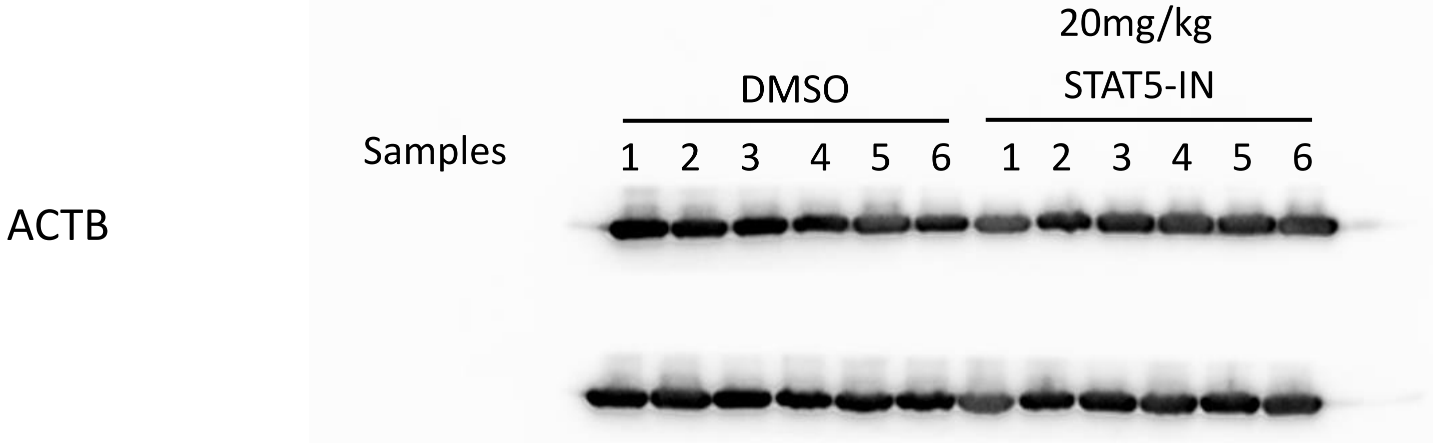

Supplement: Supplementary file 3 — Original Data File [file 41420_2023_1575_MOESM3_ESM.pdf]
